# Isolation of Lobane and Prenyleudesmane Diterpenoids from the Soft Coral *Lobophytum varium*

**DOI:** 10.3390/md18040223

**Published:** 2020-04-22

**Authors:** Chuan-Hsiang Chang, Atallah F. Ahmed, Tian-Sheng Yang, You-Cheng Lin, Chiung-Yao Huang, Tsong-Long Hwang, Jyh-Horng Sheu

**Affiliations:** 1 Department of Marine Biotechnology and Resources, National Sun Yat-sen University, Kaohsiung 804, Taiwan; hunter02141991@gmail.com (C.-H.C.); b025020004@student.nsysu.edu.tw (T.-S.Y.); betty8575@yahoo.com.tw (C.-Y.H.); 2 Department of Pharmacognosy, College of Pharmacy, King Saud University, Riyadh 11451, Saudi Arabia; afahmed@ksu.edu.sa; 3 Department of Pharmacognosy, Faculty of Pharmacy, Mansoura University, Mansoura 35516, Egypt; 4 Doctoral Degree Program in Marine Biotechnology, National Sun Yat-sen University, Kaohsiung 804, Taiwan; cand93@yahoo.com.tw; 5 Graduate Institute of Natural Products, College of Medicine, Chang Gung University, Taoyuan 333, Taiwan; htl@mail.cgu.edu.tw; 6 Research Center for Chinese Herbal Medicine, Research Center for Food and Cosmetic Safety, and Graduate Institute of Health Industry Technology, College of Human Ecology, Chang Gung University of Science and Technology, Taoyuan 333, Taiwan; 7 Department of Anesthesiology, Chang Gung Memorial Hospital, Taoyuan 333, Taiwan; 8 Department of Medical Research, China Medical University Hospital, China Medical University, Taichung 404, Taiwan; 9 Graduate Institute of Natural Products, Kaohsiung Medical University, Kaohsiung 807, Taiwan; 10 Frontier Center for Ocean Science and Technology, National Sun Yat-sen University, Kaohsiung 804, Taiwan

**Keywords:** lobane, prenyleudesmane, anti-inflammatory activity, *Lobophytum varium*

## Abstract

Further chemical investigation of the EtOAc extract of the soft coral *Lobophytum varium* resulted in the discovery of eleven new diterpenoids lobovarols F–P (**1**–**11**) of lobane– and prenyleudesmane–types, along with two known metabolites (**12** and **13**). The structures of the new metabolites were established by spectroscopic analyses, including 2D NMR experiments. The absolute configuration of **1** was determined using Mosher’s method. The complete assignment of ^1^H and ^13^C NMR spectroscopic data of **12** and **13** and the identification of pyran-derived moieties in the prenyleudesmanes were reported for the first time. Anti-inflammatory activities of the isolated compounds in suppressing elastase release and superoxide anion generation in human neutrophils were disclosed for **1**, **2**, **4**, **12**, and **13**. A stereospecific biosynthesis for lobanes and prenyleudesmanes from the related prenylgermacranes could explain the coexistence of lobanes and prenylgermacranes in *L. varium*.

## 1. Introduction

Soft corals belonging to genus *Lobophytum* (Alcyoniidae) were shown to be the main biological sources of lobane [1,2,3,4,5,6,7], prenyleudesmane [1,8,9,10,11], and spatane–type diterpenoids [8]. Some lobane and prenyleudesmane diterpenoids have displayed antimicrobial [2], cytotoxic [2,6,8], and anti-inflammatory activities [1]. Cembranoids have also been isolated from this genus, and have been shown to exhibit significant cytotoxic [12,13,14], antiviral [15], and anti-inflammatory activities [14,16,17,18]. Our previous chemical study on the EtOAc extract of a Formosan soft coral, *Lobophytum varium* has led to the discovery of five new metabolites of lobane- and prenyleudesmane-types, of which some were found to possess anti-inflammatory activity through the suppression of elastase release and/or superoxide anion generation in challenged neutrophils [1]. The present study was aimed to further discover new bioactive diterpenoids from continuing investigation of the organic fractions of *L. varium*. This study not only led to the isolation of new metabolites but also gave strong evidence for the biosynthetic mechanism of lobane and prenyleudesmane from the corresponding prenylgermacrane biosynthetic intermediate was proposed, which could explain the common coexistence of lobane and prenylgermacranes in *L. varium*.

## 2. Results and Discussion

The terpenoids containing fractions of the EtOAc extract of *L. varium* were repeatedly purified using a series of chromatographic techniques, including HPLC, to afford compounds **1**–**13** (Figure 1). The structures of the new compounds (**1**–**11**) were established on the basis of NMR data (Table 1, Table 2 and Table 3) and spectroscopic analyses (Supplementary Materials, Figures S1 to S79).

**Figure 2:**
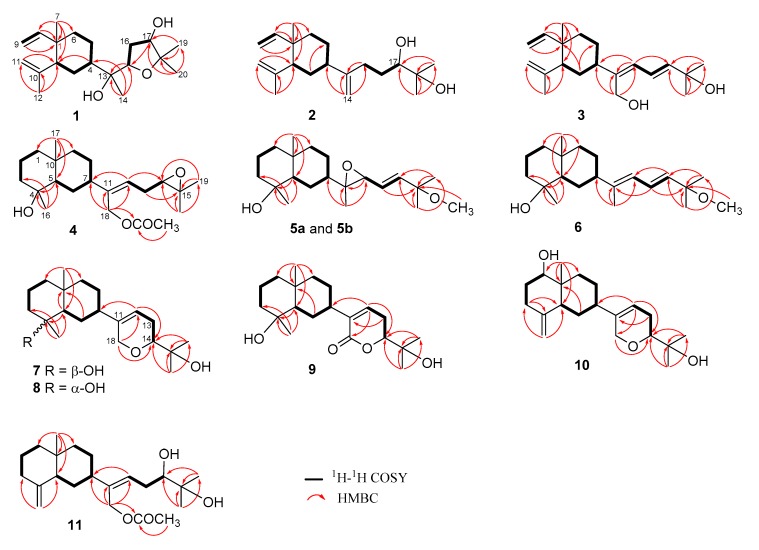
^1^H–^1^H COSY and HMBC correlations of **1**–**11**.

Lobovarol F (**1**) was found to possess a molecular formula C_20_H_34_O_3_ as established by high-resolution electrospray ionization mass spectrometry (HRESIMS, *m*/*z* 345.2403, [M + Na]^+^), indicating four degrees of unsaturation. The IR absorption at 3393 cm^−1^ designated the presence of the hydroxy group. The NMR signals (Table 1) showed the presence of a vinyl (*δ*_C_ 150.0, CH and 110.0, CH_2_; *δ*_H_ 5.80, dd, *J* = 18.0, 10.4 Hz, 4.90 d, *J* = 10.4 Hz, and 4.89, br d, *J* = 18.0 Hz), an isopropenyl (*δ*_C_ 147.6, C; 112.1, CH_2_ and 24.9, CH_3_; *δ*_H_ 4.83 and 4.59, each 1H, s; and 1.71, 3H, s); a ring-juncture methyl (*δ*_C_ 16.6, CH_3_; *δ*_H_ 0.99, 3H, s) and methine (*δ*_C_ 52.4, CH; *δ*_H_ 1.98, m) of a β-elemene segment of lobane-type diterpenoids [1,2,3]. Thus, the proton signals at *δ*_H_ 1.30, 1.26, and 1.15 (each 3H, s) should be the methyls attached to the oxygen-bearing carbons of the side chain. The analysis of correlations spectroscopy (COSY) and heteronuclear multiple bond correlation (HMBC) led to the establishment of a 2,2-dimethyl-3-hydroxytetrahydrofuran attached to a methyl-bearing oxygenated carbon (*δ*_C_ 75.0, C-13) to form the side chain of **1** (Figure 2). Accordingly, the HMBC correlations from H_3_-14 (*δ*_H_ 1.26) and from both H_3_-19 and H_3_-20 (*δ*_H_ 1.15 and 1.30) to the downfield shifted oxycarbons (*δ*_C_ 80.4, CH and 84.0, C), respectively, assigned the 15,18-ether linkage of the tetrahydrofuran ring. Moreover, the HMBC correlations observed from H_3_-14 and from both of H_3_-19 and H_3_-20 to oxycarbons (*δ*_C_ 75.0, C and 76.6, CH) positioned the two hydroxy groups at C-13 and C-17, respectively.

**Figure 3:**
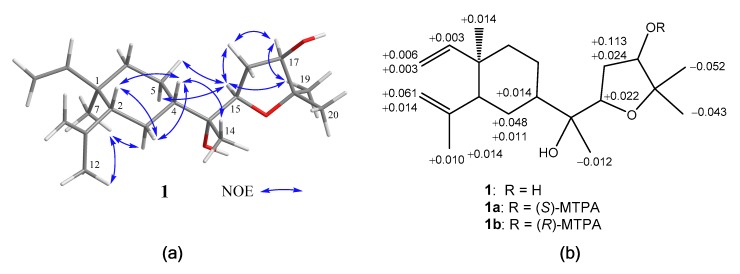
(**a**) Key nuclear Overhauser effect (NOE) correlations of 1; (**b**) ^1^H NMR chemical shift differences ∆(*δ*_S_ − *δ*_R_) in ppm for the MTPA esters of 1.

The relative configurations of **1** at C-1, C-2, C-4, C-13, C-15, and C-17 were assigned by the nuclear Overhauser effect spectroscopy (NOESY) correlation analysis (Figure 3a) and supported by MM2 force-field modeling and NMR data. From the NOE correlations displayed for H_3_-7/H_3_-12 and H-2/H-4 and by comparison of the chemical shifts of C-1 to C-12 with those of the previously reported lobane–type diterpenoids [1,2,5,6,19], the 1R*,2R*,4S* configurations of **1** was determined. Moreover, H_3_-14 and H-15 showed the NOE interactions with the β-oriented H-4 and H_2_-5, leading to the assignment of the corresponding β- and α-orientations of H_3_-14 and H-15, respectively. The α-orientation for the hydroxy group at C-13 was thus suggested and also supported by the down-field shifted H-3 (*δ*_H_ 1.70, m) relative to those of lobane–type diterpenoids having a β-orientated hydroxy group [1]. A conformation analysis for **1** and its 13-epimer, using MM2 calculations and measuring distances between protons showing key NOEs, also revealed the α-orientation of 13-OH, as the distances between both H-15/H_2_-5 and H-4/H_3_-14 were found to be shorter than 2.8 Å in **1**. Furthermore, the NOE correlations between H_3_-19 (*δ*_H_ 1.15, 3H, s) and both H-15 and H-17 disclosed the β-orientations of the hydroxy at C-17. The R*-configurations for C-13, C-15, and C-17 were thus assigned. By using Mosher’s method [20,21], the absolute *R* configuration for C-17 was proven by the analysis of the calculated ∆*δ* (*δ*_S_ − *δ*_R_) values of the neighboring protons to C-17 from the ^1^H NMR data of 17-(*S*)- and 17-(*R*)-α-methoxy-α-(trifluoromethyl)phenylacetyl (MTPA) esters of **1** (**1a** and **1b**), respectively (Figure 3b). Based on the above findings, lobovarol F (**1**) was determined to be (1*R*,2*R*,4*S*,13*R*,15*R*,17*R*)-15,18-epoxyloba-8,10-dien-13,17-diol. To the best of own knowledge, **1** is the first lobane diterpene with a tetrahydrofuran-containing side chain.

**Figure 4:**
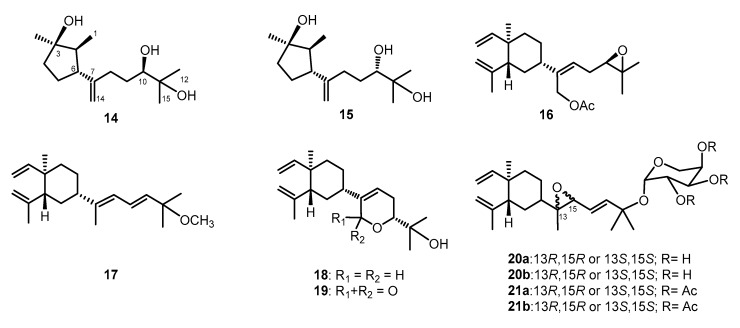
Known sequiterpenoids chokol E (**14**) and 10-*epi*-chokol E (**15**); lobane diterpenoids (**16–19**); fuscosides C and D (**20a** and **20b**), and fuscosides C and D acetates (**21a** and **21b**).

Lobovarol G (**2**) displayed a sodiated ion peak at *m*/*z* 329.2453 in the HRESIMS spectrum, appropriate for a molecular formula C_20_H_34_O_2_. The IR absorption at 3392 cm^−1^ also indicated the presence of hydroxy group. The NMR data of **2** (Table 1) showed resonances and coupling patterns identical to those of the β-elemene ring system in **1** as well as other lobane-type diterpenoids [1,2,3,4,5,6,7]. The fourth degree of unsaturation has arisen from an 1,1-disubstituted double bond (*δ*_C_ 154.5, C and 107.4, CH_2_; and *δ*_H_ 4.82 and 4.77, each 1H, s). NMR data showed that **2** differs with (1*R*,2*R*,4*S*,17*R*)-loba-8,10,13(15)-trien-17,18-diol, isolated from the same organism (*L. varium*) [1] and *L. pauciflorum* [2], only in the position of the double bond of the side chain. The analysis of HMBC correlations further confirmed the C-17 and C-18 positions of the hydroxy groups in the side chain (Figure 2). Compound **2** should possess the same 1*R*,2*R*,4*S*-configuration of the β-elemene ring system as that of **1** by the biogenetic relationship with **1** and the previously reported lobane diterpenoids isolated from genus *Lobophytum* [1,5,19]. Comparison of NMR data of the side chain of **2** (Table 1) with the corresponding data of chokol E (**14**) [22] and 10-*epi*-chokol E (**15**) [23] of known absolute configurations (Figure 4), allowed the elucidation of 17R* configuration in **2**. As **1** and **2** should share the same biosynthetic pathway, therefore, the structure of **2** was suggested to be (1*R*,2*R*,4*S*,17*R*)-loba-8,10,13-trien-17,18-diol.

Lobovarol H (**3**) possessed a molecular formula C_20_H_32_O_2_ (HRESIMS: *m*/*z* 327.2294 [M + Na]^+^ and showed the presence of hydroxy and olefinic functionalities from the IR absorptions at 3352 and 1645 cm^−1^. The NMR data (Table 1) revealed that **3** is a lobane diterpenoid, with the presence of a trisubstituted (*δ*_C_ 145.3, C and 126.1, CH; *δ*_H_ 6.02, d, *J* = 11.0 Hz) and an 1,2-disubstituted double bond (*δ*_C_ 142.0, CH and 121.9, CH; *δ*_H_ 6.61, dd, *J* = 15.0, 11.0 Hz and 5.88, d, *J* = 15.0 Hz); and hydroxymethylene group (*δ*_C_ 60.0 CH_2_; *δ*_H_ 4.31 and 4.29, each 1H, d, *J* = 12.5 Hz) in the side chain.

The NMR data of the side chain of **3** was found to be the same as the corresponding data of known compound **13** except the olefinic methyl of **13** was replaced by a hydroxymethylene group (*δ*_H_ 4.31 and 4.29, each 1H, d, *J* = 12.5 Hz) in **3**. The HMBC correlations of **3** further confirmed the C-13, C-13/C-15, C-16/C-17, and C-18 positions of the hydroxymethylene, two conjugated double bonds, and a hydroxy group, respectively (Figure 2). Finally, the strong NOE correlation of H_2_-14/H-16 and the large *J* value of H-16 and H-17 (15.0 Hz) determined the *Z* and *E* configurations of C-13/C-15 and C-16/C-17 double bonds, respectively. Compound **3** was thus established as (1*R*,2*R*,4*S*,13*Z*,16*E*)-loba-8,10,13(15),16-tetraen-14,18-diol.

Lobovarol I (**4**) displayed a sodiated ion peak [M + Na]^+^ at *m*/*z* 387.2504 in the HRESIMS, appropriate for a molecular formula C_22_H_36_O_4_, and five degrees of unsaturation. The IR absorptions at 3278 and 1740 cm^−1^ revealed the presence of the hydroxy and ester carbonyl groups. The NMR data (Table 1) showed the presence of a trisubstituted double bond (*δ*_C_ 141.0, C and 124.7, CH; *δ*_H_ 5.58, dd, *J* = 8.0, 6.8 Hz), a trisubstituted epoxide (*δ*_C_ 58.6, C; 63.7, CH; *δ*_H_ 2.76, 1H, dd, *J* = 6.4, 6.4 Hz), and an acetoxy group (*δ*_C_ 171.0, C; 21.1 CH_3_; *δ*_H_ 2.06, 3H, s). The remaining two degrees of unsaturation have arisen from the presence of two rings. A decalin bicarbocyclic structure with an angular methyl (*δ*_H_ 0.90, 3H, s) and a methine (*δ*_C_ 54.9, CH; *δ*_H_ 1.27, m) was elucidated from the COSY and HMBC correlations (Figure 2) which showed **4** to be a prenyleudesmane type diterpene [9,10]. The C-4 position of the hydroxy group was confirmed from the HMBC correlation of H_3_-16 (*δ*_H_ 1.12, s,) to C-5 (*δ*_C_ 54.9, CH) and C-4 (*δ*_C_ 72.2, C). Moreover, the NMR data of the side chain of **4** were found to be identical in all aspects to those of the side chain of acetoxylobaoxide (**16**) (Figure 4) [5], the gross structure of **4** was thus established unambiguously.

**Figure 5:**
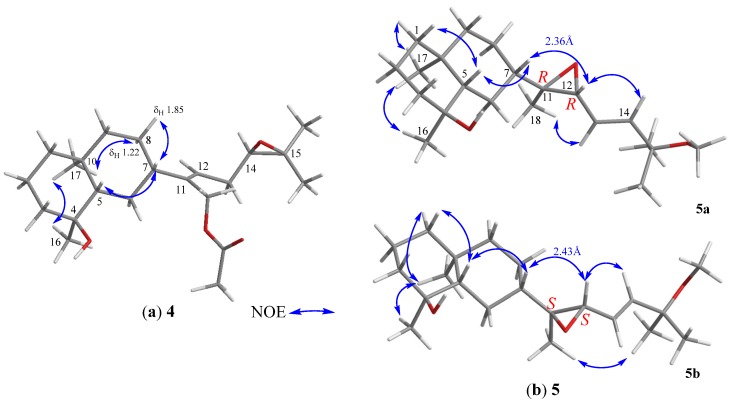
(**a**) Selected NOE correlations of **4**; (**b**) Selected NOE correlations of 11,12-bisepimers.

The relative configuration of **4** was determined based on biogenetic consideration, NOESY correlations (Figure 5a) and NMR data comparison of related metabolites. Accordingly, the steric orientations of H_3_-17, H-5, and H-7 in the bicarbocyclic ring system of prenyleudesmanes **4** and **5**–**11** (*vide infra*) should be α, β, and β, respectively, as the same with the corresponding H_3_-17, H-2, and H-4 of the ring system of the lobanes (**1**–**3**), as the showed by the NOESY analysis of lobovarol I (**4**) (Figure 5a). Consequently, the NOE interaction displayed between H_3_-17 and H_3_-16 indicated the β-orientation of the hydroxy group at C-4. Furthermore, the NMR data of the side chain in **4** were identical to those of the side chain in acetoxylobaoxide (**16**), of which the *R* absolute configuration at C-14 has been determined after reduction of the C-14/C-15 epoxide [5]. From the above results, the structure of **4** was determined as (4*S*,5*S*,7*S*,10*S*,11*Z*,14*R*)-14,15-epoxy-18-acetoxyprenyleudesma-11- en-4-ol.

Lobovarol J (**5a** and **5b**) were obtained as an inseparable mixture (≈ 1:1) of two isomers as revealed from the NMR spectra which displayed two sets of signals with an only slight difference in the chemical shifts for certain carbon and proton atoms. The HRESIMS (*m*/*z* 359.2555 [M + Na]^+^) was determining a molecular formula (C_21_H_36_O_3_) for the mixture of two isomers. Comparison of the NMR data of **5a** and **5b** (Table 2) with those of **4** (Table 1) revealed that lobovarol J possessed the same bicarbocyclic system as that of **4** (Table 2) and the similar side chain of fuscosides C & D triacetates (**21a** and **21b**) [24], except that the sugar moiety in **21a** or **21b** (Figure 4) was replaced by a methoxy group (*δ*_C_ 50.4, CH_3_; *δ*_H_ 3.10, 3H, s) in **5**. The planar structure of **5a** and **5b** was then established, as illustrated in Figure 2. The *E* configuration of the 13,14-double bond and the *trans* 11,12-epoxide were deduced from the *J* values of H-13 and H-14 (16.0 Hz) and the NOE correlations (Figure 5b), respectively. However, by using MM2 calculation for either stable conformation of **5a** or **5b**, it was found that either isomer showed a distance of near 2.4 Å between the NOE-interacting protons H-12 and H-7 (Figure 5b). Therefore, the two new compounds were defined as 11*R*,12*R*- (**5a**) and 11*S*,12*S*- (**5b**) isomers of (4*S*,5*S*,7*S*,10*S*,13*E*)-11,12-epoxy-15-methoxyprenyleudesma-13-en-4-ol with the exact NMR spectroscopic data for each isomer remained for further elucidation.

**Figure 6:**
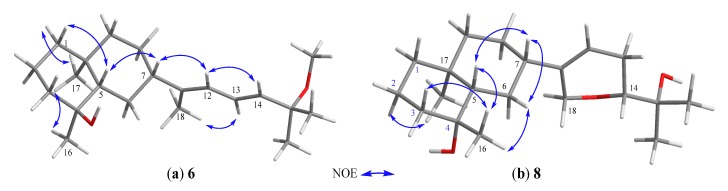
(**a**) Selected NOE correlations of **6**; (**b**) selected NOE correlations of **8**.

Lobovarol K (**6**) has a molecular formula C_21_H_36_O_2,_ as displayed from the sodiated ion peak in the HRESIMS (*m*/*z* 343.2606 [M + Na]^+^), and showed the presence of a hydroxy group (IR 3431 cm^−1^). The NMR spectroscopic data of **6** (Table 2) are similar to a methoxylated (*δ*_C_ 50.4, CH_3_ and *δ*_H_ 3.18, 3H, s) prenyleudesmane, including signals of five methyls. The ^1^H and ^13^C NMR data of **6** further revealed that it is a diterpene possessing the same side chain as that of lobane **17** [2]. The chemical shifts of C-18 (*δ*_C_ 15.3, CH_3_) and *J* value of H-13 and H-14 (15.6 Hz) indicated the *E*-geometry for the 11,12- and 13,14-double bonds, respectively. The NOE correlations (Figure 6a) and the above-mentioned biogenetic consideration established **6** as (4*S*,5*S*,7*S*,10*S*,11*E*,13*E*)-15- methoxyprenyleudesma-11,13- dien-4-ol.

Lobovarol L (**7**) possessed a molecular formula C_20_H_34_O_3_ (HRESIMS *m*/*z* 345.2401 [M + Na]^+^). Compound **7** displayed the combined NMR data of the eudesmane ring system in **4**–**6** (Table 2), and the side chain of lobatriene (**18**) [19]. The planar structure was further established by the analysis of COSY and NMR correlations (Figure 2). The configuration of **7** was established after the structure determination of **8**. NOE correlations between the known α-oriented H_3_-17 and H_3_-16, and β-oriented H-7 with H-5, but not between H-5 and both H_3_-16 and H_3_-17 led to the structure of **7** to be determined as shown in formula **7**.

The HRESIMS of lobovarol M (**8**) showed the same molecular formula C_20_H_34_O_3_ as that of **7**, suggesting **8** to be an isomer for **7**. The NMR data of **8** were found to be very similar to those of **7** (Table 2); however, **8** exhibited more upfield chemical shifts for carbons around C-4, in particular C-2, C-3, and C-5 (∆*δ*_C_ −2.1, −2.1, and −3.1, respectively) relative to the correspondent carbons in **7**, supporting that **8** is the 4-epimer of **7**. Unlike the NOE correlations of **7**, **8** exhibited correlations (Figure 6b) between H_3_-17 and H-2α, but not between H_3_-17 and H_3_-16. Also, H_3_-16 showed NOE interactions with both H-3β and H-5, and H-5 displayed correlation with the β-oriented H-7, thus **8** was determined as 4-epimer of **7**.

Lobovarol N (**9**) displayed a sodiated ion peak at *m*/*z* 359.2191 [M + Na]^+^ in the HRESIMS spectrum of a molecular formula C_20_H_32_O_4_ and IR absorptions at 3430 and 1700 cm^−1^ of the hydroxy and ester carbonyl functionalities. The ^13^C and ^1^H NMR data (Table 3) were similar to those of **7** except the replacement of the 18-hydroxymethylene signals (*δ*_C_ 68.2, CH_2_; *δ*_H_ 4.23 and 4.18, each br d, *J* = 16.4 Hz) in **7** by a signal at *δ*_C_ 164.7 (C) in **9**. Thus, **9** is the 16-oxo derivative of **7**. The NMR data of the side chain of **9** are also nearly identical with those of lobatrienolide (**19**) [5] coexisting in the same organism [1]. A combination of 2D NMR analyses (Figure 2) and biogenetic relationship with the coexisting biogenetically related lobanes **1**,**19** [5], and 17,18-epoxyloba-8,10,13(15)-trien-16-ol [1] with confirmed absolute configurations established the structure of **9** as (4*S*,5*S*,7*S*,10*S*,11*Z*,14*R*)-14,18-epoxyprenyleudesma-11-en-4,15-diol-18-one.

The sodium adduct ion peak [M + Na]^+^ of lobovarol O (**10)** at *m*/*z* 343.2242 in the HRESIMS showed a molecular formula C_20_H_32_O_3_ of **10**. NMR data (Table 3) of **10** showed that it has the same cyclic ether ring as **7**, **8**, and lobatriene (**18**) [19] in the side chain. In comparison of the NMR spectroscopic data of **10** (Table 3) with those of **7** and **8** (Table 2) and analysis of COSY spectrum which showed the proton sequence of H-1 (*δ*_H_ 3.42, dd, *J* = 11.5, 4.0 Hz) to H-3 and the HMBC correlations of H_3_-17 (*δ*_H_ 0.70, 3H, s) to C-1 (*δ*_C_ 79.2), C-5 (*δ*_C_ 47.5), C-9 (*δ*_C_ 36.9), and C-10 (*δ*_C_ 40.2), confirmed the presence of a hydroxy group at C-1 and the prenyleudesmane molecular skeleton of **10**. Furthermore, NOE correlations of H-1 with the β-oriented H-5 and one proton of H_2_-9 (*δ*_H_ 1.20, m) showed the β-orientation of H-1 and this H-9. The other proton at C-9 (*δ*_H_ 1.94, m) was thus assigned as H-9α which exhibited NOE correlation with H_3_-17, but not with both H-1 and H-5, thus H_3_-17 was assigned as α-oriented.

Lobovarol P (**11**) has the molecular formula C_22_H_36_O_4_ (HRESIMS: *m*/*z* 387.2504 [M + Na]^+^). The NMR data (Table 3) revealed that it possesses the same decalin bicyclic structures [10] and the same side-chain [3] of known compounds. The gross structure of **11** was thus determined and the relative configuration of **11** was further established by analysis of the NOESY spectrum which exhibited NOE interactions of the β-oriented H-5 (*δ*_H_ 1.83, d, *J* = 11.5 Hz) with both H-1β (*δ*_H_ 1.28, m) and H-7 (*δ*_H_ 2.10, m), and H-1α (*δ*_H_ 1.28, m) with H_3_-17 (*δ*_H_ 0.73, 3H, s). Thus, H-7 was determined to be positioned on the β face. By the biogenetic consideration, lobovarol P was found to possess (5*R*,7*S*,10*S*)-configuration, the same as that of lobovarol E [1].

Although 5,6-dihydro-2H-pyran and 5,6-dihydro-2H-pyran-2-one moieties have been known to be present in the side chain of lobane diterpenoid isolated from genus *Lobophytum* [1,2,3,5], this is the first report about discovering both pyran and pyranone systems in the structures of prenyleudesmanes. The structures of two known compounds loba-8,10,13(15)-trien-14,17,18-triol-14,17-diacetate (**12**) [3] and the eudesmane derivative (**13**) [9] were established mainly based on the MS and partial ^1^H NMR data. The detailed 1D and 2D NMR spectroscopic analysis enabled us to fully assign the ^1^H and ^13^C NMR data of these two diterpenoids for the first time.

Cytotoxicity for compounds **1**, **2**, **4**, **7**, **8**, **12**, and **13** against the growth of human colon adenocarcinoma (DLD-1), human colon carcinoma (HT-29), and human liver bile duct carcinoma (HuCCT-1); and for **3**, **5**, **6**, and **9**–**11** against DLD-1 and mouse lymphocytic leukemia (P388) cancer cell lines using the Alamar Blue assay has been screened [25,26]. The results showed that all of the tested metabolites did not exhibit cytotoxicity towards the above cell lines (IC_50_ > 40 μg/mL) compared to doxorubicin HCl (IC_50_ 0.9~7.4 μg/mL).

The anti-inflammatory activities of diterpenoids **2**–**13** against the fMLF/CB-induced pro-inflammatory responses on human neutrophils were evaluated [27,28], too. The results (Table 4) showed that **12** and **13** have potent inhibitory effect against the elastase release (IC_50_ 6.9 ±2.7 and 4.4 ± 0.7 μM, respectively). Compounds **2** and **4** could suppress elastase release (IC_50_ 18.8 ± 1.8 and 20.00 ± 3.0 μM), too, while **13** is the only compound also could significantly suppress the superoxide anion generation (IC_50_ 13.7 ± 4.4 μM). Thus **2**, **4**, **12**, and **13**, in particular **12** and **13**, have the potential to become anti-inflammatory agents. Compound **1** was found to strongly suppress elastase release in the absence of fMLF/CB, and is worthy for further biological study.

Although the structures of lobanes **1**–**3**, and **12** and eudesmanes **4**–**11**, and **13**, along with the previously reported related metabolites from the same organism [1] possess different ring structures, they are coexisted in *L. varium* and are biogenetically related to each other

**Scheme 1:**
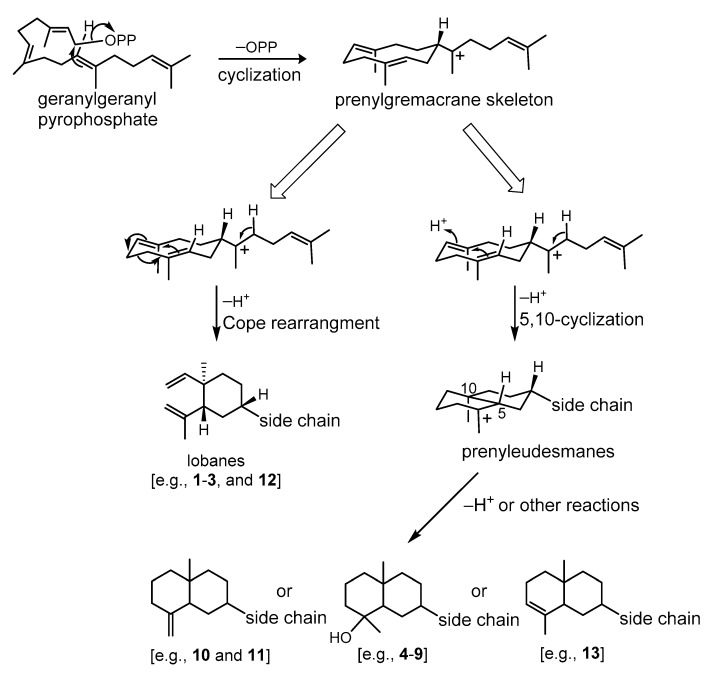
Plausible biosynthetic pathway of lobanes and prenyleudesmane-type diterpenoids.

A prenylgermacrene has been regarded as the same biosynthetic precursor [8,9] and we further propose a stereospecific biosynthesis for lobane and prenyleudesmane diterpenoids through Cope rearrangement and acid-catalyzed cyclization, respectively [9,29], as illustrated in Scheme 1. Thus, the co-occurrence of lobanes and prenyleudesmanes could be found in the same soft coral as in the cases of *L. varium* [1], *Lobophytum* sp. [9], *Eunicea fusca* [24], *Sinularia gyrosa* [30], and *Sinularia polydactyla* [31], is scientifically reasonable.

## 3. Experimental Section

### 3.1. General Experimental Procedures

Optical rotations and IR spectra were measured on a JASCO P-1020 polarimeter and FT/IR-4100 infrared spectrophotometer (Jasco Corporation, Tokyo, Japan), respectively. ESIMS and HRESIMS experiments were performed on VG Quattro GC/MS and Bruker APEX II mass spectrometers, respectively. The NMR spectra were recorded on a Varian Unity Inova 500 FT-NMR (Varian Inc., Palo Alto, CA, USA) at 500 and 125 MHz for ^1^H and ^13^C, respectively; or on a Varian 400 FT-NMR at 400 and 100 MHz for ^1^H and ^13^C, respectively. Silica gel 60 or reversed-phase silica gel (RP-18; 230–400 mesh) and precoated silica gel plates (Kieselgel 60 F254, 0.2 mm) (Merck, Darmstadt, Germany) were used for open column chromatography (CC) and analytical TLC analysis, respectively. Isolation and purification of isolates by HPLC were performed by Hitachi L-2455 instrument equipped with an RP-18 column (ODS-3, 5 µm, 250 × 20 mm; Sciences Inc., Tokyo, Japan).

### 3.2. Aminal Material

The soft coral *L. varium* (Tixier-Durivault, 1970) was collected in March 2013 at a depth of 10 to 15 m at Jihui Fish Port, Taitung, Taiwan (23°7′2″ N, 121°23′49.2″ E) and identified by Professor Chang-Feng Dai, Institute of Oceanography, National Taiwan University, Taipei, Taiwan [1].

### 3.3. Extraction and Isolation

The sliced frozen soft coral *L. varium* (1.3 kg, wet weight) was exhaustively extracted with EtOAc, and the resulting solvent-free extract (55.40 g) was fractionated by silica gel column chromatography into 24 fractions (F1 to F24) as described before^1^. F19 (1.39 g), eluting with 50% EtOAc in *n*-hexane, was permeated through a column of Sephadex LH-20 using acetone to afford two diterpenoid-rich fractions F19-1 (105.0 mg) and F19-2 (94.0 mg). F19-1 was chromatographed over silica gel and eluted with EtOAc*–n*-hexane (1:3) to give two subfractions F19-1a (70.5 mg) and F19-1b (15.0 mg). F19-1a was further divided by reverse phase (RP-18) column chromatography, using MeOH–H_2_O, into two subfractions F19-1a1 and F19-1a2 which were purified separately by RP-18 HPLC, using CH_3_CN–H_2_O (2:1), to yield **1** (2.4 mg) and **2** (3.1 mg) from F19-1a1, and **12** (10.0 mg) from F19-1a2, respectively. F19-1b (15.0 mg) was purified by RP-18 HPLC using CH_3_CN–H_2_O (1.5:1) to afford **4** (1.1 mg). F19-2 was also primarily chromatographed over silica gel column using EtOAc–*n*-hexane (1:3) where the eluted diterpenoid-rich fraction (50.1 mg) was further isolated by RP-18 column chromatography, using MeOH–H_2_O (9:1 then 2:1), to yield **13** (1.4 mg), **7** (1.5 mg), and **8** (4.8 mg), respectively. F20 (1.20 g), eluted with 66.7% EtOAc in *n*-hexane, was fractionated over silica gel column, using EtOAc–*n*-hexane (5:95 to 0:100, gradient), to yield four fractions (F20-1 to F20-4). Isolation of F20-1 over RP-18 silica gel column, using MeOH–H_2_O (9:1), gave **5** (2.2 mg) and **6** (3.8 mg), respectively. F20-2 was further separated by RP-HPLC, using MeOH–H_2_O (6:1), to afford **10** (2.0 mg) and **11** (3.2 mg), respectively. F21 (0.50 g), eluted with 100% EtOAc, was similarly further fractionated, as for fractionation of F20, to give F21-1 and F21-2. RP-18 silica gel column chromatography was then applied to purify F21-1, using MeOH–H_2_O (5:1), to afford **3** (5.0 mg) and to further purify F21-2, using MeOH–H_2_O (6:1), to yield **9** (4.0 mg).

Lobovarol F (**1**): Colorless oil; ${\left[\alpha \right]}_{D}^{24}$ + 2.8 (*c* 2.4, CHCl_3_); IR (neat) *v*_max_ 3393, 2926, 1643, 1532, 1462, 1147, 1024, and 897 cm^−1^; ^13^C and ^1^H NMR data (see Table 1); ESIMS *m*/*z* 345 [M + Na]^+^; HRESIMS *m*/*z* 345.2403 [M + Na]^+^ (calcd for C_20_H_34_O_3_Na, 345.2400).

Lobovarol G (**2**): Colorless oil; ${\left[\alpha \right]}_{D}^{24}$ + 44.5 (*c* 3.1, CHCl_3_); IR (neat) *v*_max_ 3392, 2926, 2860, 1642, 1523, and 891 cm^−1^; ^13^C and ^1^H NMR data (see Table 1); ESIMS *m*/*z* 329 [M + Na]^+^; HRESIMS *m*/*z* 329.2453 [M + Na]^+^ (calcd for C_20_H_34_O_2_Na, 329.2451).

Lobovarol H (**3**): Colorless oil; ${\left[\alpha \right]}_{D}^{24}$ −46.7 (*c* 0.57, CHCl_3_); IR (neat) *v*_max_ 3352, 2926, 1645, 1456, 1374, and 898 cm^−1^; ^13^C and ^1^H NMR data (see Table 1); ESIMS *m*/*z* 327 [M + Na]^+^; HRESIMS *m*/*z* 327.2294 [M + Na]^+^ (calcd for C_20_H_32_O_2_Na, 327.2295).

Lobovarol I (**4**): Colorless oil; ${\left[\alpha \right]}_{D}^{24}$ + 66.0 (*c* 1.1, CHCl_3_); IR (neat) *v*_max_ 3278, 2919, 2852, 1740, and 1531 cm^−1^; ^13^C and ^1^H NMR data (see Table 1); ESIMS *m*/*z* 387 [M + Na]^+^; HRESIMS *m*/*z* 387.2504 [M + Na]^+^ (calcd for C_22_H_36_O_4_Na, 387.2506).

Lobovarol J (mixture of **5a** and **5b**): Colorless oil; ${\left[\alpha \right]}_{D}^{24}$ + 14.2 (*c* 0.63, CHCl_3_); IR (neat) *v*_max_ 3443, 2929, 1531, 1456, 1378, and 1068 cm^−1^; ^13^C and ^1^H NMR data (see Table 2); ESIMS *m*/*z* 359 [M + Na]^+^; HRESIMS *m*/*z* 359.2555 [M + Na]^+^ (calcd for C_21_H_36_O_3_Na, 359.2557).

Lobovarol K (**6**): Colorless oil; ${\left[\alpha \right]}_{D}^{24}$ −10.9 (*c* 0.46, CHCl_3_); IR (neat) *v*_max_ 3431, 2922, 1670, 1535, 1458, 1377, and 1071 cm^−1^; ^13^C and ^1^H NMR data (see Table 2); ESIMS *m*/*z* 343 [M + Na]^+^; HRESIMS *m*/*z* 343.2606 [M + Na]^+^ (calcd for C_21_H_36_O_2_Na, 343.2608).

Lobovarol L (**7**): Colorless oil; ${\left[\alpha \right]}_{D}^{24}$ + 99.2 (*c* 1.5, CHCl_3_); IR (neat) *v*_max_ 3378, 2922, 2852, 1540, 1458, 1089, 906, and 670 cm^−1^; ^13^C and ^1^H NMR data (see Table 2); ESIMS *m*/*z* 345 [M + Na]^+^; HRESIMS *m*/*z* 345.2401 [M + Na]^+^ (calcd for C_20_H_34_O_3_Na, 345.2400).

Lobovarol M (**8**): Colorless oil; ${\left[\alpha \right]}_{D}^{24}$ + 87.7 (*c* 4.8, CHCl_3_); IR (neat) *v*_max_ 3459, 2927, 2852, 1451, 1378, 1161, 1088, and 756 cm^−1^; ^13^C and ^1^H NMR data (see Table 2); ESIMS *m*/*z* 345 [M + Na]^+^; HRESIMS *m*/*z* 345.2401 [M + Na]^+^ (calcd for C_20_H_34_O_3_Na, 345.2400).

Lobovarol N (**9**): Colorless oil; ${\left[\alpha \right]}_{D}^{24}$ + 59.7 (*c* 0.54, CHCl_3_); IR (neat) *v*_max_ 3430, 2924, 1700, 1429, 1376, and 1096 cm^−1^; ^13^C and ^1^H NMR data (see Table 3); ESIMS *m*/*z* 359 [M + Na]^+^; HRESIMS *m*/*z* 359.2191 [M + Na]^+^ (calcd for C_20_H_32_O_4_Na, 359.2193).

Lobovarol O (**10**): Colorless oil; ${\left[\alpha \right]}_{D}^{24}$ + 48.1 (*c* 0.57, CHCl_3_); IR (neat) *v*_max_ 3433, 2926, 1696, 1532, 1024, and 670 cm^−1^; ^13^C and ^1^H NMR data (see Table 3); ESIMS *m*/*z* 343 [M + Na]^+^; HRESIMS *m*/*z* 343.2242 [M + Na]^+^ (calcd for C_20_H_32_O_3_Na, 343.2244).

Lobovarol P (**11**): Colorless oil; ${\left[\alpha \right]}_{D}^{24}$ −54.0 (*c* 0.71, CHCl_3_); IR (neat) *v*_max_ 3503, 2925, 1731, 1373, and 1237 cm^−1^; ^13^C and ^1^H NMR data (see Table 3); ESIMS *m*/*z* 387 [M + Na]^+^; HRESIMS *m*/*z* 387.2504 [M + Na]^+^ (calcd for C_2__2_H_3__6_O_4_Na, 387.2506).

Compound **12**: Colorless oil; ${\left[\alpha \right]}_{D}^{24}$ + 57.0 (*c* 14.3, CHCl_3_); IR (neat) *v*_max_ 3496, 2927, 2937, 1736, 1640, 1442, 1373, and 1027 cm^−1^; ^1^H NMR (CDCl_3_, 400 MHz): *δ*_H_ 5.80 (1H, dd, *J* = 17.6, 10.4 Hz, H-8), 5.48 (1H, dd, *J* = 7.2, 7.2 Hz, H-15), 4.89 (1H, d, *J* = 17.6 Hz, H-9a), 4.89 (1H, d, *J* = 10.4 Hz, H-9b), 4.85 (1H, dd, *J* = 9.2, 4.0 Hz, H-17), 4.81 (1H, s, H-11a), 4.66 (1H, d, *J* = 12.0 Hz, H-14a), 4.58 (1H, d, *J* = 12.0 Hz, H-14b), 4.56 (1H, s, H-11b), 2.46, (2H, m, H-6, H_2_-16), 2.06 (3H, s, 17-OCOCH_3_), 2.05 (3H, s, 14-OCOCH_3_) 2.03, (1H, m, H-4), 1.99 (1H, dd, *J* = 11.6, 3.6 Hz, H-2), 1.69 (3H, s, H_3_-12), 1.53, (2H, m, H_2_-5), 1.49 (2H, m, H_2_-3), 1.41 (2H, m, H_2_-6), 1.22 (6H, s, H_3_-19 and H_3_-20), 0.98 (3H, s, H_3_-7); ^13^C NMR (CDCl_3_, 100 MHz): *δ*_C_ 171.0 (C, 14-OCOCH_3_), 170.6 (C, 17-OCOCH_3_), 150.0 (CH, C-8), 147.4 (C, C-10), 140.7 (C, C-13), 125.6 (CH, C-15), 112.2 (CH_2_, C-11), 110.0 (CH_2_, C-9), 78.9 (CH, C-17), 72.1 (C, C-18), 61.1 (CH_2_, C-14), 52.6 (CH, C-2), 43.7 (CH, C-4), 39.8 (CH_2_, C-6), 39.7 (C, C-1), 33.1 (CH_2_, C-3), 28.2 (CH_2_, C-16), 27.1 (CH_2_, C-5), 26.5 (CH_3_, C-19), 25.3 (CH_3_, C-20), 24.8 (CH_3_, C-12), 21.1 (CH_3_, 14-OCOCH_3_), 20.9 (CH_3_, 17-OCOCH_3_), 16.5 (CH_3_, C-7); ESIMS *m*/*z* 429 [M + Na]^+^; HRESIMS *m*/*z* 429.2609 [M + Na]^+^ (calcd for C_24_H_38_O_5_Na, 429.2612).

Compound **13**: Colorless oil; ${\left[\alpha \right]}_{D}^{24}$ − 81.0 (*c* 1.4, CHCl_3_); IR (neat) *v*_max_ 3254, 2925, 2857, 1640, 1450, and 1023 cm^−1^; ^1^H NMR (CDCl_3_, 500 MHz): *δ*_H_ 6.50 (1H, dd, *J* = 15.5, 11.0 Hz, H-13), 5.90 (1H, d, *J* = 11.0 Hz, H-12), 5.78 (1H, d, *J* = 15.5 Hz, H-14), 5.29 (1H, s, H-3), 4.58 (1H, dd, *J* = 7.0, 7.0 Hz, H-1), 3.55 (1H, dd, *J* = 7.0, 7.0 Hz, H-1), 2.31 (1H, m, H-2a), 1.98 (2H, m, H-2b and H-7), 1.95 (1H, m, H-5), 1.92 (1H, m, H-9a), 1.71 (1H, m, H-6a), 1.80 (3H, s, H_3_-18), 1.60 (2H, m, H_2_-8), 1.58 (3H, s, H_3_-16), 1.36 (6H, s, H_3_-19 and H_3_-20), 1.29 (1H, m, H-6b), 1.14 (1H, m, H-9b), 0.79 (3H, s, H_3_-17); ^13^C NMR (CDCl_3_, 125 MHz): *δ*_C_ 143.6 (C, C-11), 139.3 (CH, C-14), 135.2 (C, C-4), 123.0 (CH_2_, C-13), 122.7 (CH, C-12), 119.5 (CH, C-3), 76.4 (CH, C-1), 71.0 (C, C-15), 52.8 (CH_2_, C-2), 48.0 (CH, C-7), 46.6 (CH, C-5), 37.4 (C, C-10), 35.0 (CH_2_, C-9), 29.9 (2 x CH_3_, C-19 and C-20), 28.2 (CH_2_, C-6), 26.0 (CH_2_, C-8), 20.8 (CH_3_, C-16), 15.0 (CH_3_, C-18), 9.5 (CH_3_, C-17); ESIMS *m*/*z* 327 [M + Na]^+^; HRESIMS *m*/*z* 327.2296 [M + Na]^+^ (calcd for C_20_H_32_O_2_Na, 327.2295).

#### Preparation of (*S*)- and (*R*)-MTPA Esters of 1

To a solution of **1** (1.0 mg, 3.1 μmol) in anhydrous pyridine (200 μL) was added *S*-(+)- -α-methoxy-α-(trifluoromethyl)phenylacetyl (MTPA) chloride (20 μL), and the mixture was further reacted for 20 h at room temperature. The reaction mixture was then processed as previously described [20,21] to yield the (*R*)-MTPA ester **1a** (0.5 mg, 0.93 μmol, 30%). The correspondent (*S*)-MTPA ester **1b** was similarly yielded from the reaction of *R*-(–)-MTPA chloride with **1**. ^1^H NMR (CDCl_3_, 400 MHz) of **1a**: *δ*_H_ 5.8005 (1H, dd, *J* = 17.6, 10.4 Hz, H-8), 4.892 (1H, d, *J* = 12 Hz, H-9b), 5.114 (1H, dd, *J* = 5.6, 5.6 Hz, H-17), 4.8895 (1H, d, *J* = 16.4 Hz, H-9a), 4.822 (1H, s, H-11b), 4.591 (1H, s, H-11a), 3.949 (1H, dd, *J* = 8.4, 6.8 Hz, H-15), 2.395 (1H, ddd, *J* = 10.0, 6.8, 6.8 Hz, H-16b), 1.994 (1H, m, H-16a), 1.9475 (1H, m, H-2), 1.708 (3H,s, H_3_-12), 1.689 (1H, d, *J* = 15.2, H-3b), 1.522 (1H, m, H-3a), 1.367 (1H, m, H-4), 1.394-1.229 (4H, m, H_2_-5 and H_2_-6), 1.279 (3H, s, H_3_-20), 1.210 (3H, s, H_3_-14), 1.158 (3H, s, H_3_-19), 0.981 (3H, s, H_3_-7); ^1^H NMR (CDCl_3_, 400 MHz) of **1b**: *δ*_H_ 5.803 (1H, dd, *J* = 17.6, 10.4 Hz, H-8), 4.895 (1H, d, *J* = 12 Hz, H-9b), 5.136 (1H, dd, *J* = 6.0, 6.0 Hz, H-17), 4.8945 (1H, d, *J* = 16.4 Hz, H-9a), 4.883 (1H, s, H-11b), 4.605 (1H, s, H-11a), 3.971 (1H, dd, *J* = 8.0, 7.6 Hz, H-15), 2.419 (1H, ddd, *J* = 9.6, 6.8, 6.8 Hz, H-16b), 2.107 (1H, m, H-16a), 1.989 (1H, m, H-2), 1.718 (3H,s, H_3_-12), 1.7665 (1H, br d, *J* = 14.0, H-3b), 1.5325 (1H, m, H-3a), 1.415 (1H, m, H-4), 1.394-1.256 (4H, m, H_2_-5 and H_2_-6), 1.236 (3H, s, H_3_-20), 1.198 (3H, s, H_3_-14), 1.106 (3H, s, H_3_-19), 0.995 (3H, s, H_3_-7).

### 3.4. Cyotoxic Testing

Cytotoxicities of **1**–**11** were assayed using Almar Blue assay [25,26]. Doxorubicin HCl, employed as positive control, displayed cytotoxic activity toward DLD-1, HT-29, HuCCT-1, and P388 cell lines with IC_50_ 0.9, 4.4, 2.6, and 7.4 μg/mL, respectively.

### 3.5. In Vitro Anti-Inflammatory Testing

The experiment for measuring superoxide anion generation and elastase release were manipulated according to previously reported method [28,32,33].

## 4. Conclusions

In conclusion, our further chemical investigation and biological evaluation on the EtOAc extract of the soft coral *L. varium* disclosed three new lobane and eight prenyleudesmane diterpenoids along with two known derivatives. Compound **1** is the first labane possessing a tetrahydrofuran ring at the end of the side chain, and displayed ability to inhibit elastase release in the absence of fMLF/CB. Compounds **2**, **4**, **12**, and **13**, in particular **12** and **13**, displayed the potential to be the leads for anti-inflammatory medicines. Our proposed stereospecific biosynthetic pathway can explain the common coexistence of both lobanes and prenyleudesmanes in the soft coral *L. varium*.

## Figures and Tables

**Figure 1:**
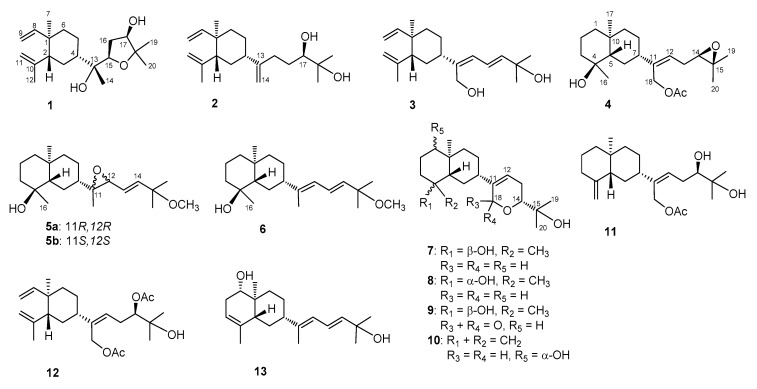
Structures of compounds **1**–**13**.

**Table 1:** ^13^C and ^1^H NMR spectroscopic data of compounds **1**–**4**.

Position	1		2		3		4	
	*δ*_C_*^a^* (Mult.)*^b^*	*δ*_H_*^c^* (*J* in Hz)	*δ*_C_*^a^* (Mult.)	*δ*_H_*^c^* (*J* in Hz)	*δ*_C_*^d^* (Mult.)	*δ*_H_*^e^* (*J* in Hz)	*δ*_C_*^a^* (Mult.)	*δ*_H_*^c^* (*J* in Hz)
1	39.9, C		39.8, C		39.7, C		41.0, CH_2_	1.38 m; 1.08 m
2	52.4, CH	1.98 m	52.8, CH	2.01 m	52.8, CH	2.04 dd (7.5, 7.5)	20.1, CH_2_	1.55–1.15 m
3	27.4, CH_2_	1.70 m; 1.50 m	33.3, CH_2_	1.58 m	33.4, CH_2_	1.60 m	43.4, CH_2_	1.80 d (13.2); 1.40 m
4	45.1, CH	1.52 m	44.4, CH	1.93 m	43.6, CH	2.20 m	72.2, C	
5	23.1, CH_2_	1.35 m; 1.24 m	27.4, CH_2_	1.66 m; 1.47 m	27.2, CH_2_	1.64 m; 1.52 m	54.9, CH	1.27 m
6	39.7, CH_2_	1.42 m	39.7, CH_2_	1.52 m; 1.47 m	39.9, CH_2_	1.50 m	27.2, CH_2_	1.52 m
7	16.6, CH_3_	0.99 s	16.6, CH_3_	1.01 s	16.6, CH_3_	1.02 s	44.7, CH	2.06 m
8	150.0, CH	5.80 dd (18.0, 10.4)	150.2, CH	5.82 dd (17.2, 10.8)	150.1, CH	5.82 dd (17.5, 11.5)	26.4, CH_2_	1.85 d (12.0); 1.22 m
9	110.0, CH_2_	4.90 d (10.4)	110.0, CH_2_	4.90 d (17.2)	110.0, CH_2_	4.92 d (11.5)	44.6, CH_2_	1.45 m
		4.89 br d (18.0)		4.90 d (10.8)		4.89 d (17.5)		1.21 m
10	147.6, C		147.6, C		147.5, C		34.6, C	
11	112.1, CH_2_	4.83 s; 4.59 s	112.1, CH_2_	4.59 s; 4.82 br s	112.2, CH_2_	4.82 s; 4.59 s	141.0, C	
12	24.9, CH_3_	1.71 s	24.8, CH_3_	1.71 s	24.8, CH_3_	1.71 s	124.7, CH	5.58 dd (8.0, 6.8)
13	75.0, C		154.5, C		145.3, C		27.9, CH_2_	2.39–2.30 m
14	20.4, CH_3_	1.26 s	107.4, CH_2_	4.82 s	60.0 CH_2_	4.31 d (12.5)	63.7 CH	2.76 dd (6.4, 6.4)
				4.77 s		4.29 d (12.5)		
15	80.4, CH	4.01 dd (9.2, 4.0)	32.0, CH_2_	2.35 m; 2.12 m	126.1, CH	6.02 d (11.0)	58.6, C	
16	34.3, CH_2_	2.39 ddd (14.4, 9.2, 6.0)	30.0, CH_2_	1.64 m	121.9, CH	6.61 dd (15.0, 11.0)	22.7, CH_3_	1.12 s
		1.95 dd (14.4, 4.0)		1.47 m				
17	76.6, CH	3.78 br d (6.0)	78.4, CH	3.78 d (10.8)	142.0, CH	5.88 d (15.0)	18.7, CH_3_	0.90 s
18	84.0, C		73.1, C		70.9, C		61.2, CH_2_	4.62 d (12.0); 4.61 d (12.0)
19	25.5, CH_3_	1.15 s	23.3, CH_3_	1.18 s	29.8, CH_3_	1.36 s	24.8, CH_3_	1.33 s
20	22.2, CH_3_	1.30 s	26.5, CH_3_	1.23 s	29.8, CH_3_	1.36 s	18.7, CH_3_	1.31 s
Ac							171.0, C	
							21.1, CH_3_	2.06 s

*^a^* Spectrum recorded at 100 MHz in CDCl_3_. *^b^* Attached protons were deduced by the distortionless enhancement by polarization transfer (DEPT) experiment. *^c^* Spectrum recorded at 400 MHz in CDCl_3_. *^d^* Spectrum recorded at 125 MHz in CDCl_3_. *^e^* Spectrum recorded at 500 MHz in CDCl_3_.

**Table 2:** ^13^C and ^1^H NMR spectroscopic data of compounds **5**–**8**.

Position	5a + 5b		6		7		8	
	*δ*_C_*^a^* (Mult.) *^b^*	*δ*_H_*^c^* (*J* in Hz)	*δ*_C_*^d^* (Mult.)	*δ*_H_*^e^* (*J* in Hz)	*δ*_C_*^d^* (Mult.)	*δ*_H_*^e^* (*J* in Hz)	*δ*_C_*^d^* (Mult.)	*δ*_H_*^e^* (*J* in Hz)
1	42.1, CH_2_	1.37 m; 1.06 m	41.0, CH_2_	1.42 m; 1.11 m	41.0, CH_2_	1.40 m; 1.07 m	41.5, CH_2_	1.44 m; 1.08 m
2	20.9, CH_2_	1.53 m; 1.49 m	20.1, CH_2_	1.58 m; 1.55 m	20.1, CH_2_	1.58-1.53 m	18.0, CH_2_	1.85 m;1.65 m
3	44.3, CH_2_	1.71 m	43.3, CH_2_	1.82 m; 1.38 m	43.4, CH_2_	1.78 br d (12.0); 1.38 m	41.3, CH_2_	1.69 m; 1.44 m
4	71.4, C		72.2, C		72.2, C		71.9, C	
5	55.2/55.3, CH	1.21 m	54.9, CH	1.25 m	54.9, CH	1.25 m	51.8, CH	1.07 m
6	23.8/23.9, CH_2_	1.54 m	26.5, CH_2_	1.53 m; 1.51 m	26.0, CH_2_	1.86 br d (9.2); 1.14 m	25.7, CH_2_	1.70 m; 1.34 m
7	48.2/48.3, CH	1.20 m	48.3, CH	2.01 m	42.5, CH	1.86 br d (9.2)	42.9, CH	1.85 m
8	23.0, CH_2_	2.00 m	25.8, CH_2_	1.81 m; 1.26 m	27.0, CH_2_	1.48 m; 1.42 m	27.3, CH_2_	1.48 m
9	45.2, CH_2_	1.41 m; 1.16 m	44.6, CH_2_	1.47 m; 1.22 m	44.6, CH_2_	1.42 m; 1.19 m	43.8, CH_2_	1.36 m; 1.14 m
10	35.4, C		34.6, C		34.6, C		33.7, C	
11	65.7, C		143.4, C		141.4, C		141.5, C	
12	62.6/62.8, CH	3.24/3.26 d (7.0)	122.5, CH	5.90 d (10.8)	116.3, CH	5.57 br d (5.6)	116.4, CH	5.59 br d (4.0)
13	126.3/126.4, CH	5.557/5.563 dd (16.0, 7.0)	126.0, CH	6.39 dd (15.6, 10.8)	25.3, CH_2_	2.14 br d (13.6, 12.8)	25.3, CH_2_	2.16 m
						1.97 m		1.98 m
14	141.7/141.8, CH	5.84 d (16.0)	136.6 CH	5.57 d (15.6)	80.3 CH	3.26 dd (11.2, 3.6)	80.3 CH	3.27 dd (10.8, 3.2)
15	75.1, C		75.1, C		71.1, C		71.7, C	
16	23.1, CH_3_	1.07 s	22.7, CH_3_	1.12 s	22.7, CH_3_	1.11 s	30.3, CH_3_	1.15 s
17	19.1, CH_3_	0.90 s	18.7, CH_3_	0.90 s	18.6 CH_3_	0.88 s	18.6, CH3	1.04 s
18	14.3/14.4, CH_3_	1.23 s	15.3, CH_3_	1.80 s	68.2, CH_2_	4.18 d (16.4)	68.2, CH2	4.19 d (16.4)
						4.23 d (16.4)		4.21 d (16.4)
19	26.0/26.1, CH_3_	1.24 s	26.0, CH_3_	1.30 s	26.1, CH_3_	1.21 s	26.1, CH_3_	1.21 s
20	26.3/26.4, CH_3_	1.24 s	26.0, CH_3_	1.30 s	23.6, CH_3_	1.17 s	23.6, CH_3_	1.17 s
OCH_3_	50.4, CH_3_	3.10 s	50.4, CH_3_	3.18 s				

*^a^* Spectrum recorded at 125 MHz in acetone-d_6_. *^b^* Attached protons were deduced by the DEPT experiment. *^c^* Spectrum recorded at 500 MHz in acetone-d_6_. *^d^* Spectrum recorded at 100 MHz in CDCl_3_. *^e^* Spectrum recorded at 400 MHz in C.

**Table 3:** ^13^C and ^1^H NMR spectroscopic data of compounds **9**–**11**.

Position	9		10		11	
	*δ*_C_*^a^* (Mult.)*^b^*	*δ*_H_*^c^* (*J* in Hz)	*δ*_C_*^d^* (Mult.)	*δ*_H_*^e^* (*J* in Hz)	*δ*_C_*^d^* (Mult.)	*δ*_H_*^e^* (*J* in Hz)
1	40.9, CH_2_	1.40 m; 1.10 m	79.2, CH	3.42 dd (11.5, 4.0)	41.8, CH_2_	1.44 m; 1.28 m
2	20.1, CH_2_	1.56 m	31.4, CH_2_	1.82 m;1.56 m	23.4, CH_2_	1.63 m
3	43.6, CH_2_	1.83 m; 1.80 m	34.1, CH_2_	2.32 m; 2.11 m	36.8, CH_2_	2.31 m; 2.01 m
4	72.1, C		148.5, C		150.7, C	
5	54.9, CH	1.32 m	47.5, CH	1.77 m	49.9, CH	1.83 d (11.5)
6	25.4, CH_2_	1.90 m	28.8, CH_2_	1.64 m; 1.31 m	29.7, CH_2_	1. 56 m; 1.26 m
7	39.5, CH	2.57 m	41.2, CH	1.83 m	44.0, CH	2.01 m
8	27.9, CH_2_	1.67 m; 1.30 m	26.7, CH_2_	1.59 m; 1.42 m	27.5, CH_2_	1.60 m; 1.42 m
9	44.6, CH_2_	1.43 m; 1.34 m	36.9, CH_2_	1.94 m; 1.20 m	41.2, CH_2_	1.51 m; 1.42 m
10	34.6, C		40.2, C		35.9, C	
11	137.2, C		141.2, C		141.6, C	
12	136.7, CH	6.63 d (5.6)	116.4, CH	5.58 d (5.5)	126.6, CH	5.61 t (7.5)
13	24.5, CH_2_	2.49 m; 2.36 m	25.3, CH_2_	2.17 m; 1.96 m	30.4, CH_2_	2.32 m
14	83.3 CH	4.13 dd (12.8, 3.6)	80.3 CH	3.28 dd (11.0, 3.5)	77.6 CH	3.42 m
15	71.0, C		71.7, C		72.7, C	
16	22.5, CH_3_	1.12 s	107.0, CH_2_	4.76 s; 4.50 s	105.5, CH_2_	4.71 s; 4.42 s
17	18.7, CH_3_	0.91 s	10.2 CH_3_	0.70 s	16.4, CH_3_	0.73 s
18	164.7, C		68.2, CH_2_	4.20 br s	61.6, CH_2_	4.54 d (12.0)
						4.76 d (12.0)
19	24.4, CH_3_	1.25 s	23.7, CH_3_	1.17 s	23.8, CH_3_	1.20 s
20	25.9, CH_3_	1.33 s	26.1, CH_3_	1.22 s	26.3, CH_3_	1.24 s
Ac					171.3, C	
					21.1, CH_3_	2.06 s

*^a^* Spectrum recorded at 100 MHz in CDCl_3_. *^b^* Attached protons were deduced by the DEPT experiment. *^c^* Spectrum recorded at 400 MHz in CDCl_3_. *^d^* Spectrum recorded at 125 MHz in CDCl_3_. *^e^* Spectrum recorded at 500 MHz in CDCl_3_.

**Table 4:** Inhibitory effects of compounds **1**–**13** on the generation of superoxide anion and release of elastase in fMLF/CB-stimulated human neutrophils.

Compound	Superoxide Anion			Elastase Release		
	IC_50_ (μM)	Inh % *^a^*		IC_50_ (μM)	Inh %	
**1**	>20	4.6 ± 3.8 *^b^*		>20	64.3 ± 3.7 *^b^*	
**2**	>20	18.1 ± 4.0	*	18.8 ± 1.8	87.3 ± 7.9	***
**3**	>20	4.3 ± 4.2		>20	3.8 ± 2.6	
**4**	>20	40.2 ± 7.3	**	20.0 ± 3.0	53.2 ± 0.2	***
**5**	>20	8.4 ± 7.4		>20	3.5 ± 1.4	
**6**	>20	4.2 ± 4.4		>20	−10.9 ± 5.1	
**7**	>20	22.9 ± 7.6	*	NT *^c^*	NT	
**8**	>20	19.1 ± 6.9	*	>20	37.8 ± 6.2	***
**9**	>20	5.3 ± 3.2		>20	5.5 ± 0.6	***
**10**	>20	2.7 ± 5.0		>20	5.3 ± 1.6	*
**11**	>20	8.9 ± 7.0		>20	12.0 ± 4.4	
**12**	>20	46.5 ± 5.8	***	6.9 ± 2.7	73.7 ± 2.4	***
**13**	13.7 ± 4.4	72.4 ± 5.9	***	4.4 ± 0.7	117.3 ± 3.2	***
Idelalisib *^d^*	0.07 ± 0.01		***	0.30 ± 0.10		***

*^a^* Inh% at 10 μg/mL. *^b^* **1** induced superoxide generation and elastase release in the absence of fMLF/CB. *^c^* NOT tested. *^d^* Positive control. Results are presented as mean ± S.E.M. (n = 3–4). * *p* < 0.05, ** *p* < 0.01, *** *p* < 0.001 compared with the control (DMSO).

## Supplementary Materials

HRESIMS, ^1^H, ^13^C, COSY, heteronuclear single quantum coherence spectroscopy (HSQC), HMBC, and NOESY spectra of new compounds **1**–**11** and ^1^H NMR spectrum of MTPA ester of **1** are available online at https://www.mdpi.com/1660-3397/18/4/223/s1, Figure S1: HRESIMS spectrum of **1**, Figure S2: ^1^H NMR spectrum of **1** in CDCl_3_ at 400 MHz, Figure S3: ^13^C NMR spectrum of **1** in CDCl_3_ at 100 MHz, Figure S4: ^1^H-^1^H COSY spectrum of **1** in CDCl_3_, Figure S5: HSQC spectrum of **1** in CDCl_3_, Figure S6: HMBC spectrum of **1** in CDCl_3_, Figure S7: NOESY spectrum of **1** in CDCl_3_, Figure S8: ^1^H NMR spectrum of (*S*)-MTPA ester of **1** (**1a)** in CDCl_3_ at 400 MHz, Figure S9: ^1^H NMR spectrum of (*R*)-MTPA ester of **1** (**1b)** in CDCl_3_ at 400 MHz, Figure S10: HRESIMS spectrum of **2**, Figure S11: ^1^H NMR spectrum of **2** in CDCl_3_ at 400 MHz, Figure S12: ^13^C NMR spectrum of **2** in CDCl_3_ at 100 MHz, Figure S13: ^1^H-^1^H COSY spectrum of **2** in CDCl_3_, Figure S14: HSQC spectrum of **2** in CDCl_3_, Figure S15: HMBC spectrum of **2** in CDCl_3_, Figure S16: NOESY spectrum of **2** in CDCl_3_, Figure S17: HRESIMS spectrum of **3**, Figure S18: ^1^H NMR spectrum of **3** in CDCl_3_ at 500 MHz, Figure S19: ^13^C NMR spectrum of **3** in CDCl_3_ at 125 MHz, Figure S20: ^1^H-^1^H COSY spectrum of **3** in CDCl_3_, Figure S21: HSQC spectrum of **3** in CDCl_3_, Figure S22: HMBC spectrum of **3** in CDCl_3_, Figure S23: NOESY spectrum of **3** in CDCl_3_, Figure S24: HRESIMS spectrum of **4**, Figure S25: ^1^H NMR spectrum of **4** in CDCl_3_ at 400 MHz, Figure S26: ^13^C NMR spectrum of **4** in CDCl_3_ at 100 MHz, Figure S27: ^1^H-^1^H COSY spectrum of **4** in CDCl_3_, Figure S28: HSQC spectrum of **4** in CDCl_3_, Figure S29: HMBC spectrum of **4** in CDCl_3_, Figure S30: NOESY spectrum of **4** in CDCl_3_, Figure S31: HRESIMS spectrum of **5a** and **5b**, Figure S32: ^1^H NMR spectrum of **5a** and **5b** acetone-*d6* at 500 MHz, Figure S33: ^13^C NMR spectrum of **5a** and **5b** in acetone-*d6* at 125 MHz, Figure S34: ^1^H-^1^H COSY spectrum of **5a** and **5b** in acetone-*d6*, Figure S35: HSQC spectrum of **5a** and **5b** in acetone-*d6*, Figure S36: HMBC spectrum of **5a** and **5b** in acetone-*d6*, Figure S37: NOESY spectrum of **5a** and **5b** in acetone-*d6*, Figure S38: HRESIMS spectrum of **6**, Figure S39: ^1^H NMR spectrum of **6** in CDCl_3_ at 400 MHz, Figure S40: ^13^C NMR spectrum of **6** in CDCl_3_ at 100 MHz, Figure S41: ^1^H-^1^H COSY spectrum of **6** in CDCl_3_, Figure S42: HSQC spectrum of **6** in CDCl_3_, Figure S43: HMBC spectrum of **6** in CDCl_3_, Figure S44: NOESY spectrum of **6** in CDCl_3_, Figure S45: HRESIMS spectrum of **7**, Figure S46: ^1^H NMR spectrum of **7** in CDCl_3_ at 400 MHz, Figure S47: ^13^C NMR spectrum of **7** in CDCl_3_ at 100 MHz, Figure S48: ^1^H-^1^H COSY spectrum of **7** in CDCl_3_, Figure S49: HSQC spectrum of **7** in CDCl_3_, Figure S50: HMBC spectrum of **7** in CDCl_3_, Figure S51: NOESY spectrum of **7** in CDCl_3_, Figure S52: HRESIMS spectrum of **8**, Figure S53: ^1^H NMR spectrum of **8** in CDCl_3_ at 400 MHz, Figure S54: ^13^C NMR spectrum of **8** in CDCl_3_ at 100 MHz, Figure S55: ^1^H-^1^H COSY spectrum of **8** in CDCl_3_, Figure S56: HSQC spectrum of **8** in CDCl_3_, Figure S57: HMBC spectrum of **8** in CDCl_3_, Figure S58: NOESY spectrum of **8** in CDCl_3_, Figure S59: HRESIMS spectrum of **9**, Figure S60: ^1^H NMR spectrum of **9** in CDCl_3_ at 400 MHz, Figure S61: ^13^C NMR spectrum of **9** in CDCl_3_ at 100 MHz, Figure S62: ^1^H-^1^H COSY spectrum of **9** in CDCl_3_, Figure S63: HSQC spectrum of **9** in CDCl_3_, Figure S64: HMBC spectrum of **9** in CDCl_3_, Figure S65: NOESY spectrum of **9** in CDCl_3_, Figure S66: HRESIMS spectrum of **10**, Figure S67: ^1^H NMR spectrum of **10** in CDCl_3_ at 500 MHz, Figure S68: ^13^C NMR spectrum of **10** in CDCl_3_ at 125 MHz, Figure S69: ^1^H-^1^H COSY spectrum of **10** in CDCl_3_, Figure S70: HSQC spectrum of **10** in CDCl_3_, Figure S71: HMBC spectrum of **10** in CDCl_3_, Figure S72: NOESY spectrum of **10** in CDCl_3_, Figure S73: HRESIMS spectrum of **11**, Figure S74: ^1^H NMR spectrum of **11** in CDCl_3_ at 500 MHz, Figure S75: ^13^C NMR spectrum of **11** in CDCl_3_ at 125 MHz, Figure S76: ^1^H-^1^H COSY spectrum of **11** in CDCl_3_, Figure S77: HSQC spectrum of **11** in CDCl_3_, Figure S78: HMBC spectrum of **11** in CDCl_3_, Figure S79: NOESY spectrum of **11** in CDCl_3_.
