# Supplementary material for: Isolation of Lobane and Prenyleudesmane Diterpenoids from the Soft Coral Lobophytum varium
Source: Mar Drugs. 2020 Apr 22;18(4):223. doi: 10.3390/md18040223 (PMC7230303; doi:10.3390/md18040223)
Supplement: Supplementary file 1 [file marinedrugs-18-00223-s001.pdf]

Isolation of Lobane and Prenyleudesmane Diterpenoids from the Soft Coral *Lobophytum varium*

Chuan-Hsiang Chang [1,†], Atallah F. Ahmed [2,3,†], Tian-Sheng Yang [1,†], You-Cheng Lin [4], Chiung-Yao Huang [1], Tsong-Long Hwang [5,6,7] and Jyh-Horng Sheu [1,4,8,9,10,*]

[1] Department of Marine Biotechnology and Resources, National Sun Yat-sen University, Kaohsiung 804, Taiwan; hunter02141991@gmail.com (C.-H. C.); b025020004@student.nsysu.edu.tw (T.-S. Y.); betty8575@yahoo.com.tw (C.-Y. H.)
[2] Department of Pharmacognosy, College of Pharmacy, King Saud University, Riyadh 11451, Saudi Arabia; afahmed@ksu.edu.sa
[3] Department of Pharmacognosy, Faculty of Pharmacy, Mansoura University, Mansoura 35516, Egypt
[4] Doctoral Degree Program in Marine Biotechnology, National Sun Yat-sen University, Kaohsiung 804, Taiwan; cand93@yahoo.com.tw
[5] Graduate Institute of Natural Products, College of Medicine, Chang Gung University, Taoyuan 333, Taiwan; htl@mail.cgu.edu.tw
[6] Research Center for Chinese Herbal Medicine, Research Center for Food and Cosmetic Safety, and Graduate Institute of Health Industry Technology, College of Human Ecology, Chang Gung University of Science and Technology, Taoyuan 333, Taiwan
[7] Department of Anesthesiology, Chang Gung Memorial Hospital, Taoyuan 333, Taiwan Graduate Institute of Natural Products, College of Medicine, Chang Gung University, Taoyuan 333, Taiwan
[8] Department of Medical Research, China Medical University Hospital, China Medical University, Taichung 404, Taiwan
[9] Graduate Institute of Natural Products, Kaohsiung Medical University, Kaohsiung 807, Taiwan
[10] Frontier Center for Ocean Science and Technology, National Sun Yat-sen University, Kaohsiung 804, Taiwan
* Correspondence: sheu@mail.nsysu.edu.tw; Tel.: +886-7-5252000 (ext. 5030); Fax: +886-7-5255020
† These authors contributed equally to this work

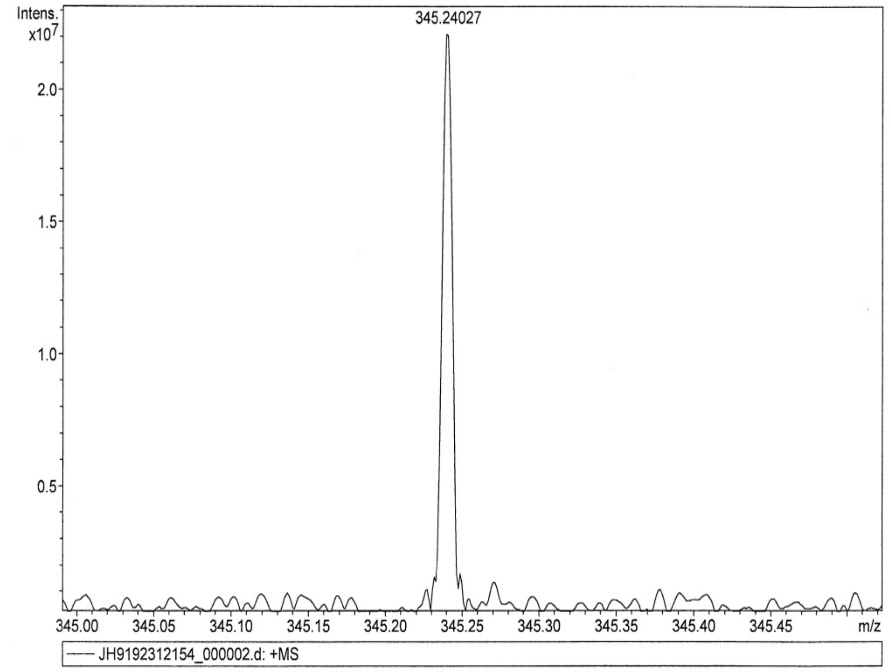

Meas. m/z	#	Formula	Score	m/z	err [mDa]	err [ppm]	mSigma	rdb	e⁻ Conf	N-Rule
345.24027	1	C 20 H 34 Na O 3	100.00	345.24002	-0.25	-0.73	18.4	3.5	even	ok

Figure S1. HRESIMS spectrum of **1**.

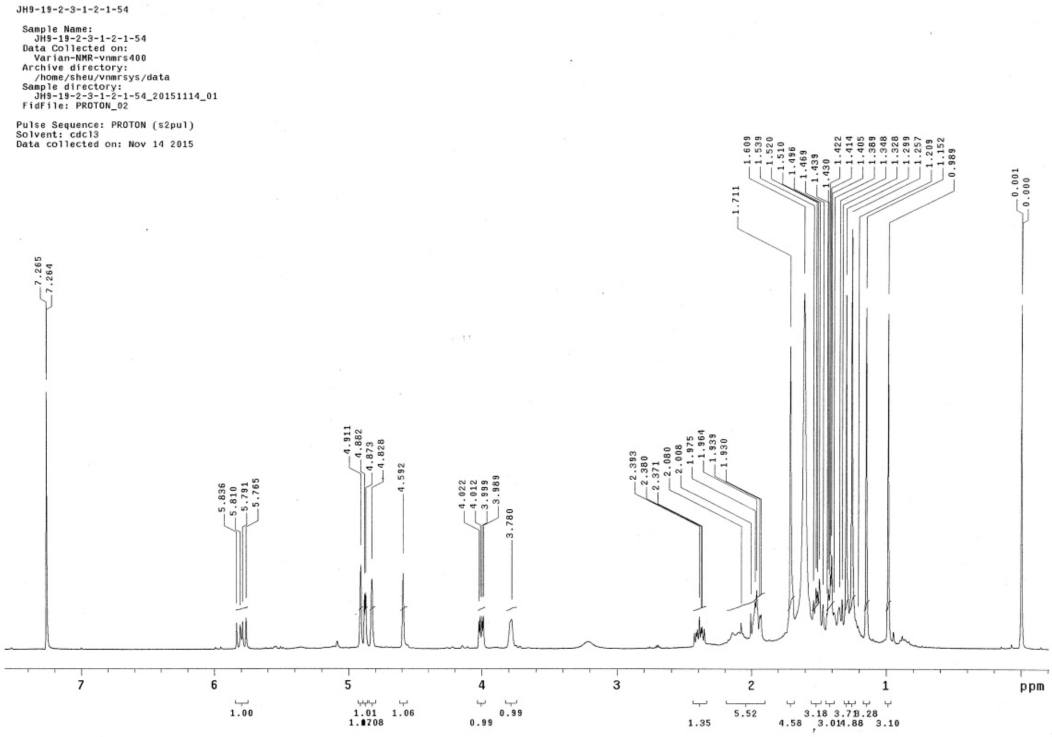

Figure S2. ^{1}H NMR spectrum of **1** in $CDCl_3$ at 400 MHz

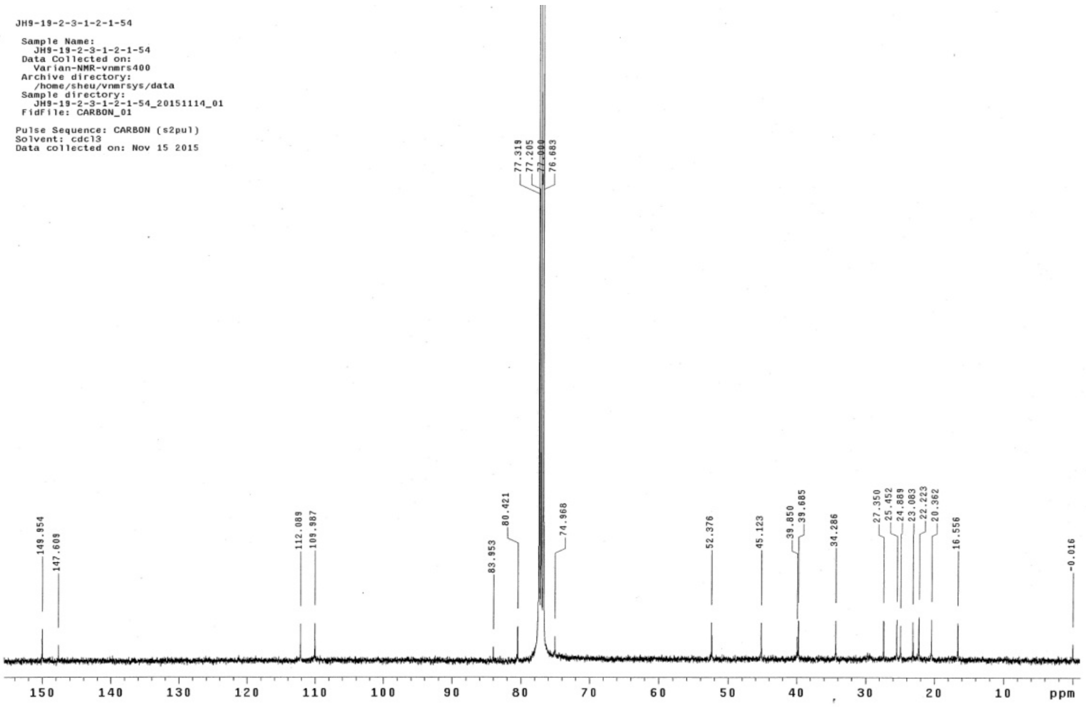

Figure S3. ^{13}C NMR spectrum of **1** in $CDCl_3$ at 100 MHz.

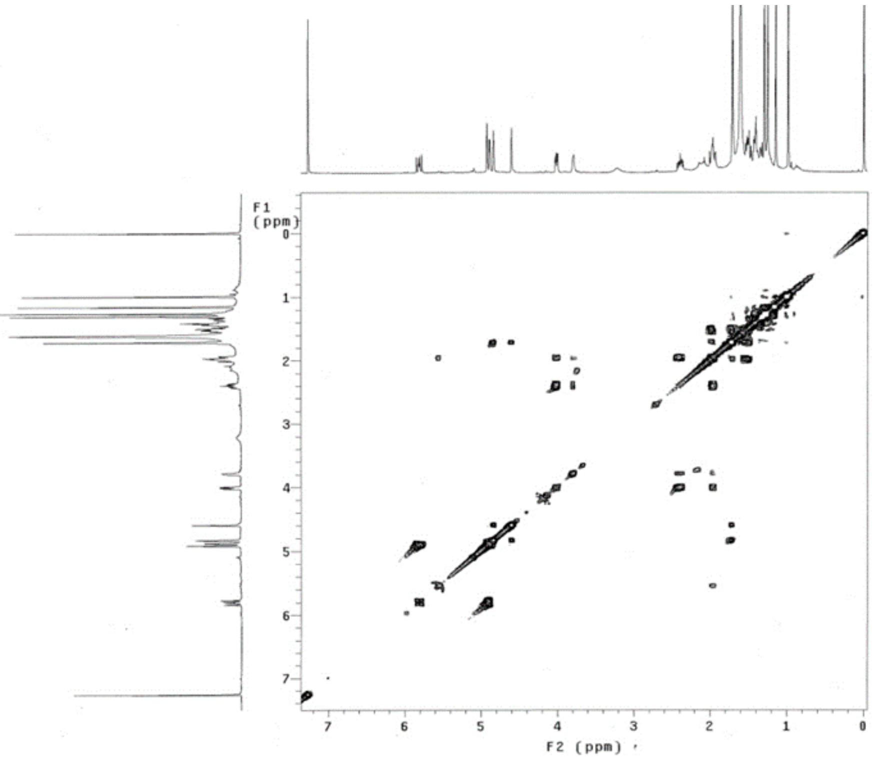

Figure S4. ^{1}H-^{1}H COSY spectrum of **1** in $CDCl_3$.

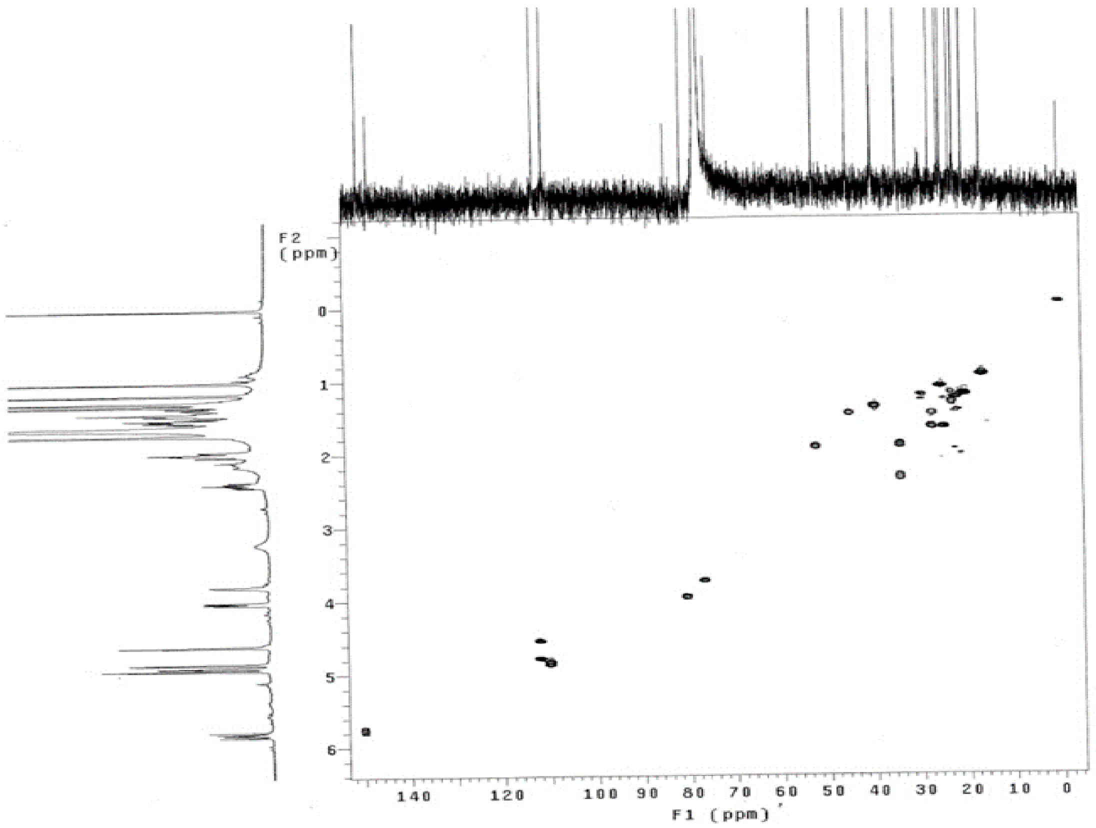

Figure S5. HSQC spectrum of **1** in $CDCl_3$.

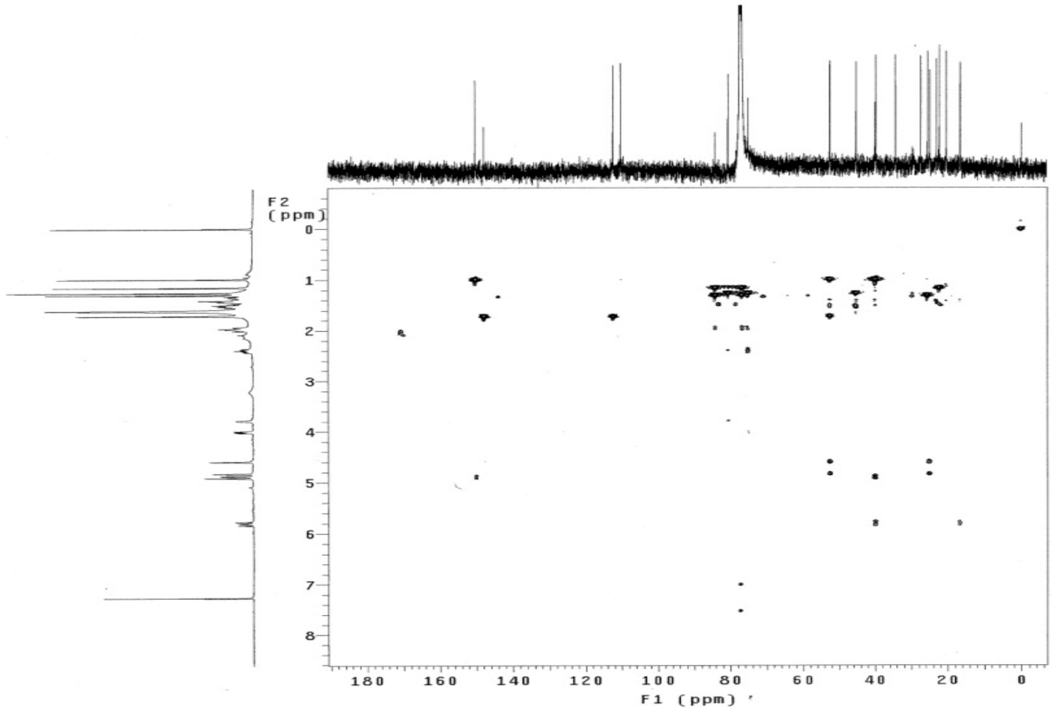

Figure S6. HMBC spectrum of **1** in $CDCl_3$.

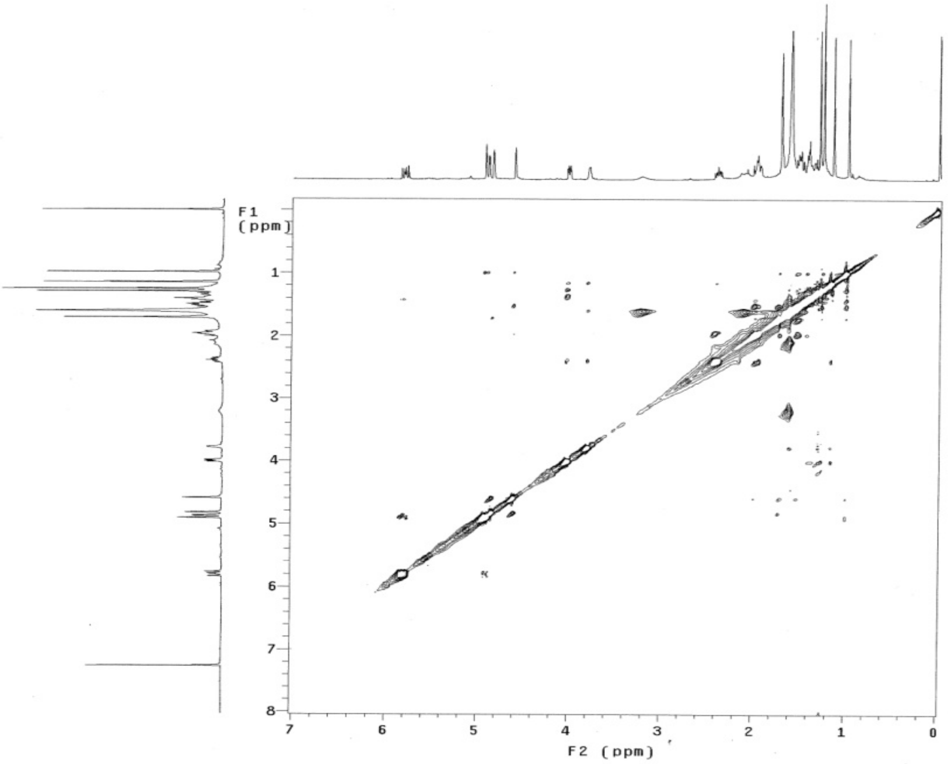

Figure S7. NOESY spectrum of **1** in $CDCl_3$.

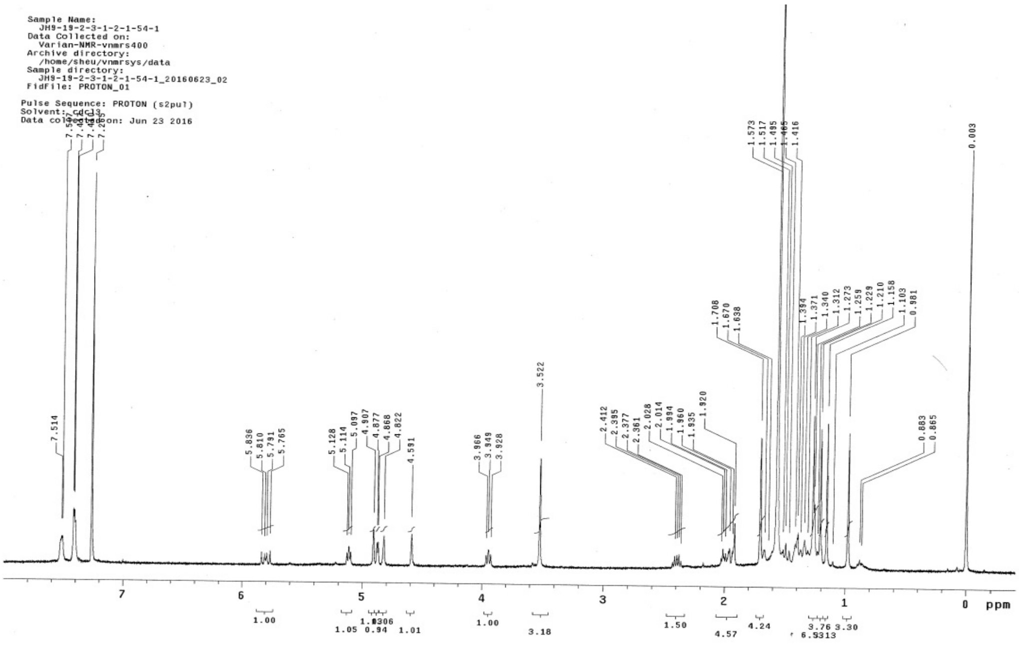

Figure S8. ^{1}H NMR spectrum of (*S*)-MTPA ester of **1** **(1a)** in $CDCl_3$ at 400 MHz.

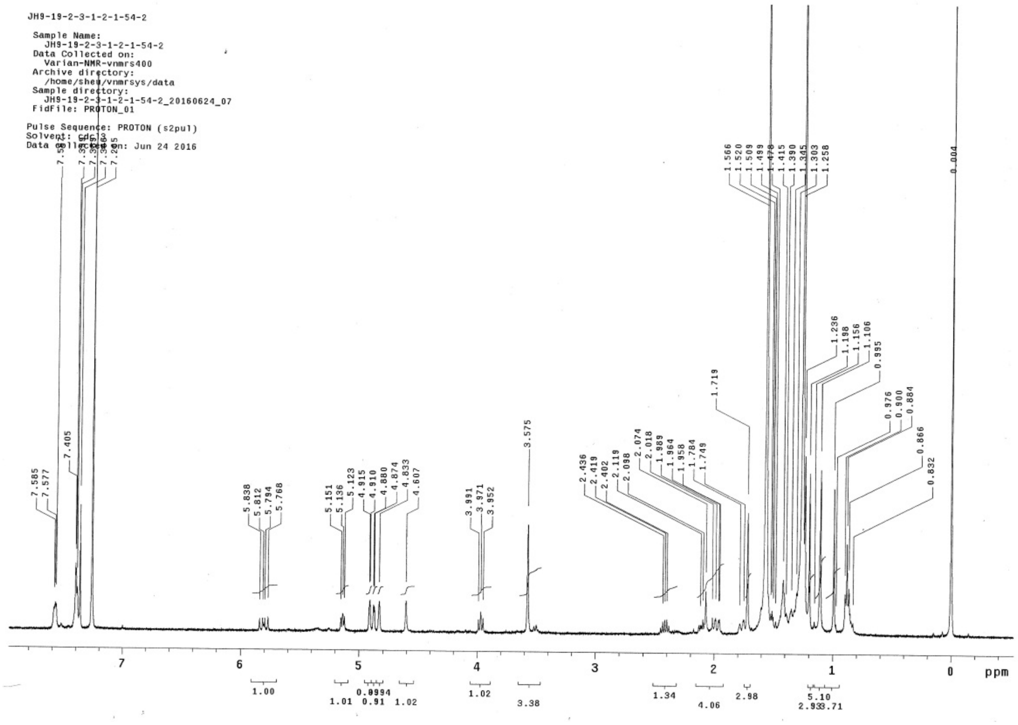

Figure S9. ^{1}H NMR spectrum of (*R*)-MTPA ester of **1** (**1b**) in $CDCl_3$ at 400 MHz.

Figure S10. HRESIMS spectrum of **2**.

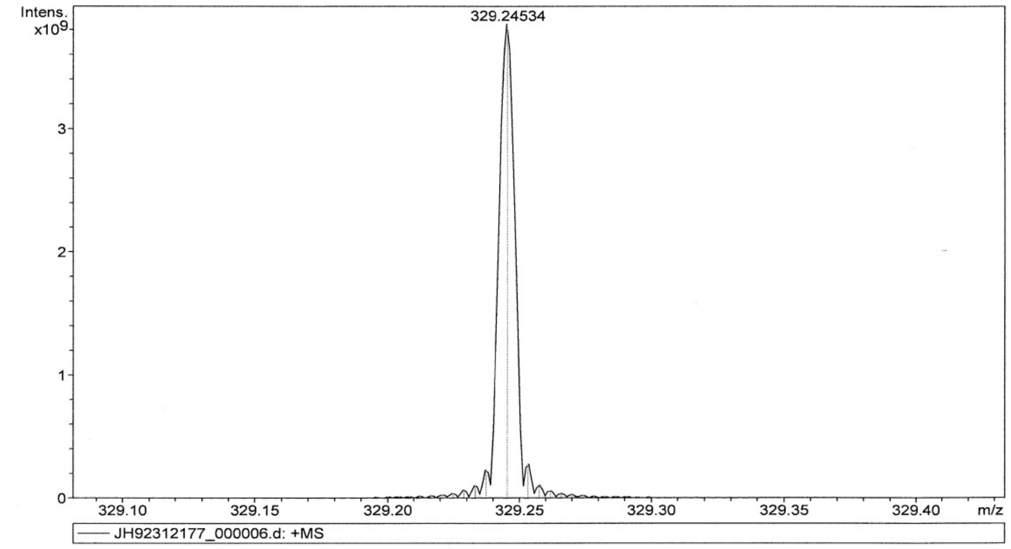

Meas. m/z	#	Formula	Score	m/z	err [mDa]	err [ppm]	mSigma	rdb	e⁻ Conf	N-Rule
329.24534	1	C 20 H 34 Na O 2	100.00	329.24510	-0.24	-0.73	2.7	3.5	even	ok

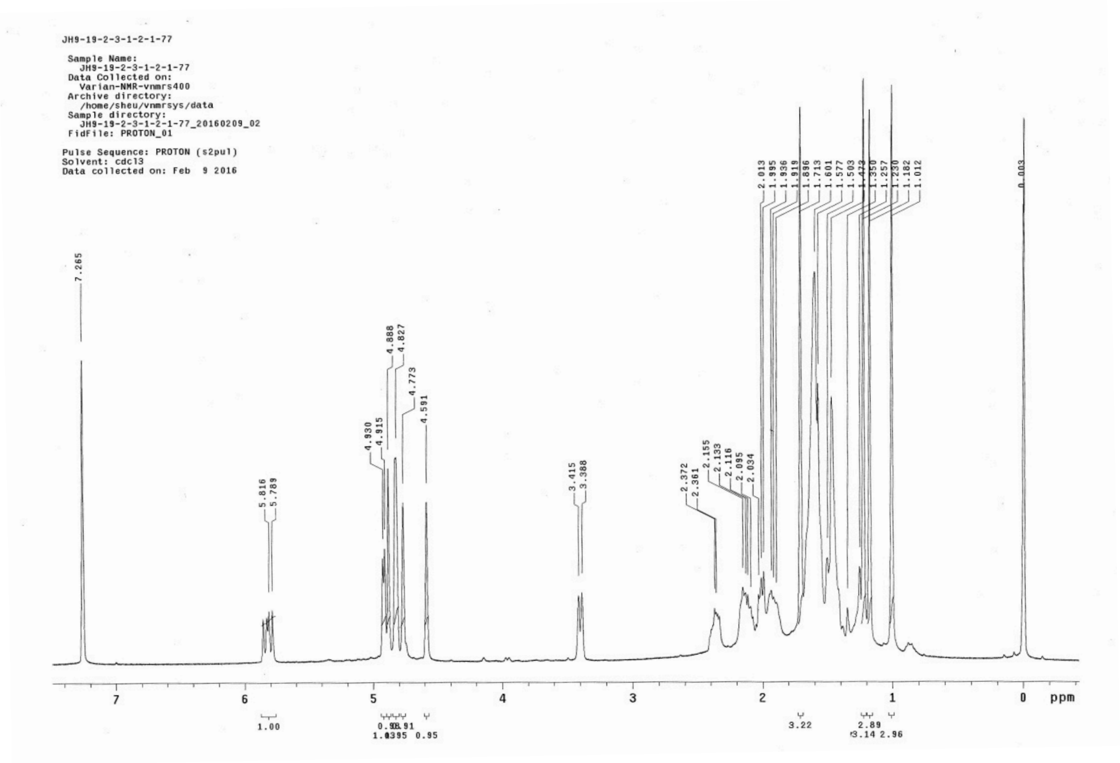

Figure S11. ^{1}H NMR spectrum of **2** in $CDCl_3$ at 400 MHz.

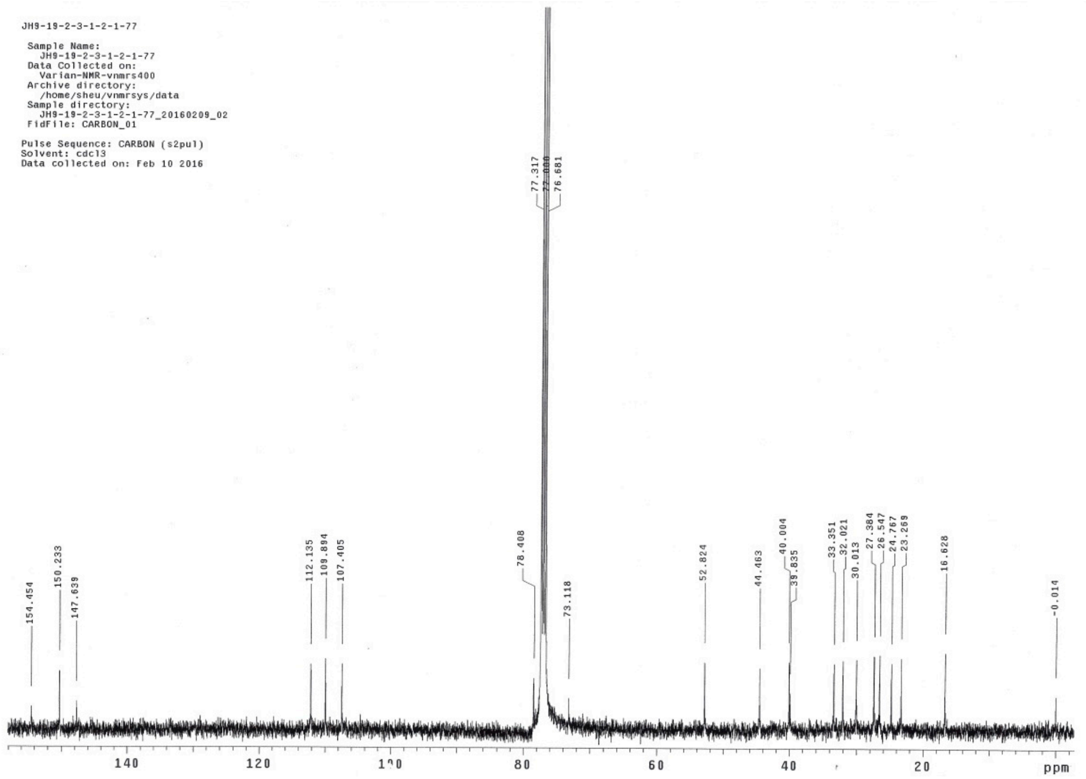

Figure S12. ^{13}C NMR spectrum of **2** in $CDCl_3$ at 100 MHz.

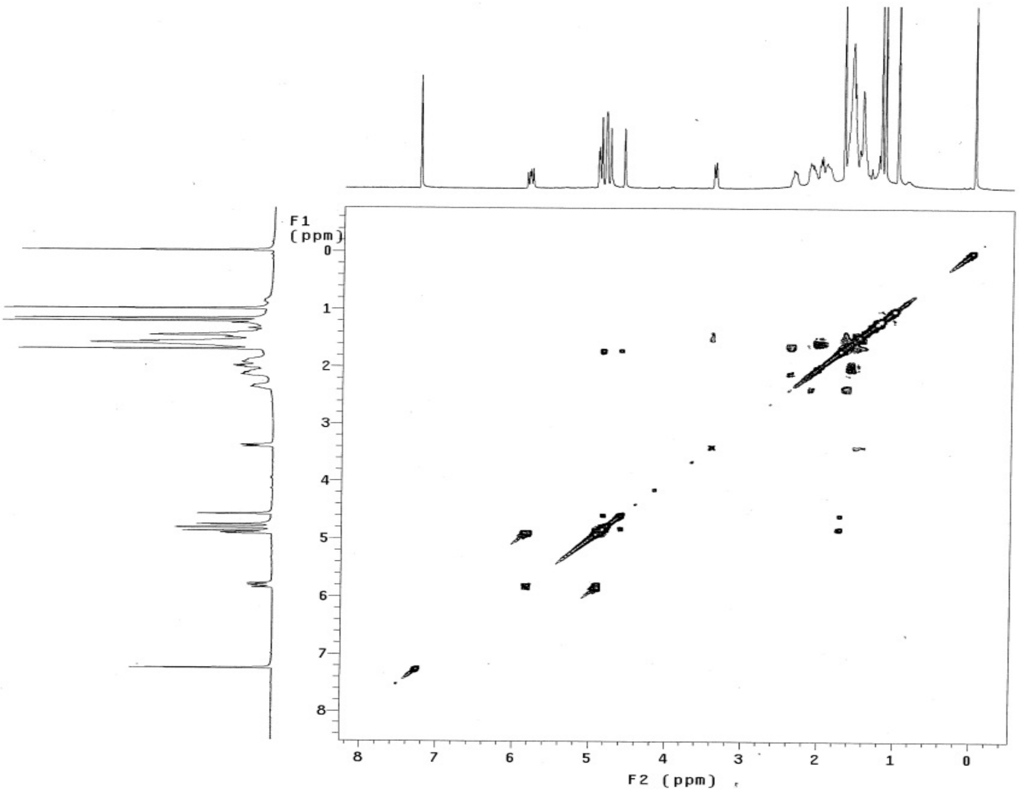

Figure S13. ^1H-^1H COSY spectrum of **2** in $CDCl_3$..

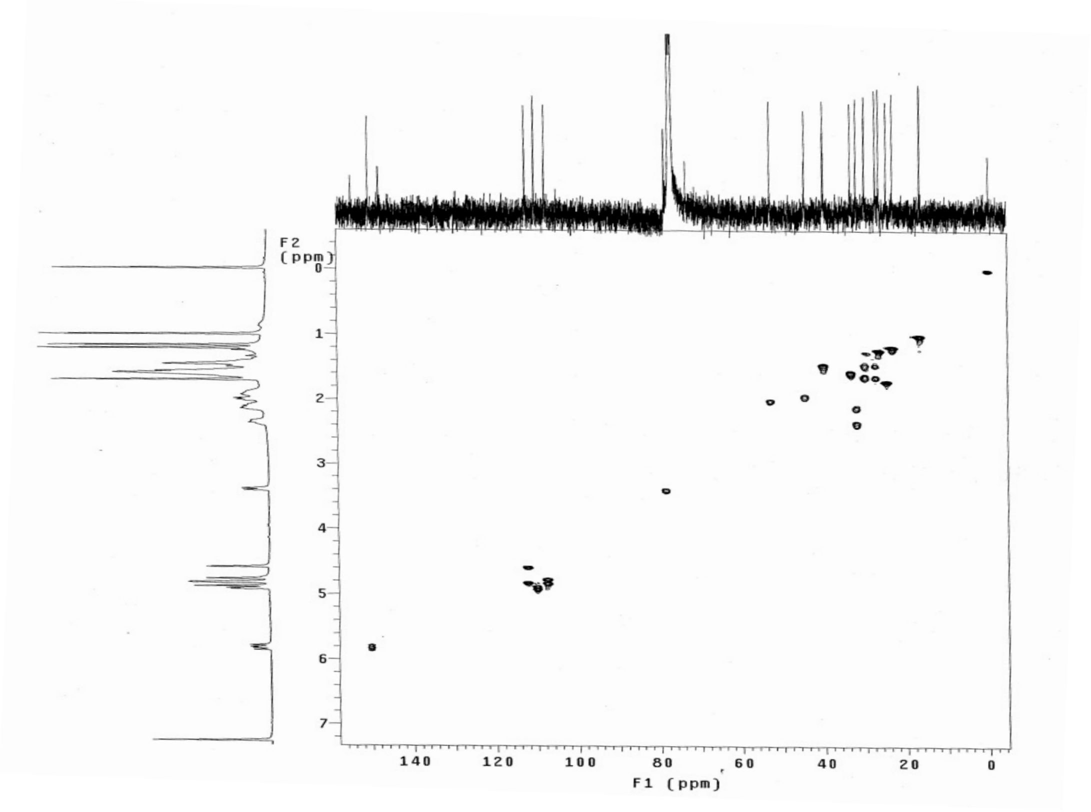

Figure S14. HSQC spectrum of **2** in $CDCl_3$.

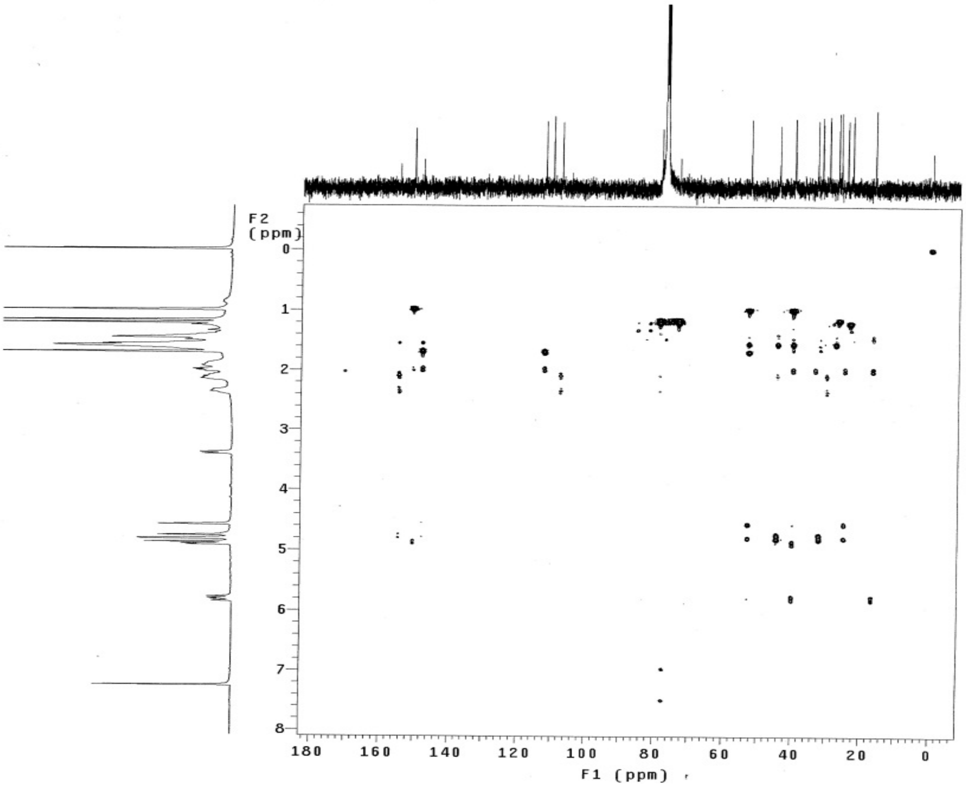

Figure S15. HMBC spectrum of **2** in $CDCl_3$.

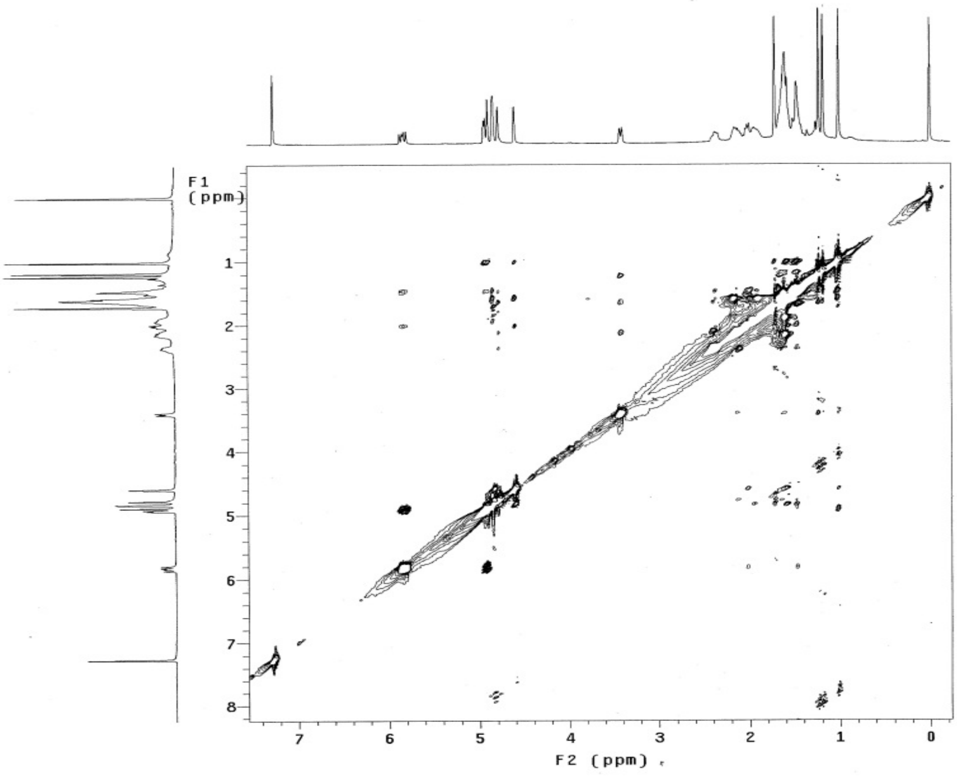

Figure S16. NOESY spectrum of **2** in $CDCl_3$.

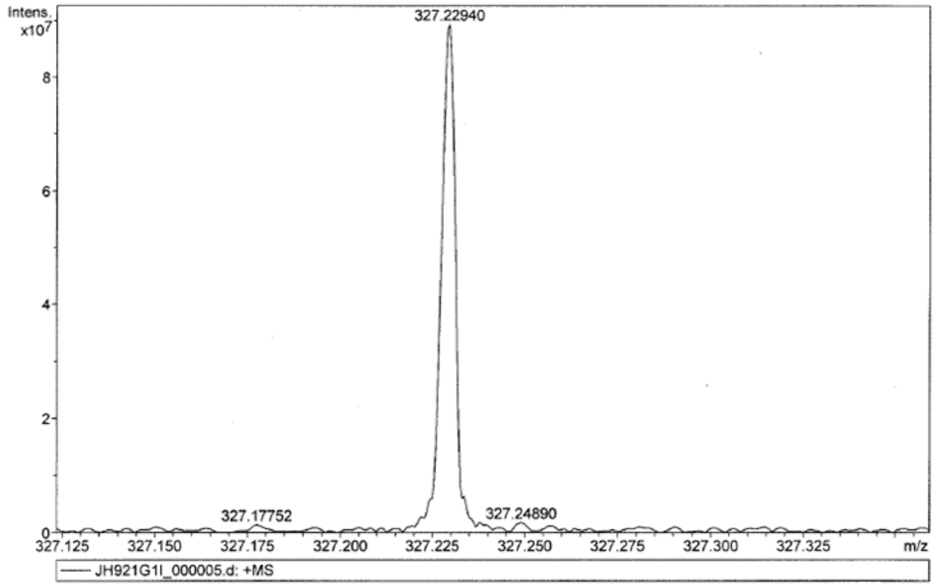

Meas. m/z	#	Formula	Score	m/z	err [mDa]	err [ppm]	mSigma	rdb	e⁻ Conf	N-Rule
327.22940	1	C 20 H 32 Na O 2	100.00	327.22945	0.05	0.15	7.5	4.5	even	ok

Figure S17. HRESIMS spectrum of **3**.

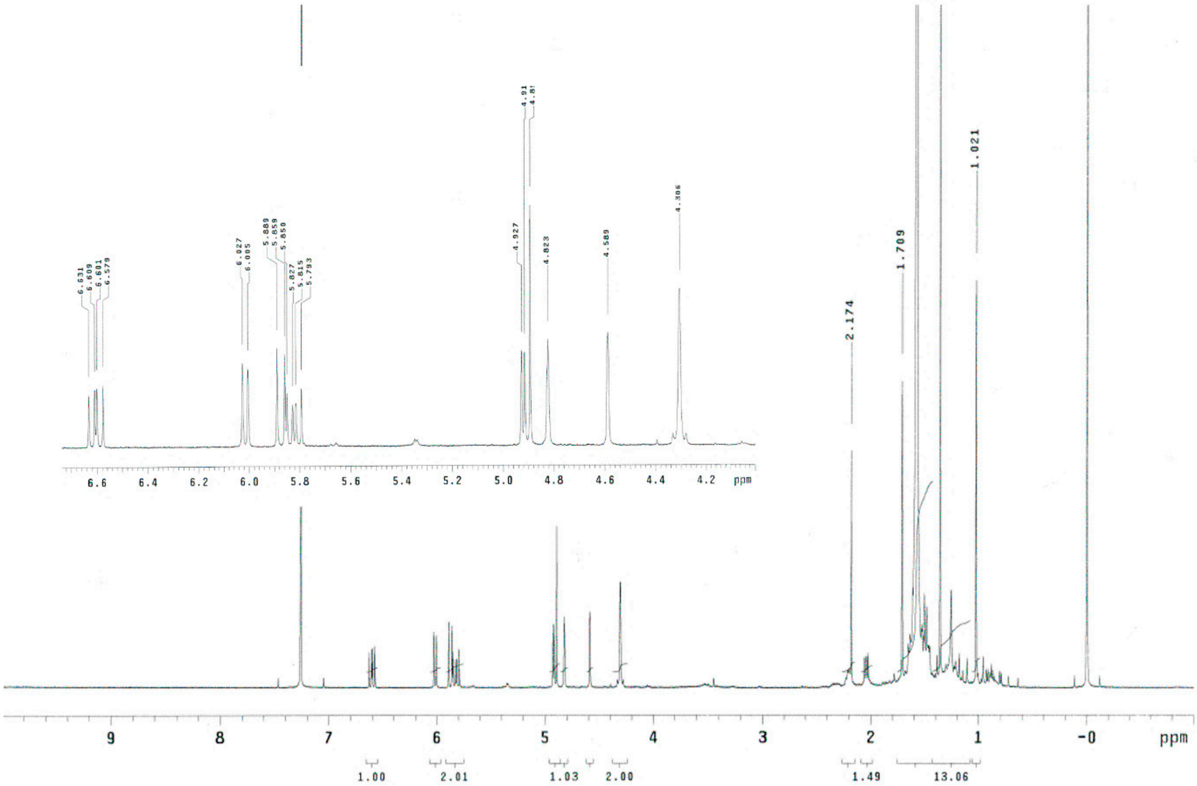

Figure S18. ^{1}H NMR spectrum of **3** in $CDCl_3$ at 500 MHz.

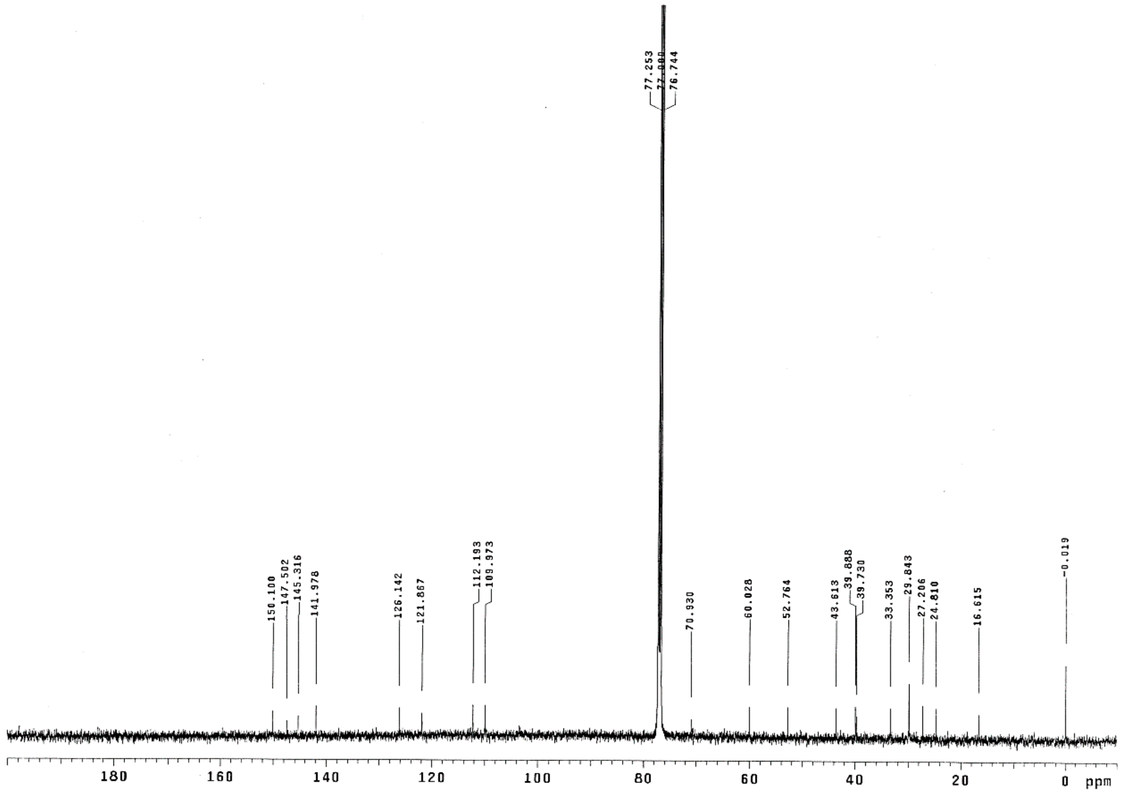

Figure S19. ^{13}C NMR spectrum of **3** in $CDCl_3$ at 125 MHz.

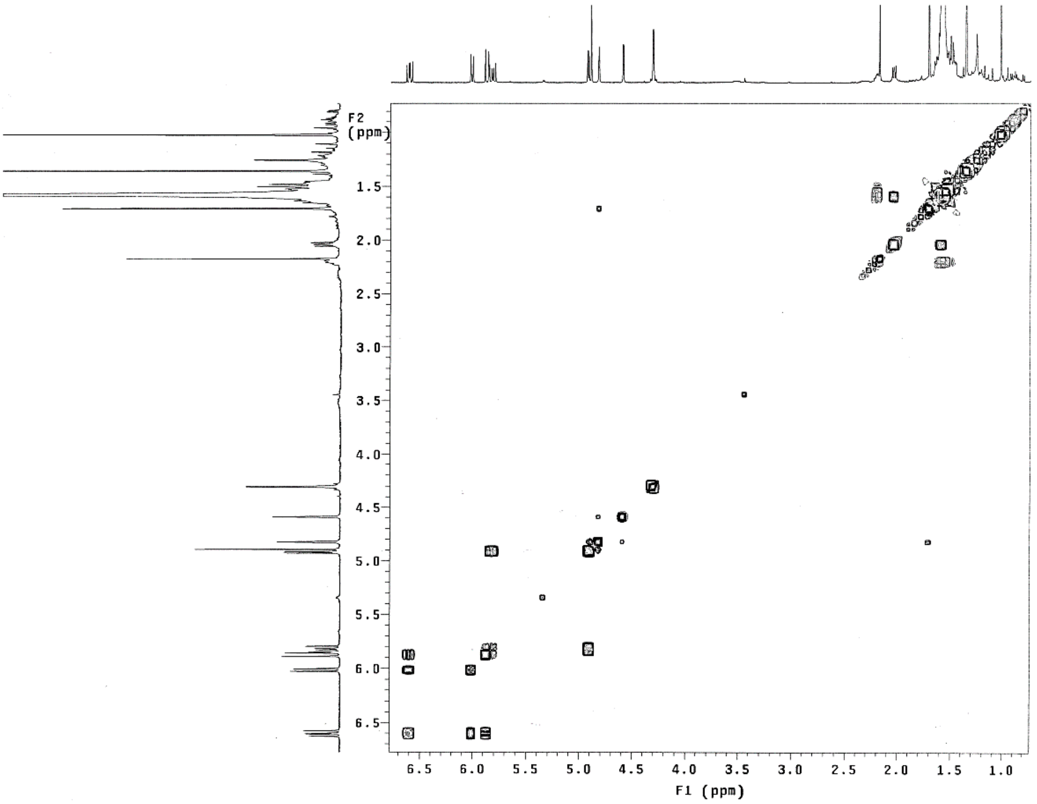

Figure S20. ^{1}H-^{1}H COSY spectrum of **3** in $CDCl_3$.

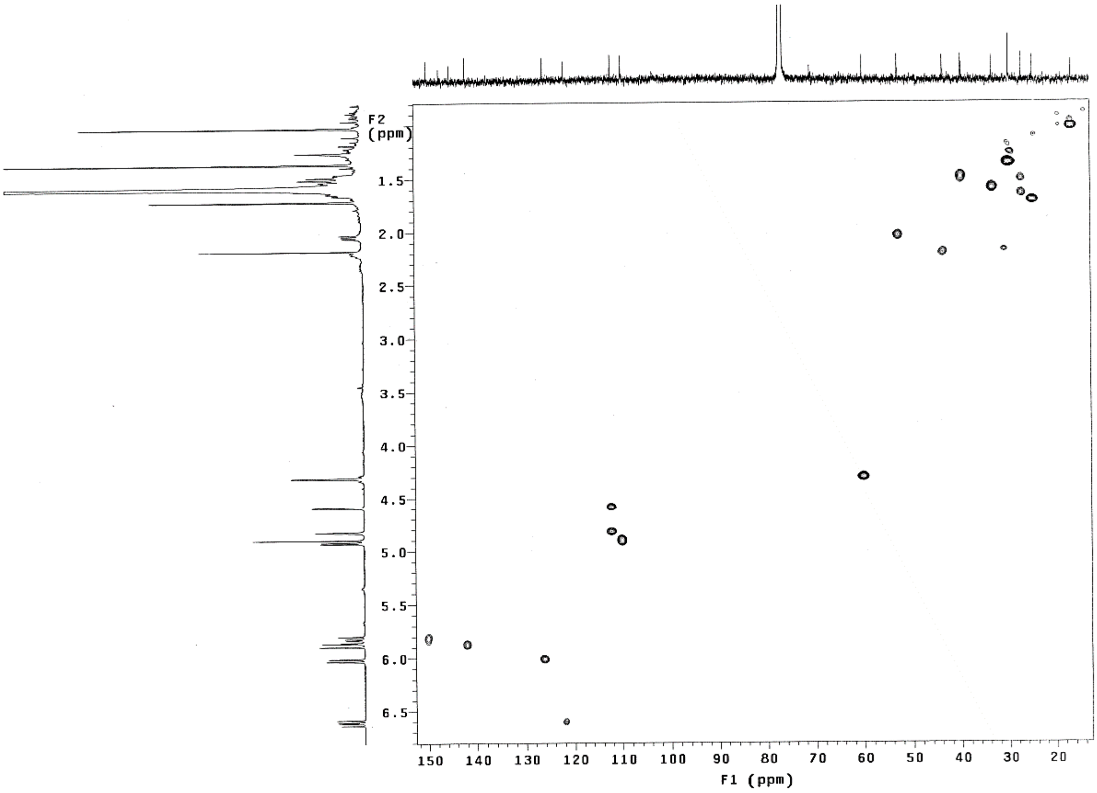

Figure S21. HSQC spectrum of **3** in $CDCl_3$.

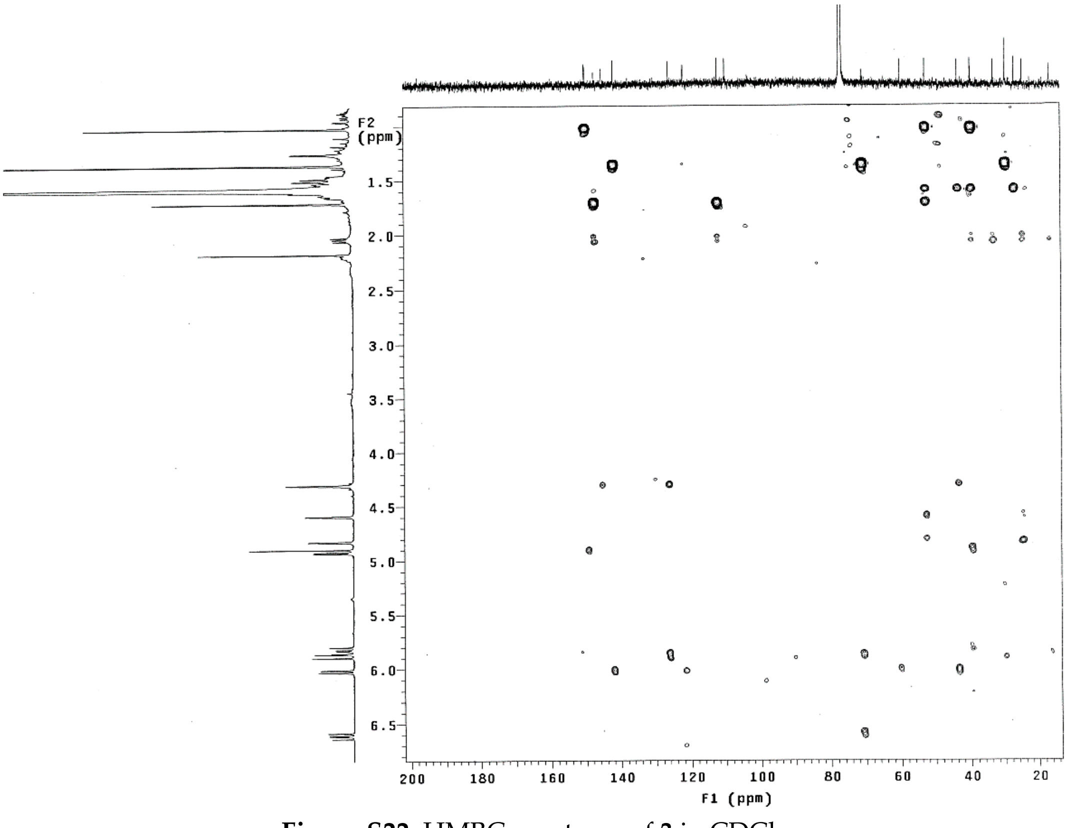

Figure S22. HMBC spectrum of **3** in $CDCl_3$.

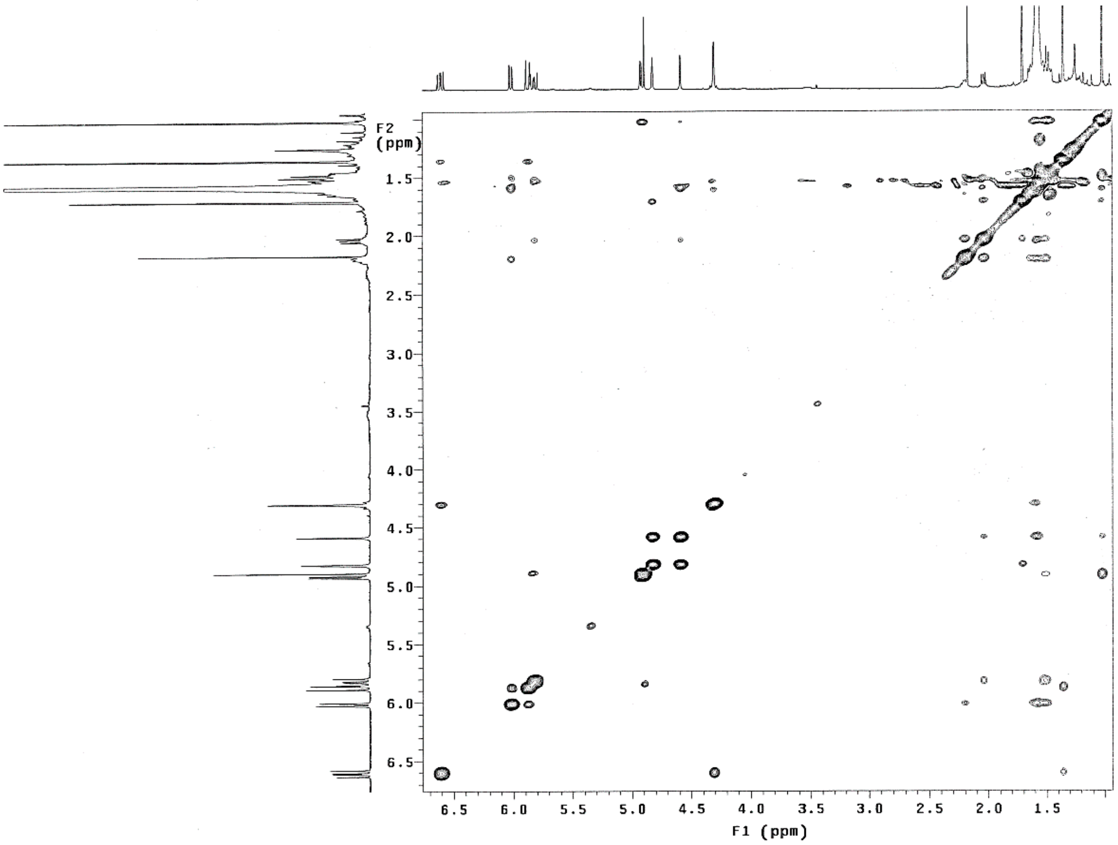

Figure S23. NOESY spectrum of **3** in $CDCl_3$.

Figure S24. HRESIMS spectrum of **4**.

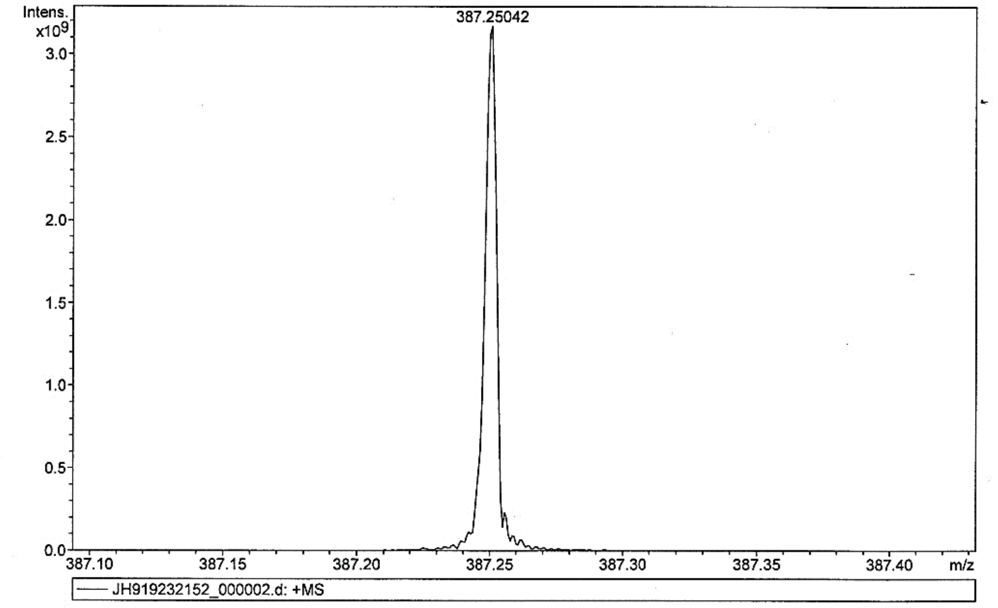

Meas. m/z	#	Formula	Score	m/z	err [mDa]	err [ppm]	mSigma	rdb	e⁻ Conf	N-Rule
387.25042	1	C 22 H 36 Na O 4	100.00	387.25058	0.16	0.41	7.4	4.5	even	ok

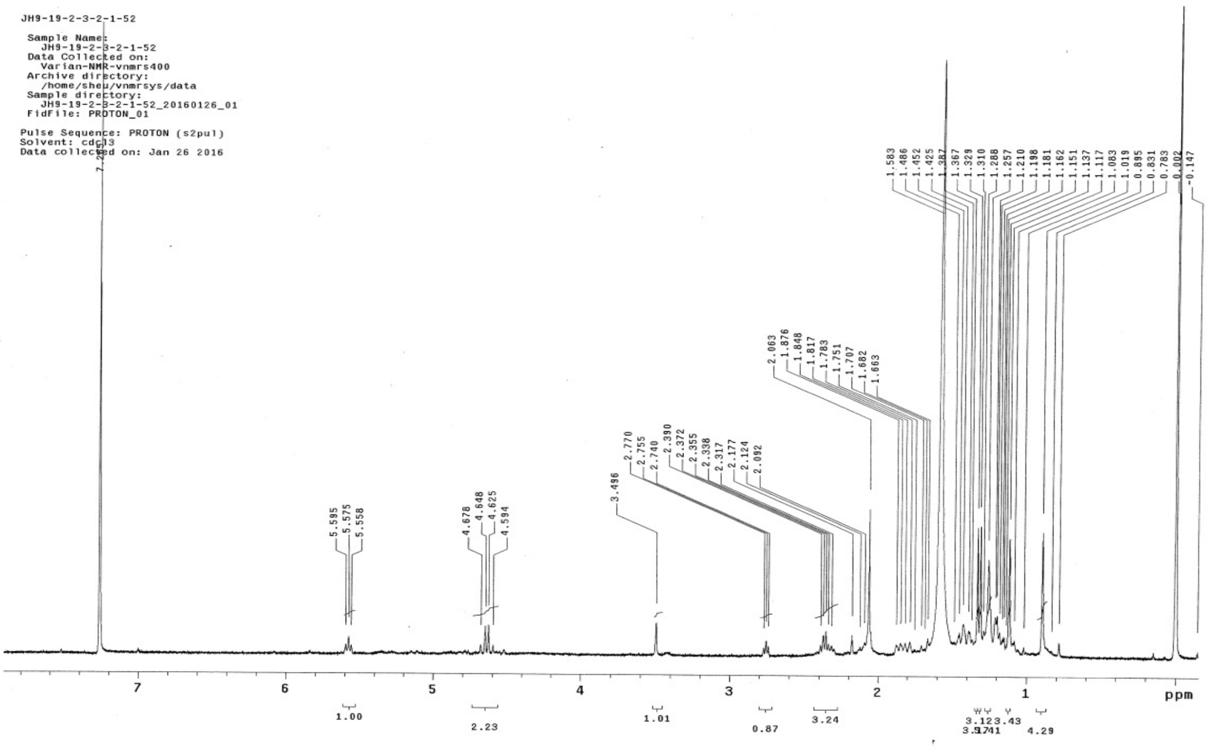

Figure S25. ^{1}H NMR spectrum of **4** in $CDCl_3$ at 400 MHz.

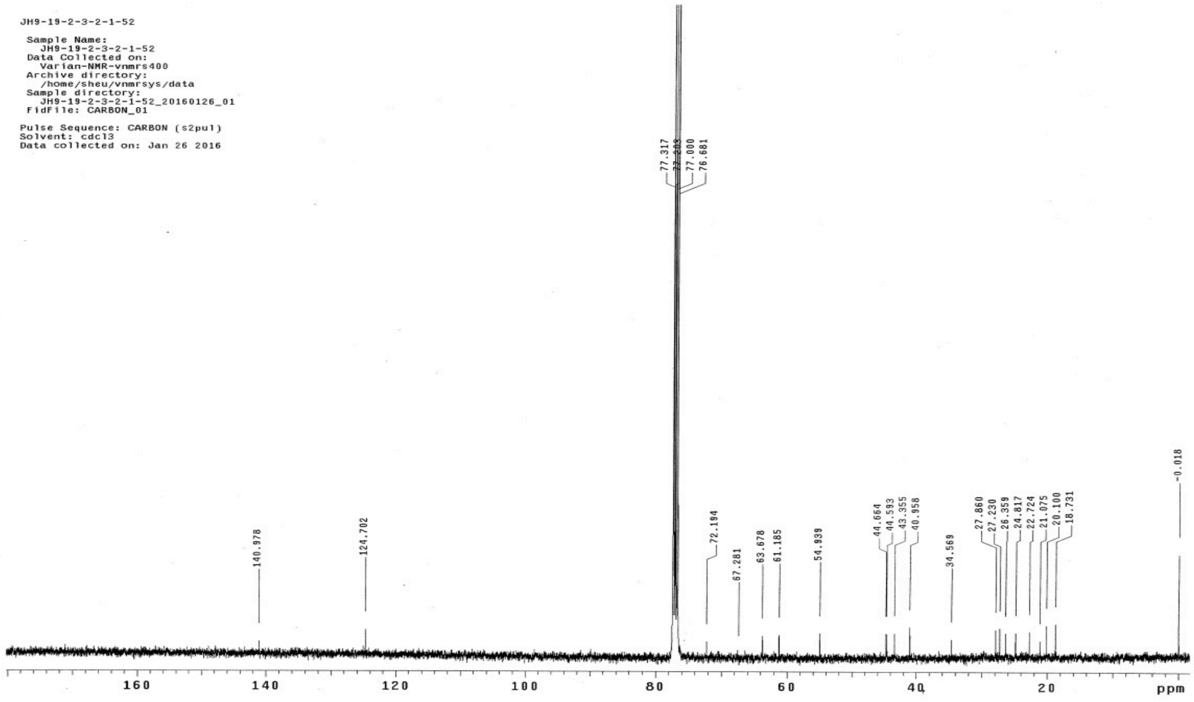

Figure S26. ^{13}C NMR spectrum of **4** in $CDCl_3$ at 100 MHz.

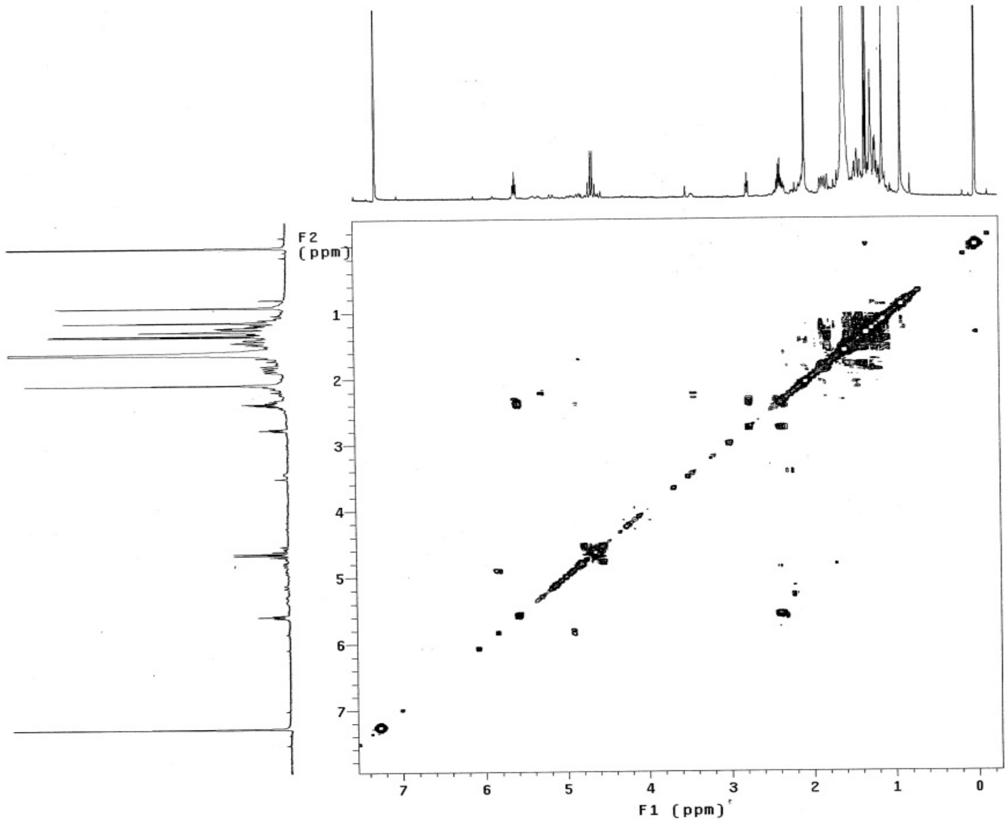

Figure S27. ^{1}H-^{1}H COSY spectrum of **4** in $CDCl_3$.

Figure S28. HSQC spectrum of **4** in $CDCl_3$.

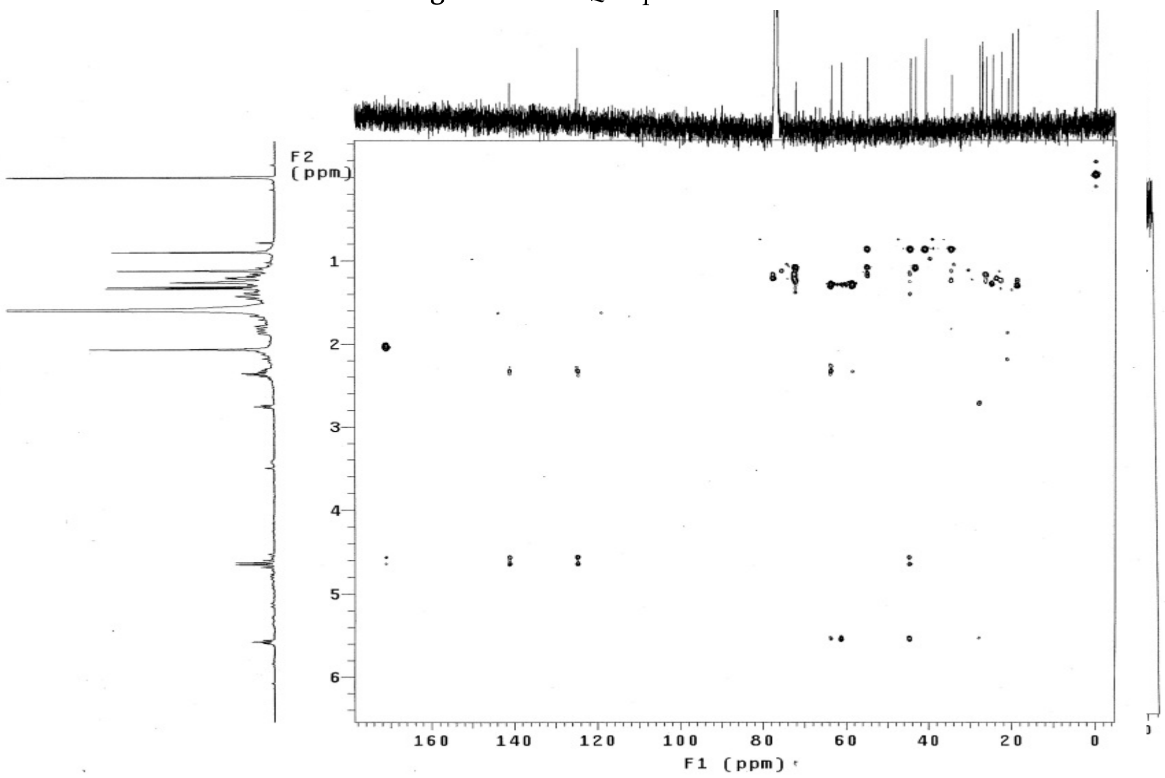

Figure S29. HMBC spectrum of **4** in $CDCl_3$.

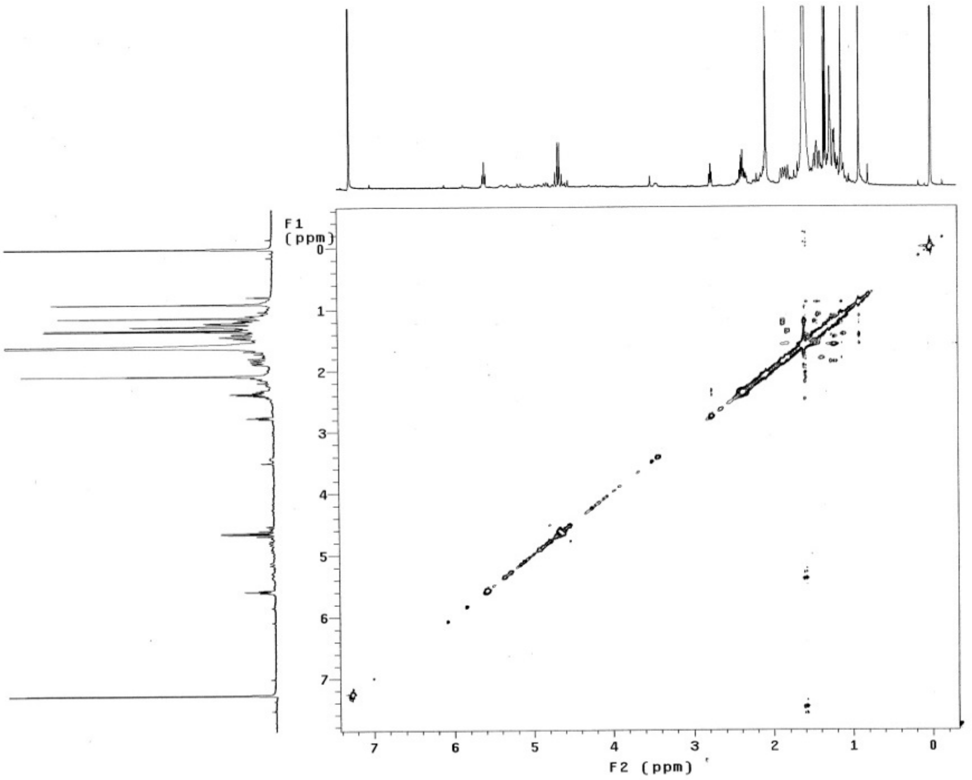

Figure S30. NOESY spectrum of **4** in $CDCl_3$.

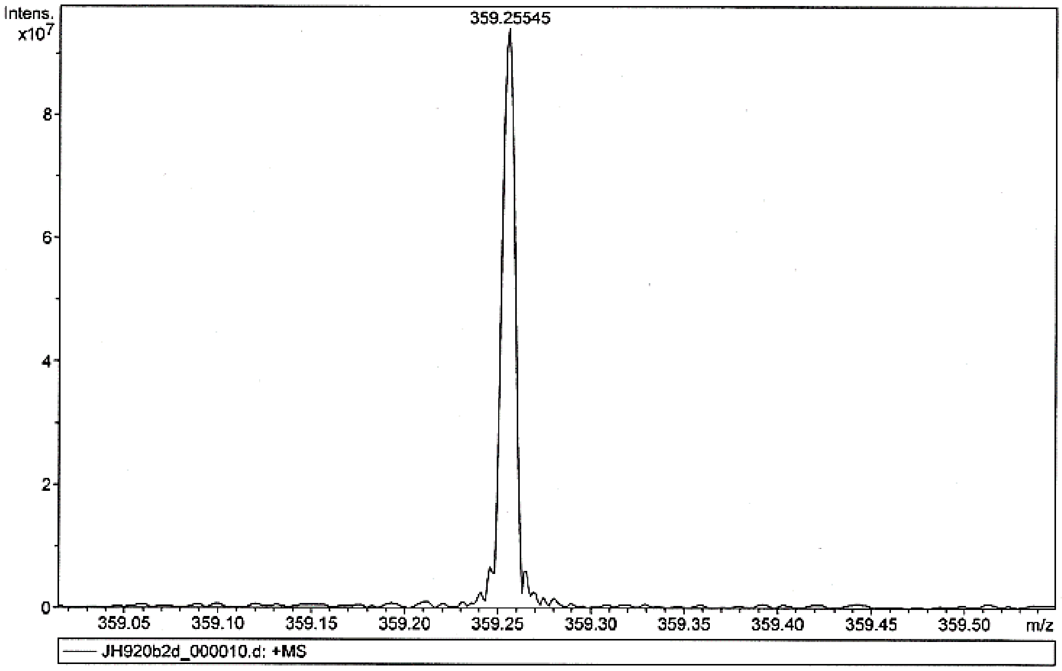

Meas. m/z	#	Formula	Score	m/z	err [mDa]	err [ppm]	mSigma	rdb	e⁻ Conf	N-Rule
359.25545	1	C 21 H 36 Na O 3	100.00	359.25567	0.22	0.61	0.6	3.5	even	ok

Figure S31. HRESIMS spectrum of **5a** and **5b**.

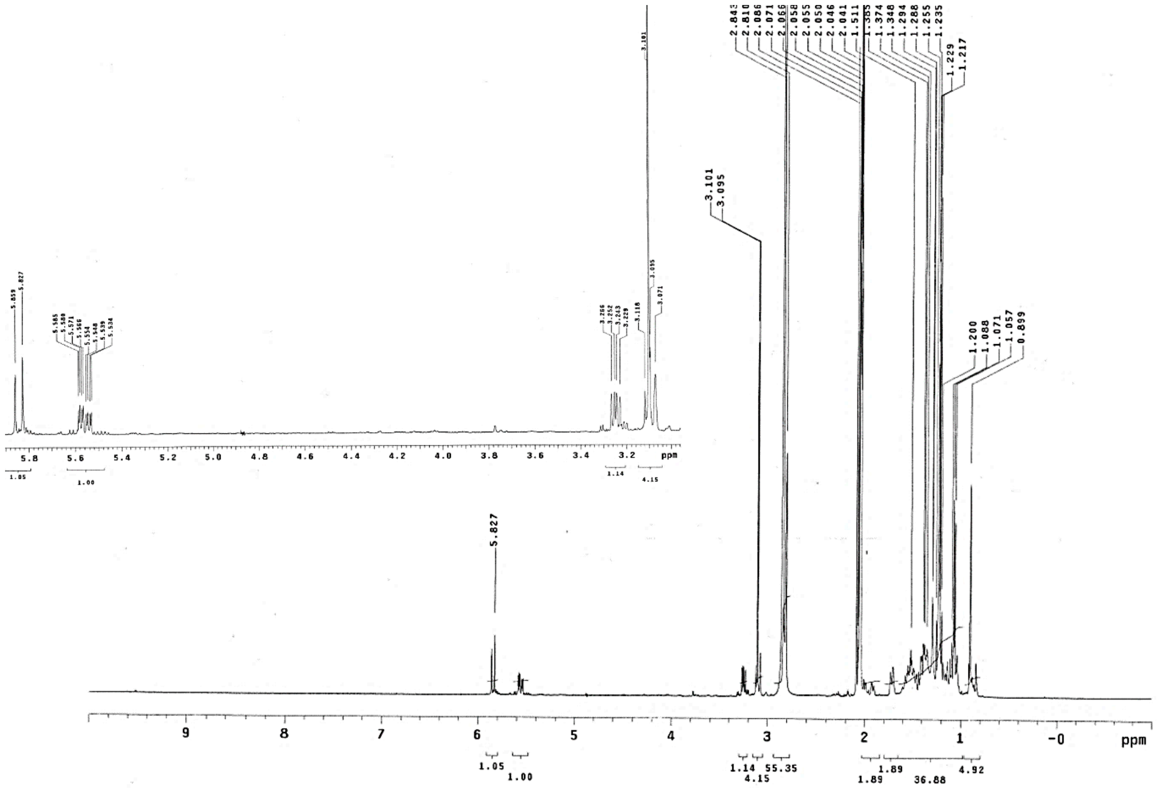

Figure S32. ^{1}H NMR spectrum of **5a** and **5b** in acetone-d_6 at 500 MHz.

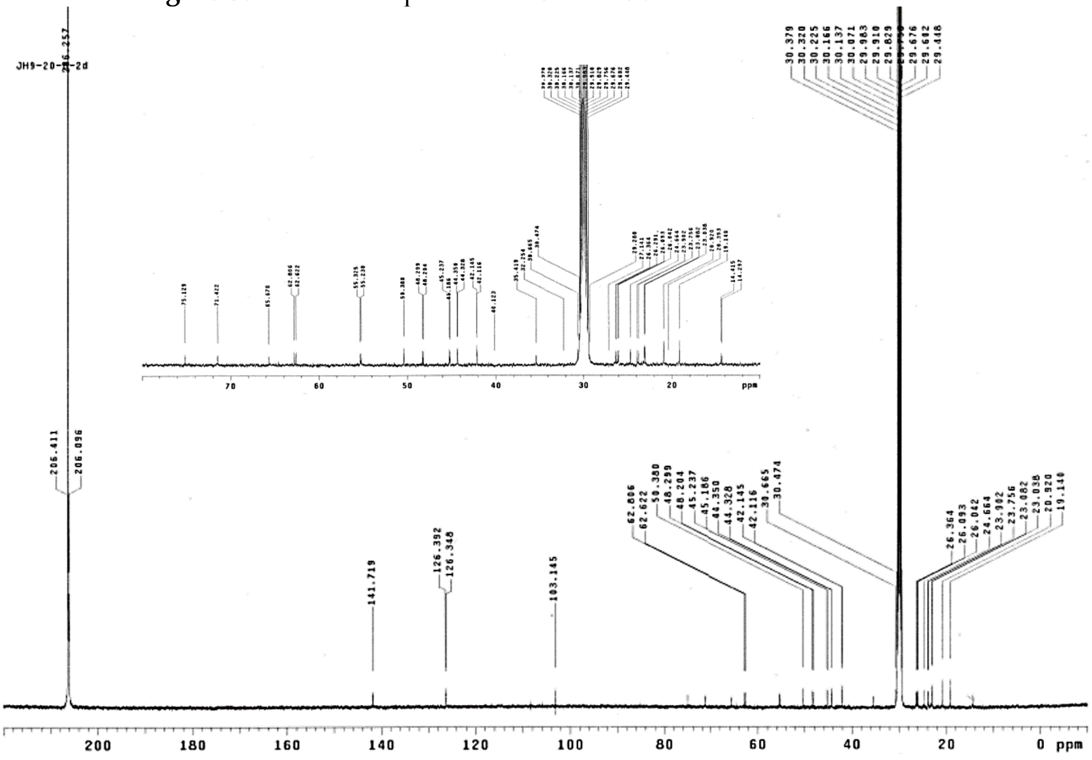

Figure S33. ^{13}C NMR spectrum of **5a** and **5b** in acetone-d_6 at 125 MHz.

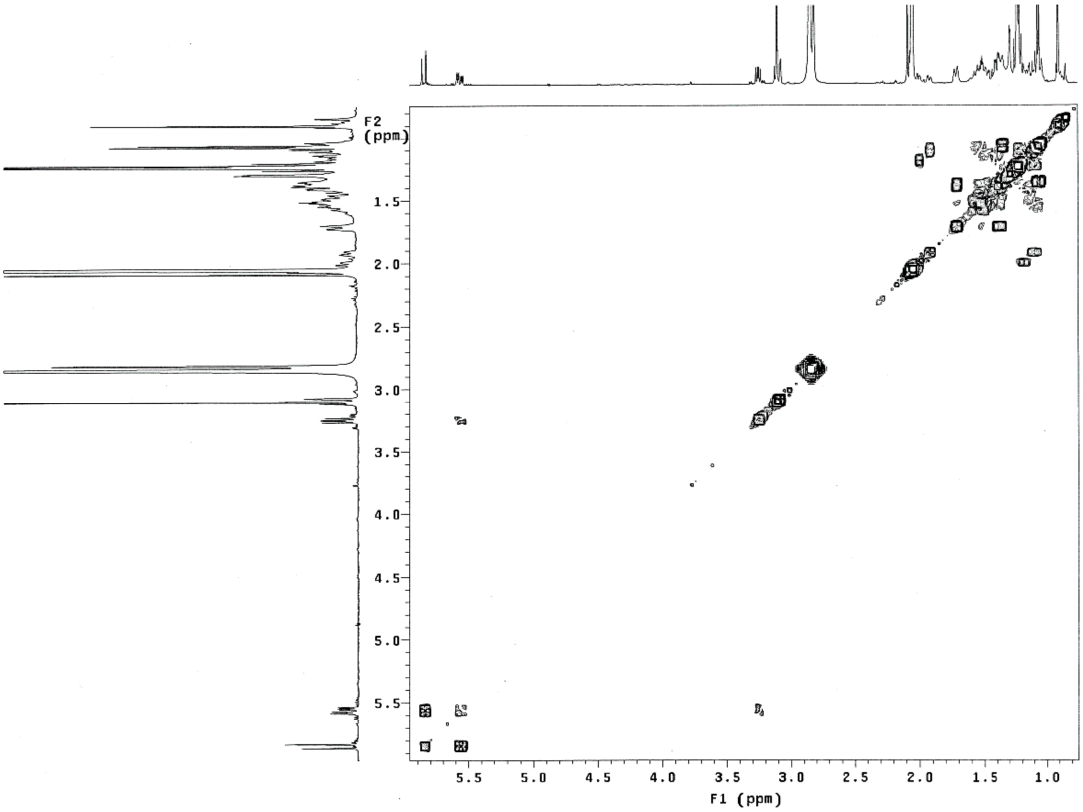

Figure S34. ^{1}H-^{1}H COSY spectrum of **5a** and **5b** in acetone-d_6.

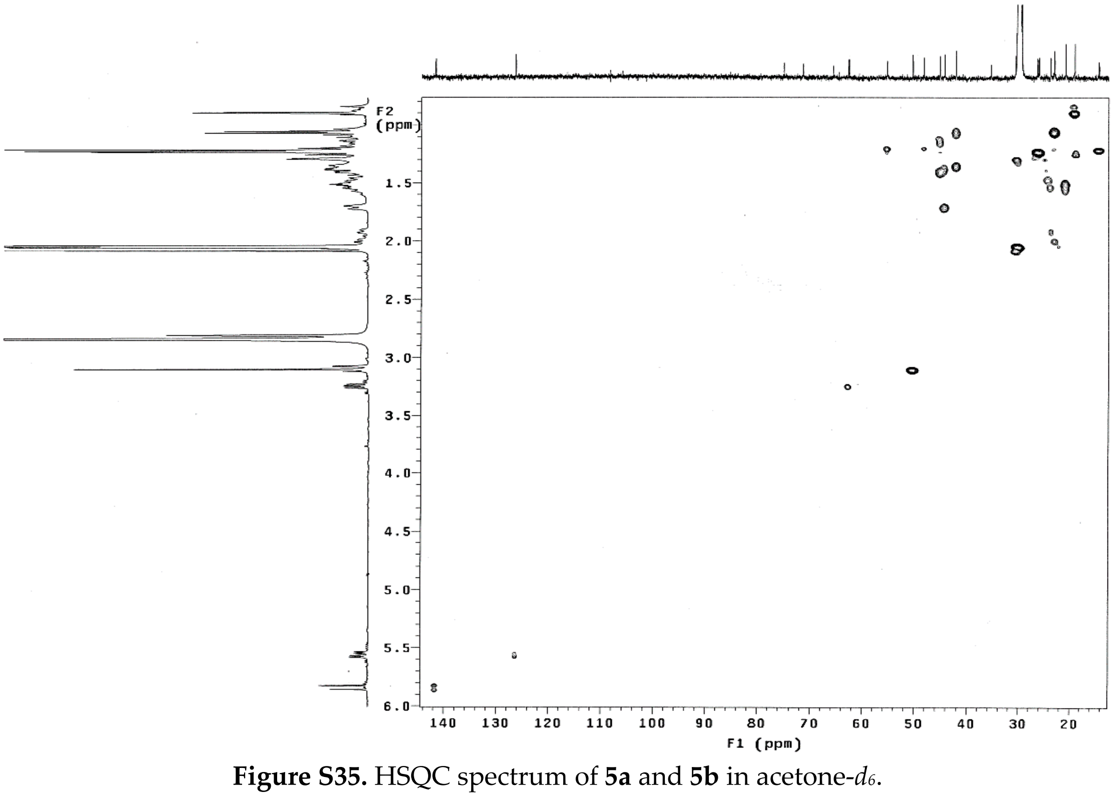

Figure S35. HSQC spectrum of **5a** and **5b** in acetone-d_6.

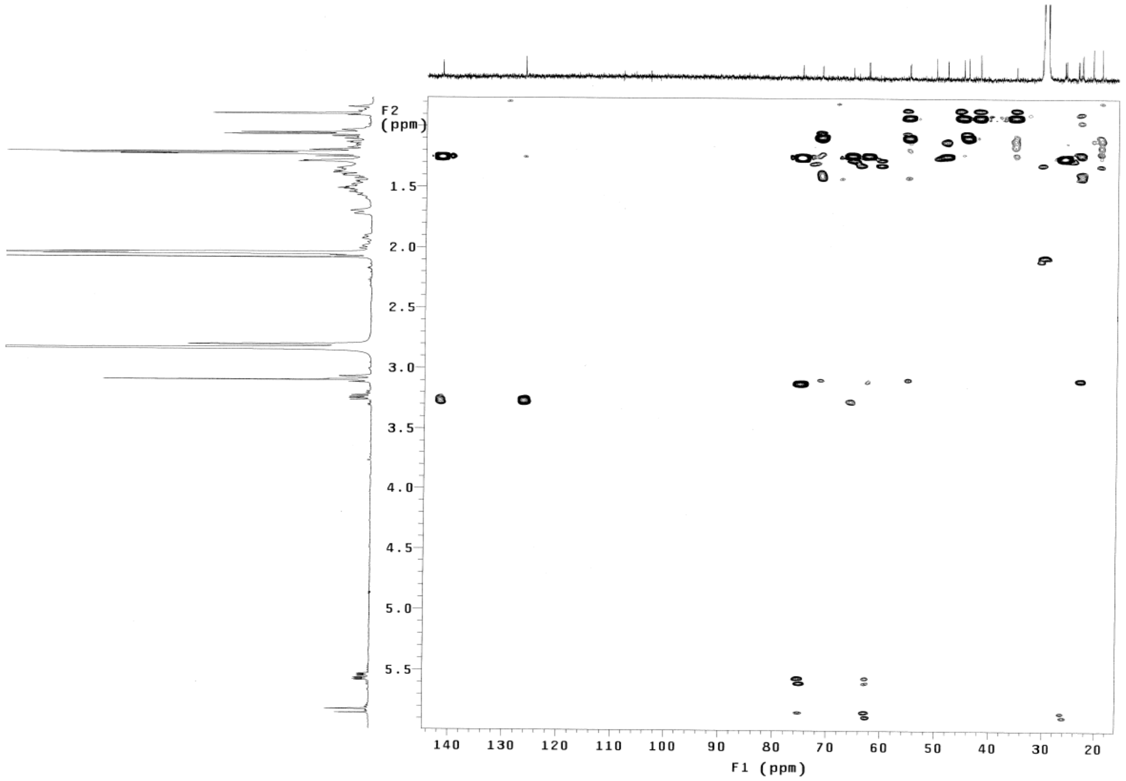

Figure S36. HMBC spectrum of **5a** and **5b** in acetone-d_6.

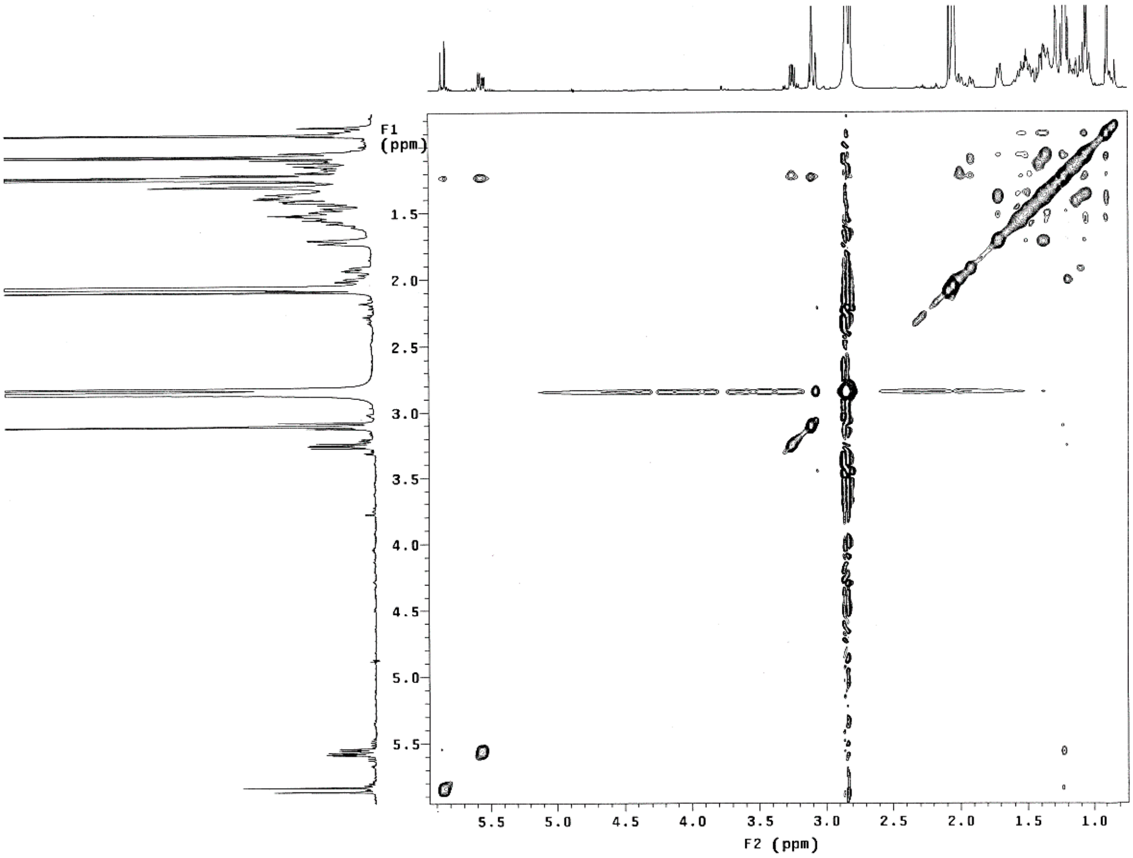

Figure S37. NOESY spectrum of **5a** and **5b** in acetone-d_6.

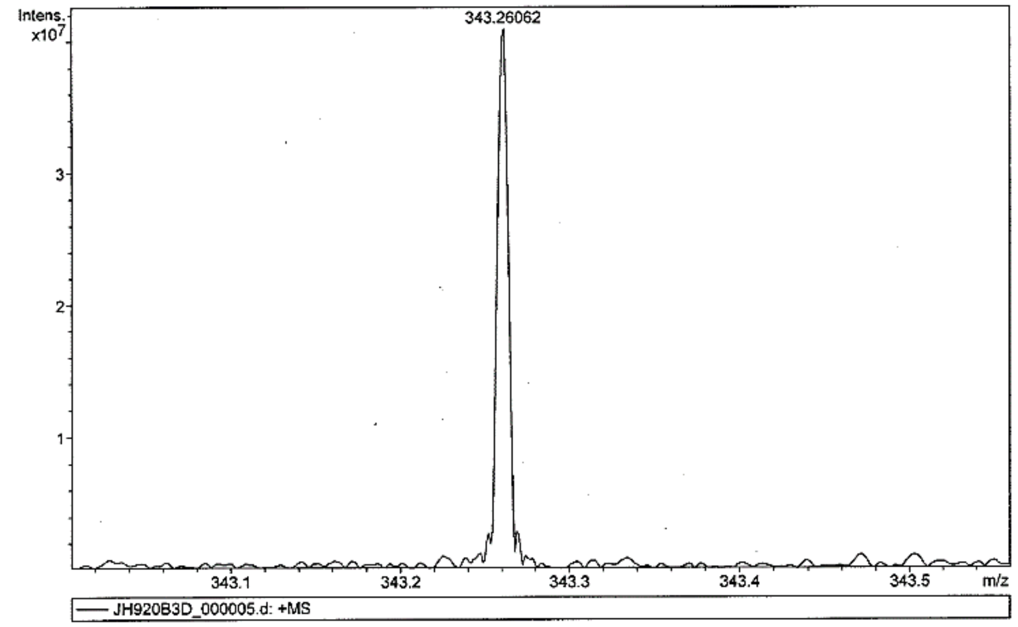

Meas. m/z	#	Formula	Score	m/z	err [mDa]	err [ppm]	mSigma	rdb	e⁻ Conf	N-Rule
343.26062	1	C 21 H 36 Na O 2	100.00	343.26075	0.13	0.39	15.3	3.5	even	ok

Figure S38. HRESIMS spectrum of **6**.

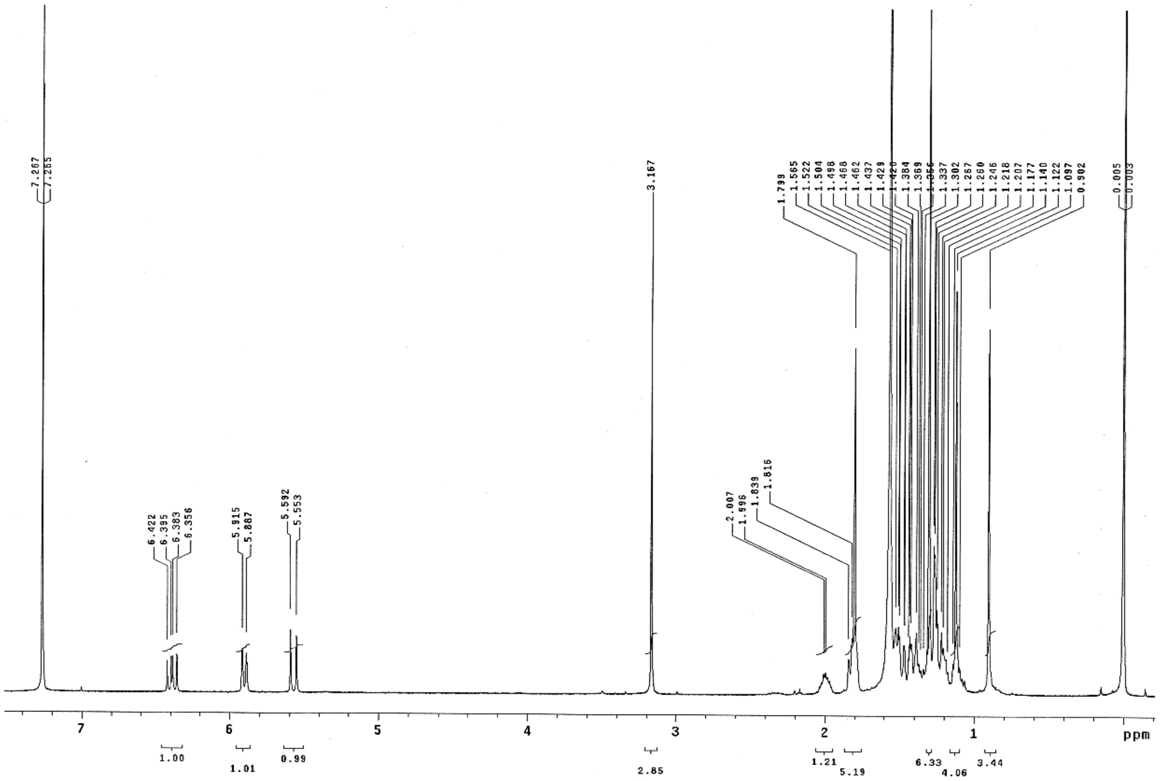

Figure S39. ^{1}H NMR spectrum of **6** in $CDCl_3$ at 400 MHz.

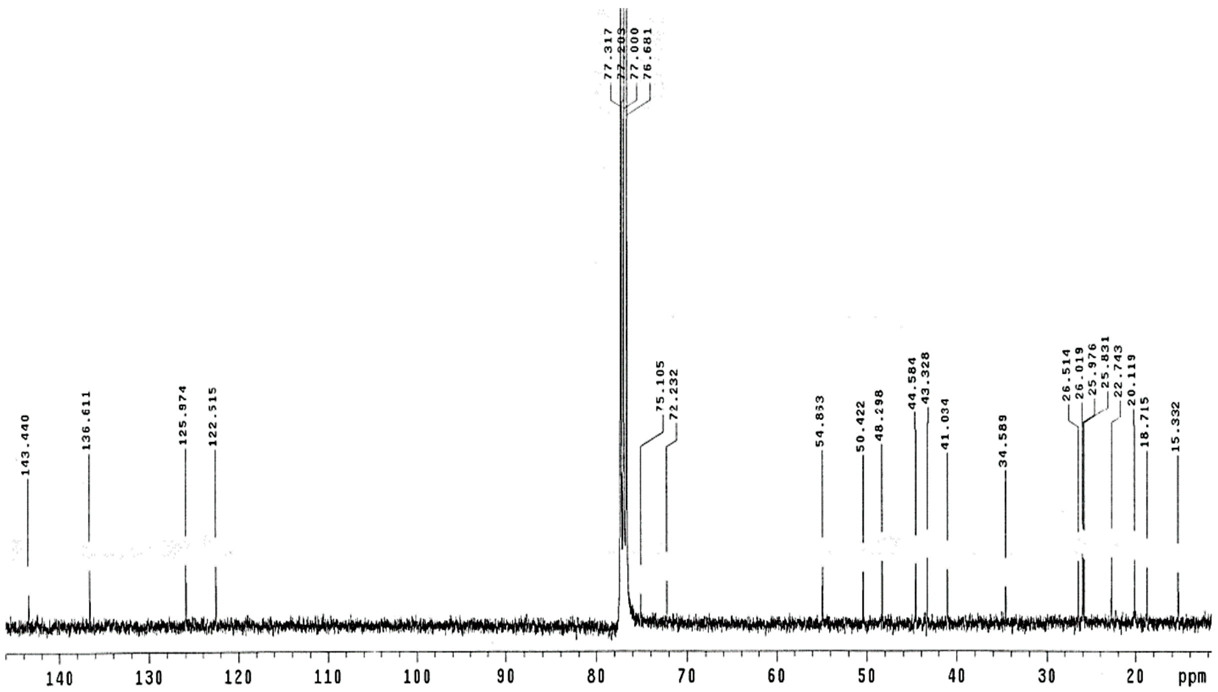

Figure S40. ^{13}C NMR spectrum of **6** in $CDCl_3$ at 100 MHz.

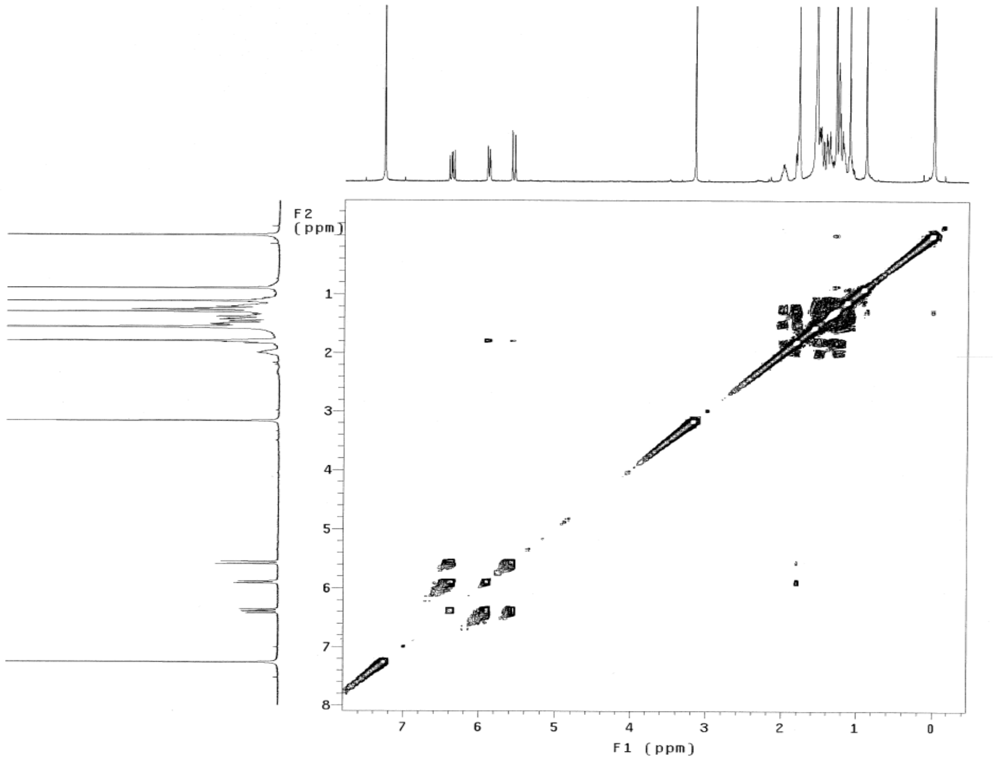

Figure S41. ^{1}H-^{1}H COSY spectrum of **6** in $CDCl_3$.

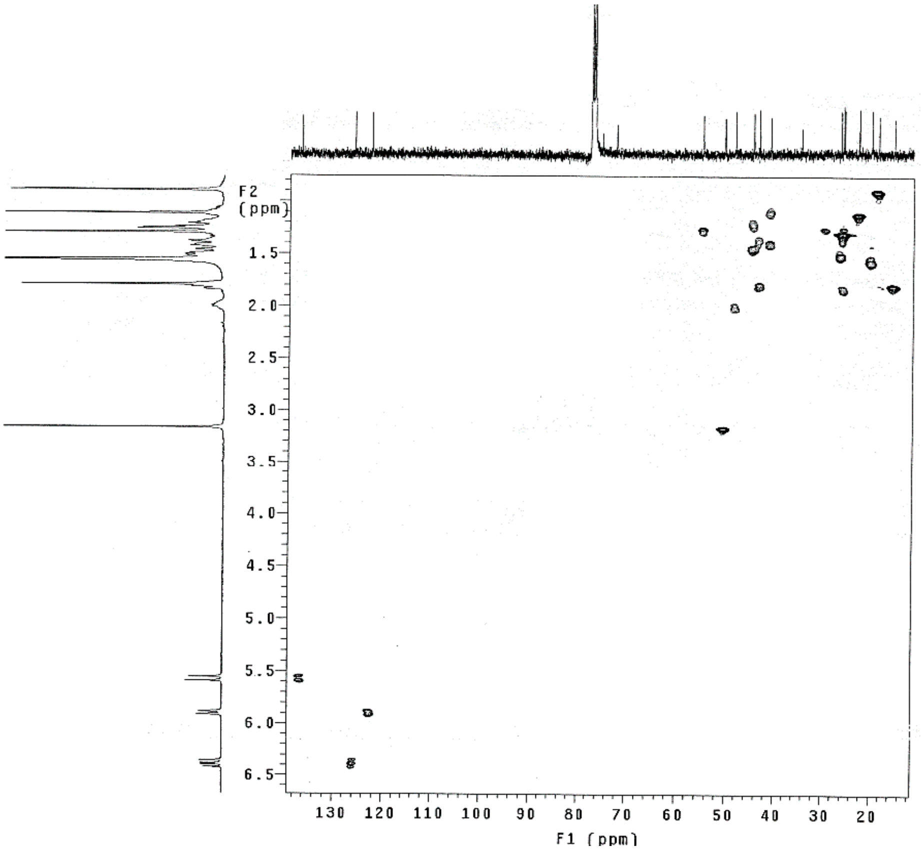

Figure S42. HSQC spectrum of **6** in $CDCl_3$.

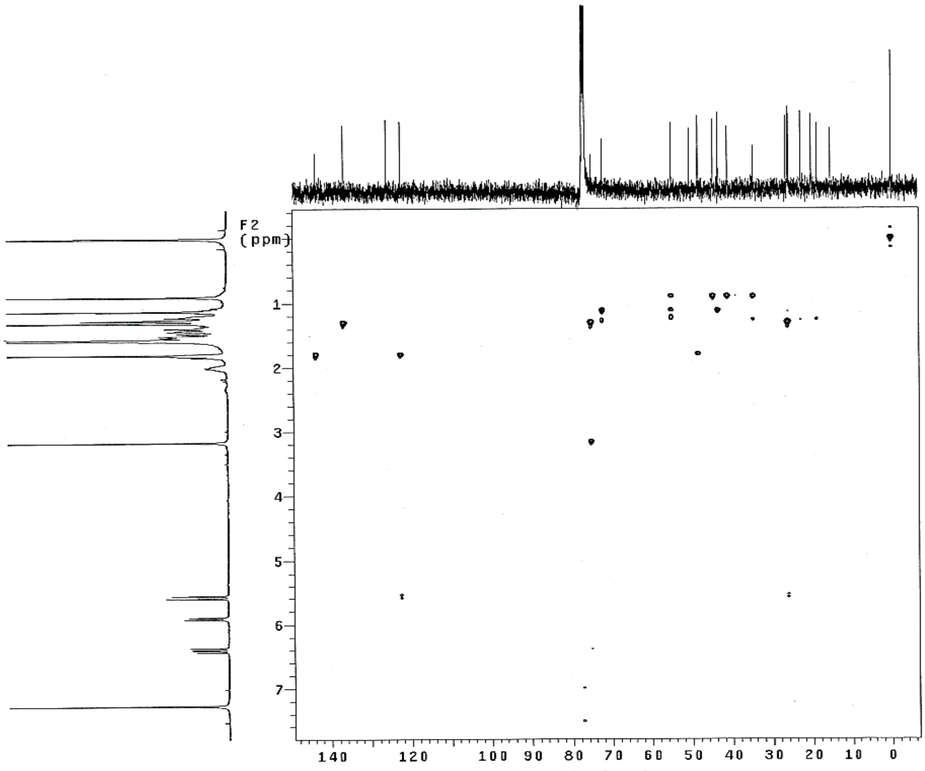

Figure S43. HMBC spectrum of **6** in $CDCl_3$.

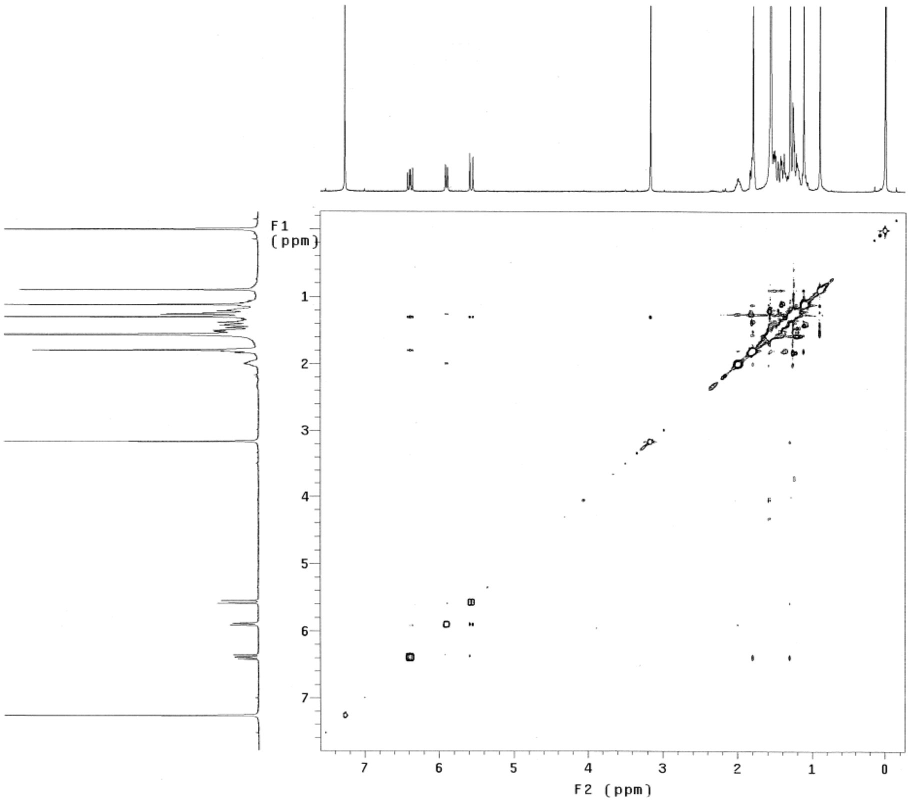

Figure S44. NOESY spectrum of **6** in $CDCl_3$.

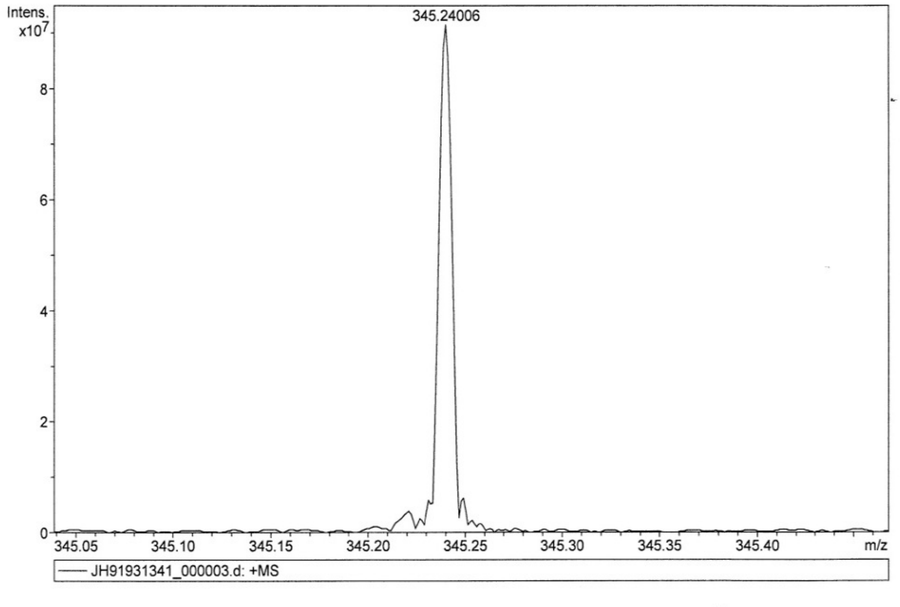

Meas. m/z	#	Formula	Score	m/z	err [mDa]	err [ppm]	mSigma	rdb	e⁻ Conf	N-Rule
345.24006	1	C 20 H 34 Na O 3	100.00	345.24002	-0.05	-0.14	4.3	3.5	even	ok

Figure S45. HRESIMS spectrum of **7**.

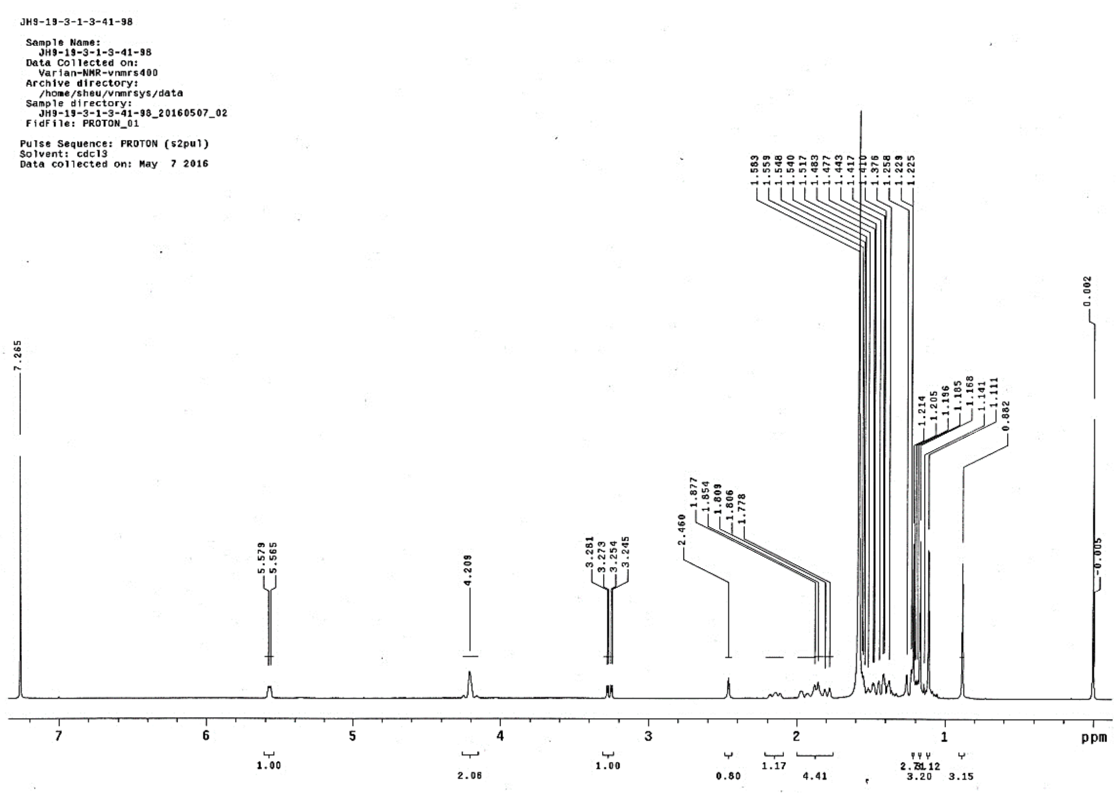

Figure S46. ^{1}H NMR spectrum of **7** in $CDCl_3$ at 400 MHz.

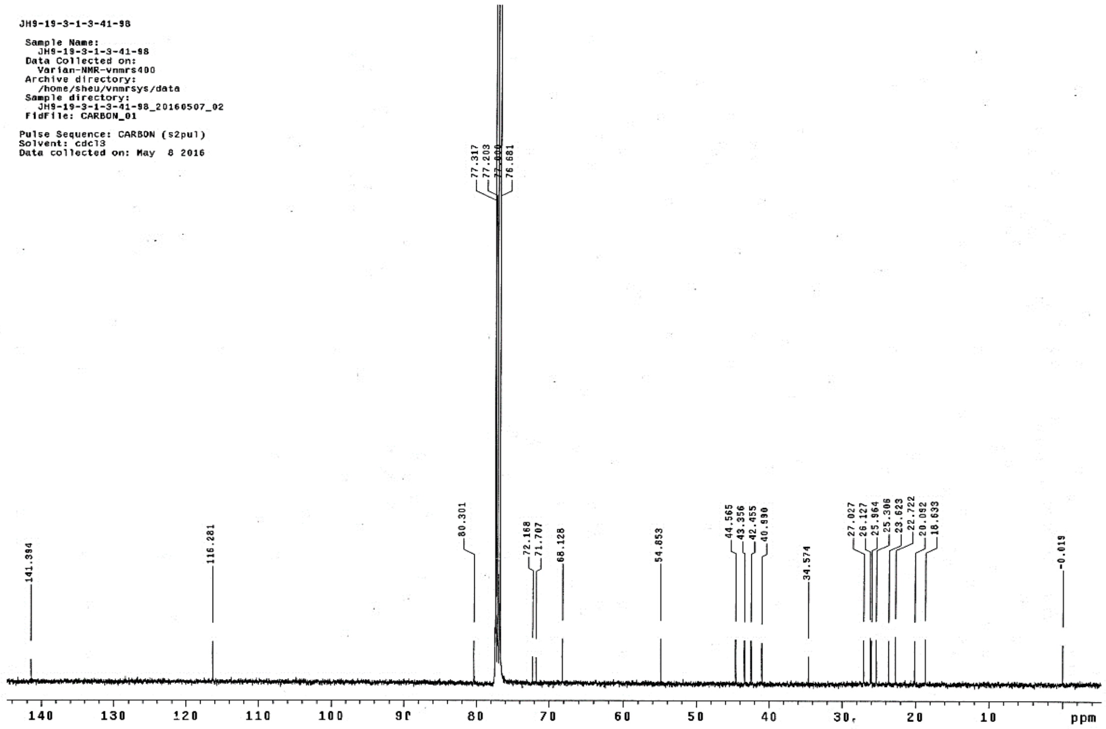

Figure S47. ^{13}C NMR spectrum of **7** in $CDCl_3$ at 100 MHz.

Figure S48. ^{1}H-^{1}H COSY spectrum of **7** in $CDCl_3$.

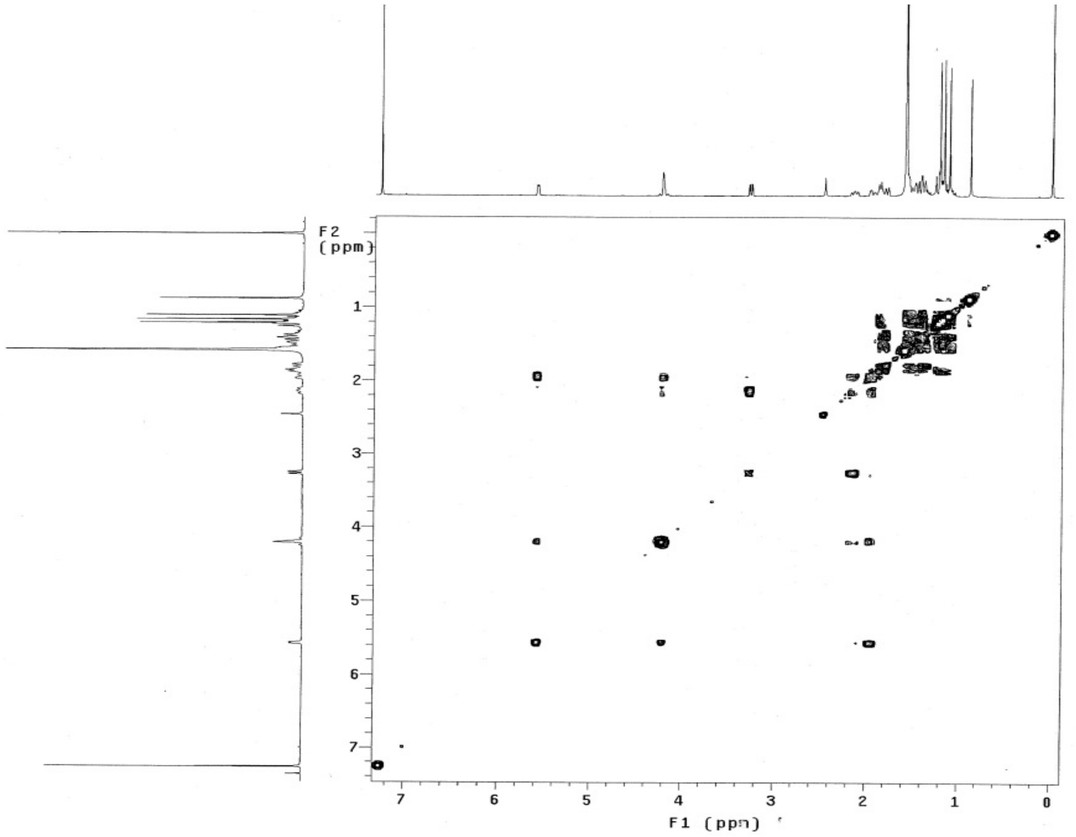

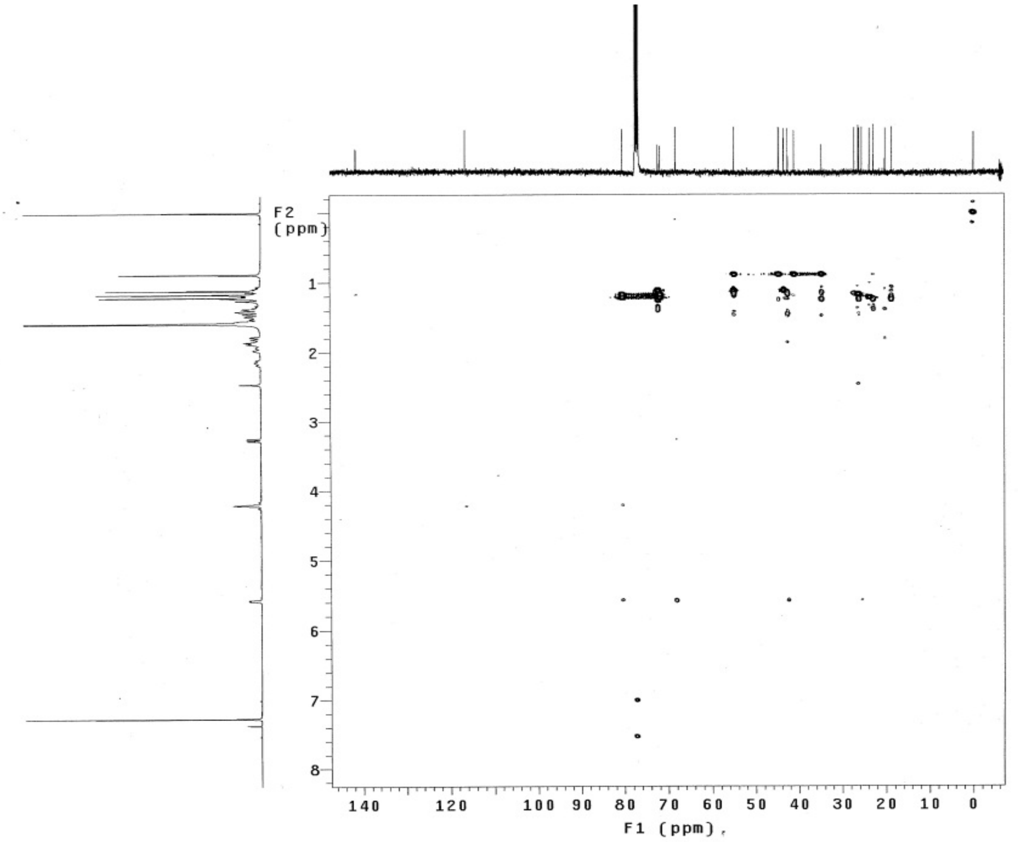

Figure S49. HSQC spectrum of **7** in $CDCl_3$.

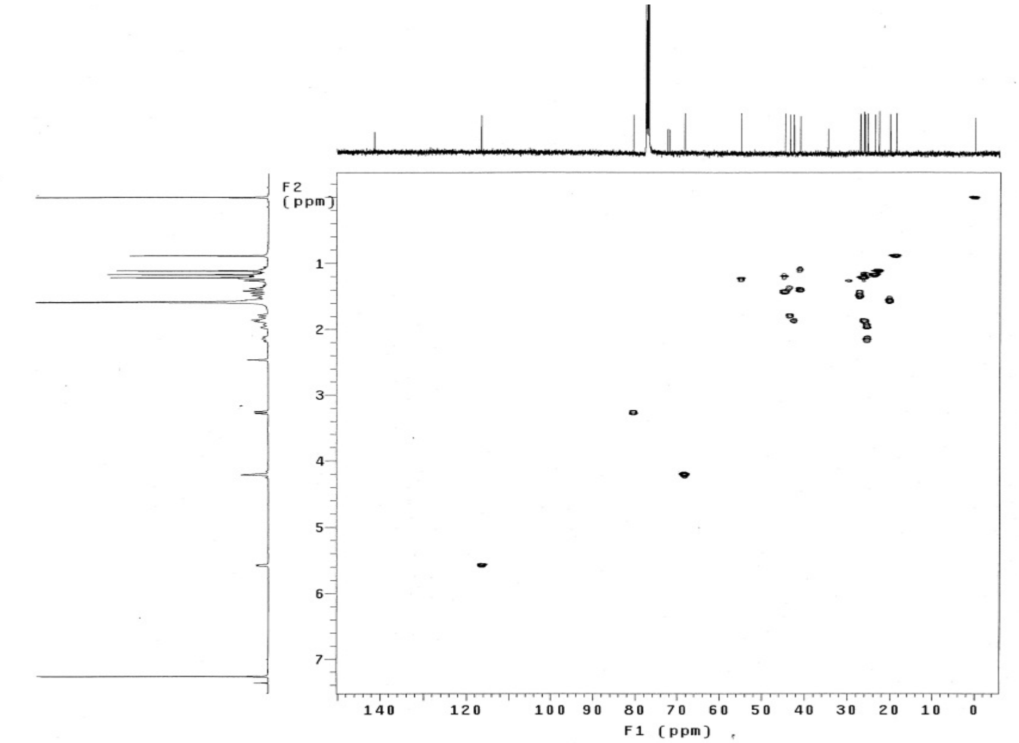

Figure S50. HMBC spectrum of **7** in $CDCl_3$.

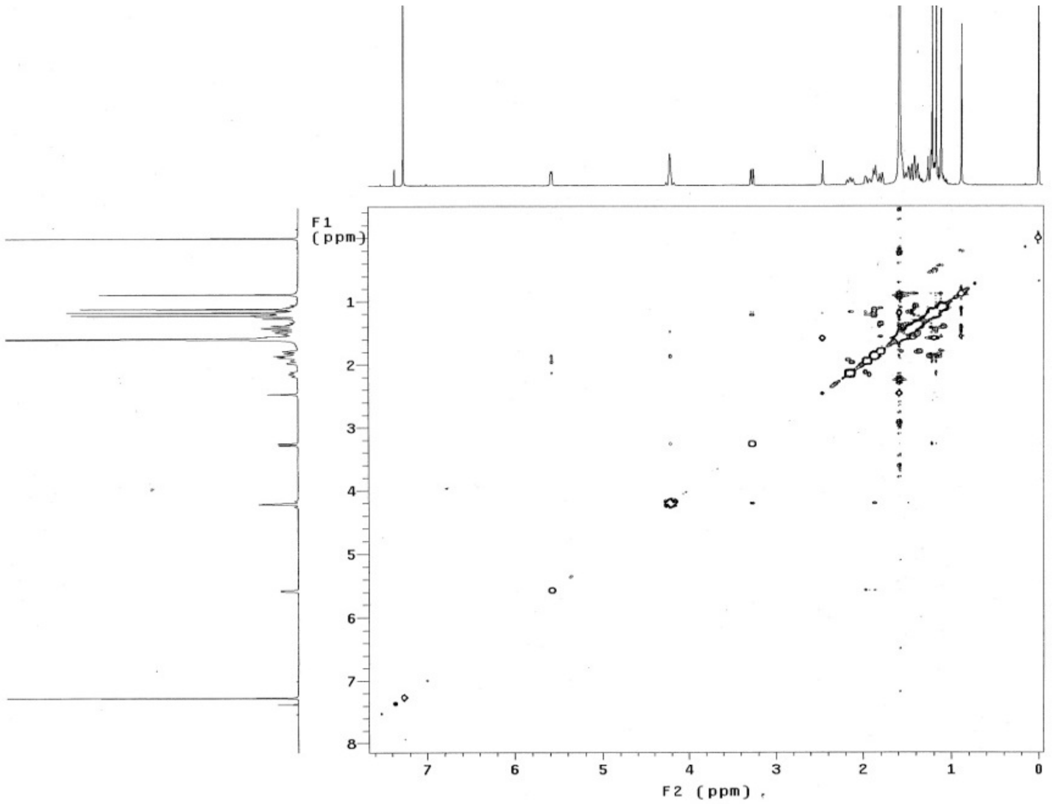

Figure S51. NOESY spectrum of **7** in $CDCl_3$.

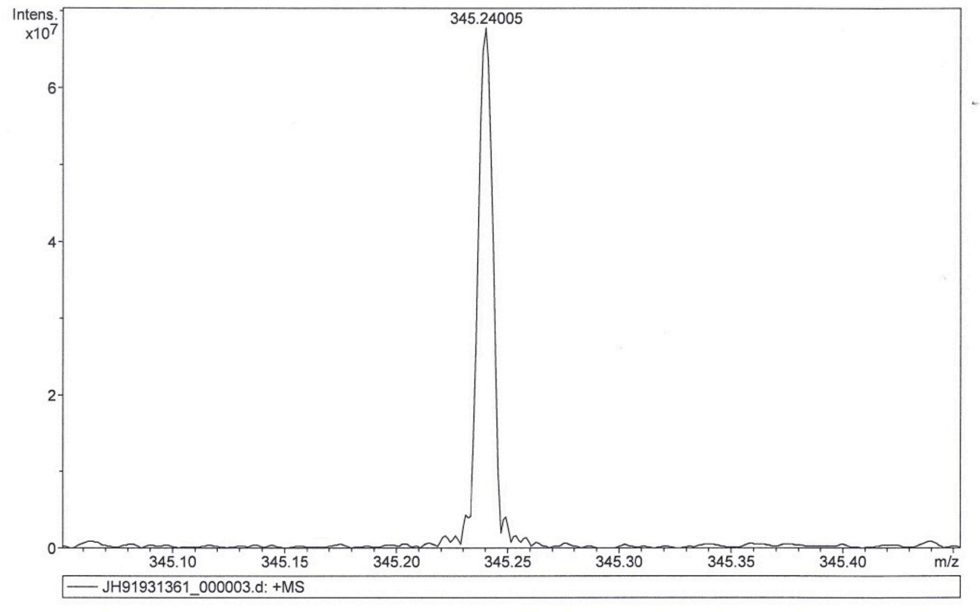

Meas. m/z	#	Formula	Score	m/z	err [mDa]	err [ppm]	mSigma	rdb	e¯ Conf	N-Rule
345.24005	1	C 20 H 34 Na O 3	100.00	345.24002	-0.04	-0.11	13.3	3.5	even	ok

Figure S52. HRESIMS spectrum of **8**.

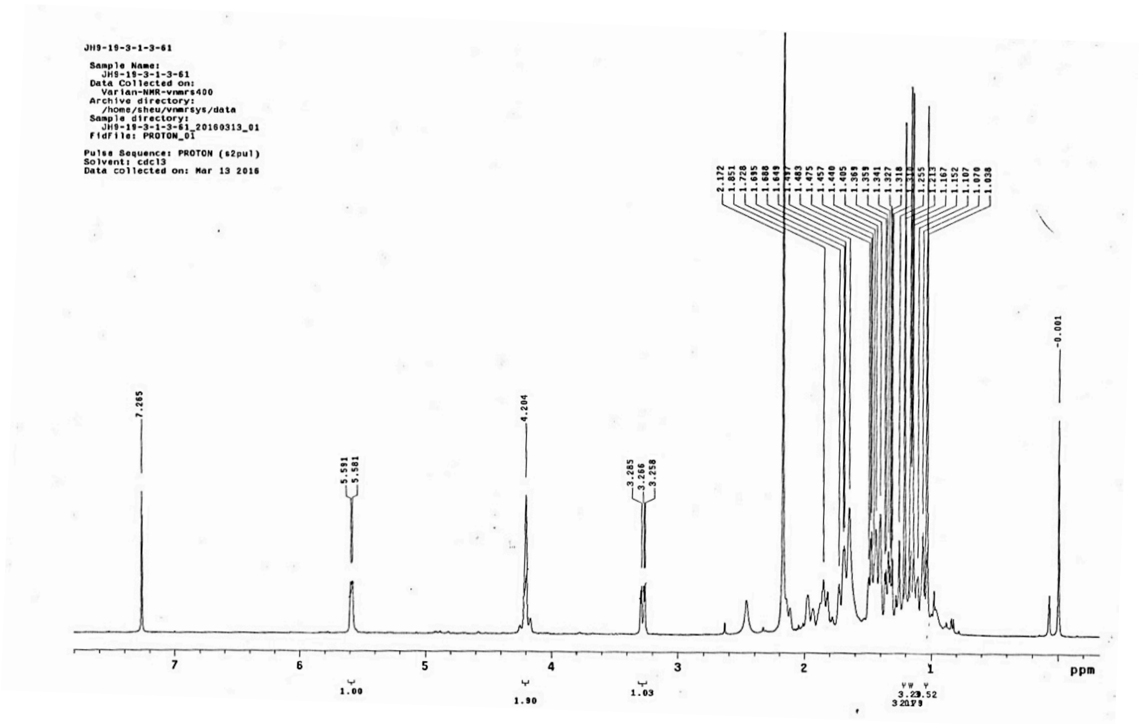

Figure S53. ^{1}H NMR spectrum of **8** in $CDCl_3$ at 400 MHz.

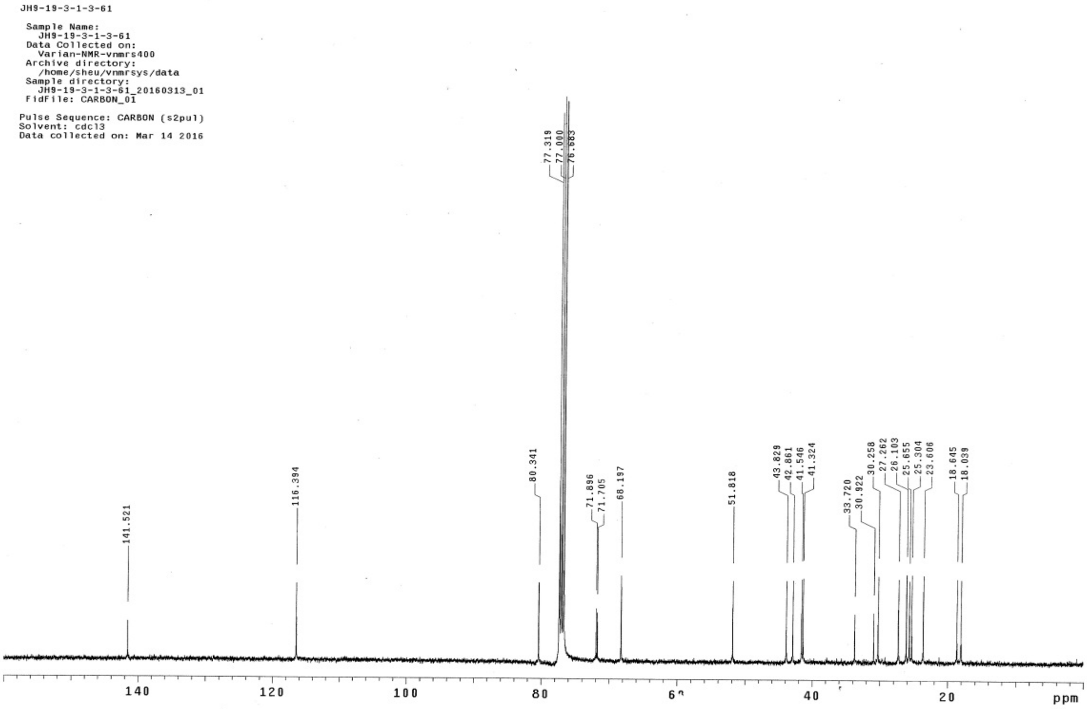

Figure S54. ^{13}C NMR spectrum of **8** in $CDCl_3$ at 100 MHz.

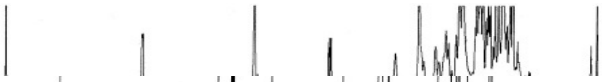

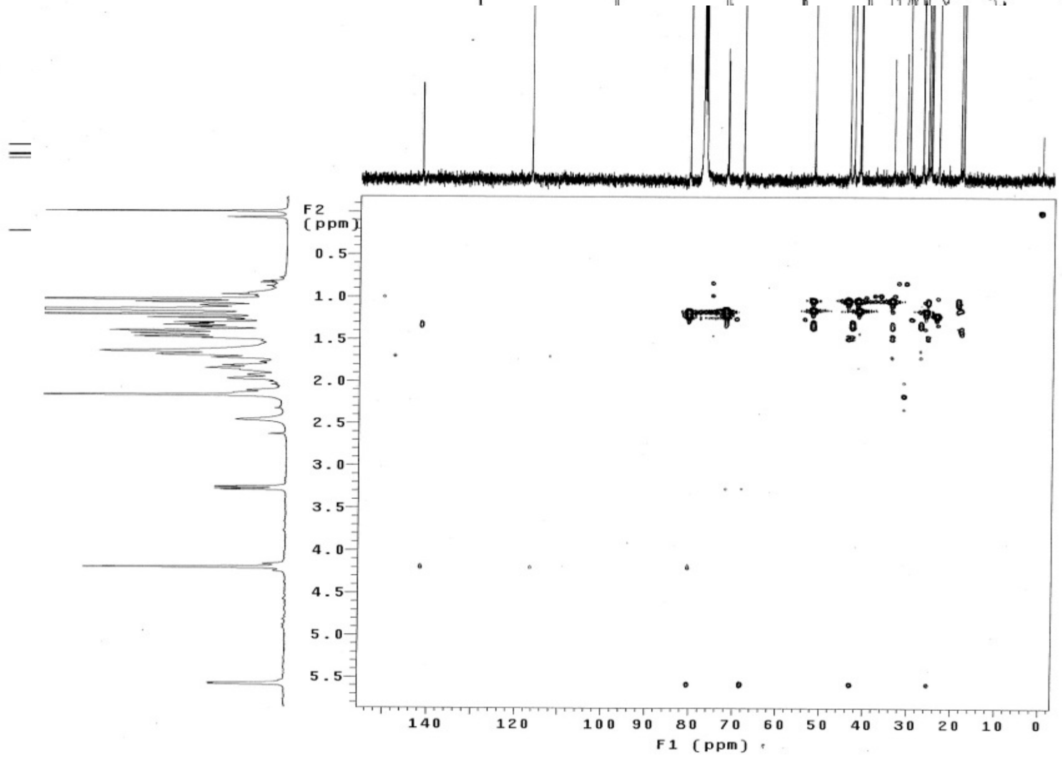

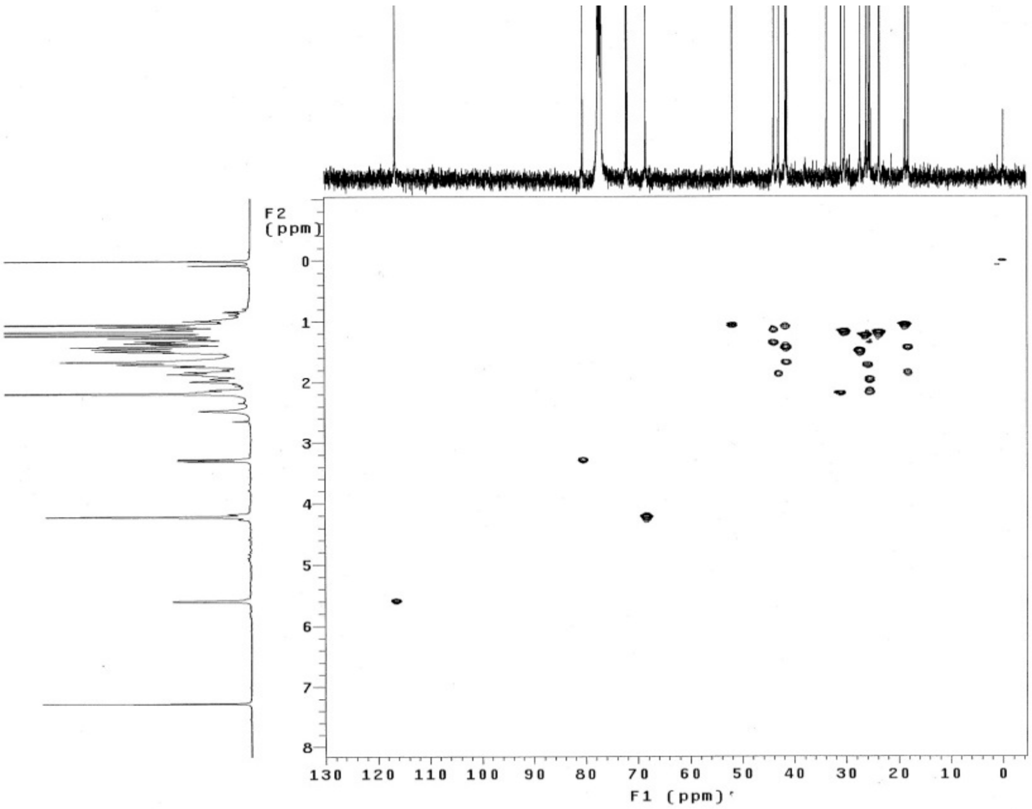

Figure S55. ¹H-¹H COSY spectrum of **8** in $CDCl_3$.

Figure S56. HSQC spectrum of **8** in $CDCl_3$.

Figure S57. HMBC spectrum of **8** in $CDCl_3$.

Figure S58. NOESY spectrum of **8** in $CDCl_3$.

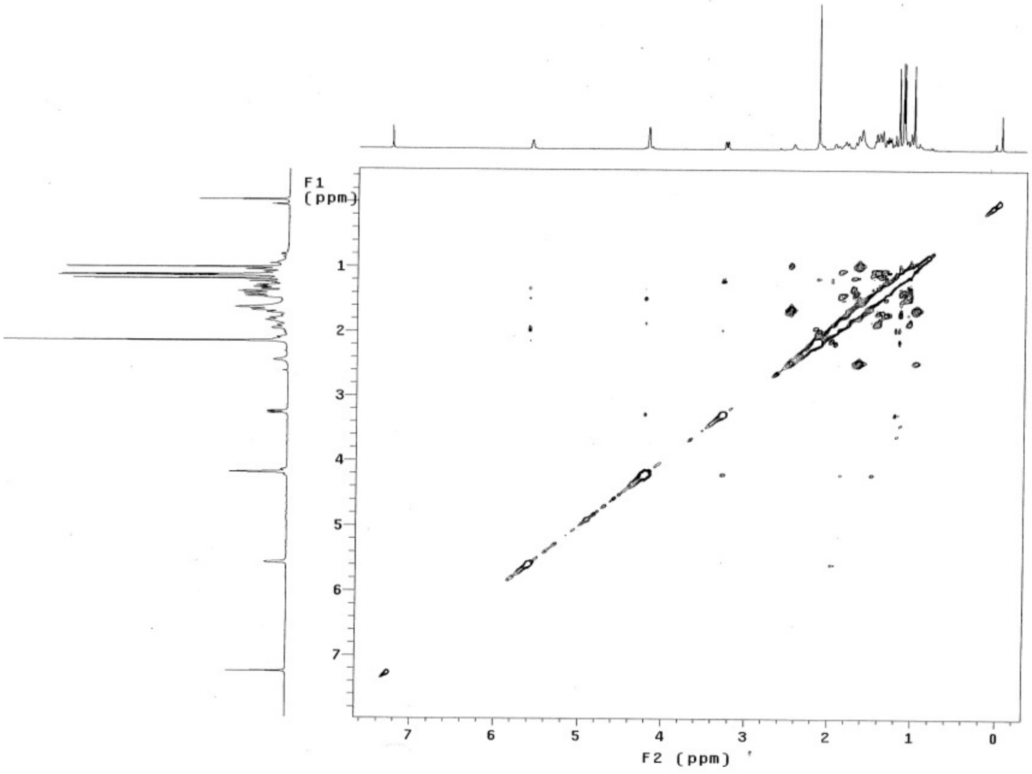

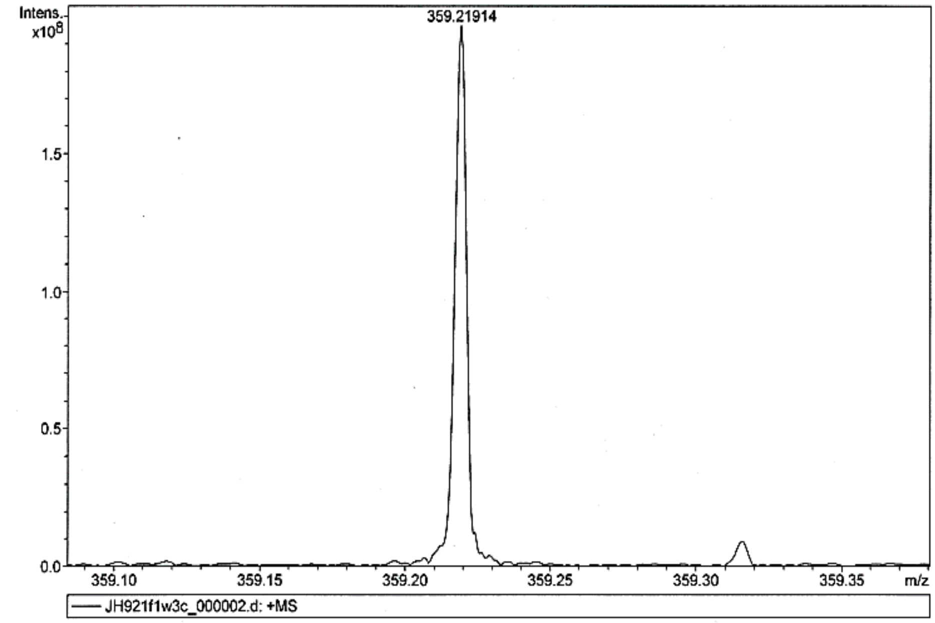

Meas. m/z	#	Formula	Score	m/z	err [mDa]	err [ppm]	mSigma	rdb	e⁻ Conf	N-Rule
359.21914	1	C 20 H 32 Na O 4	100.00	359.21928	0.14	0.40	9.4	4.5	even	ok

Figure S59. HRESIMS spectrum of **9**.

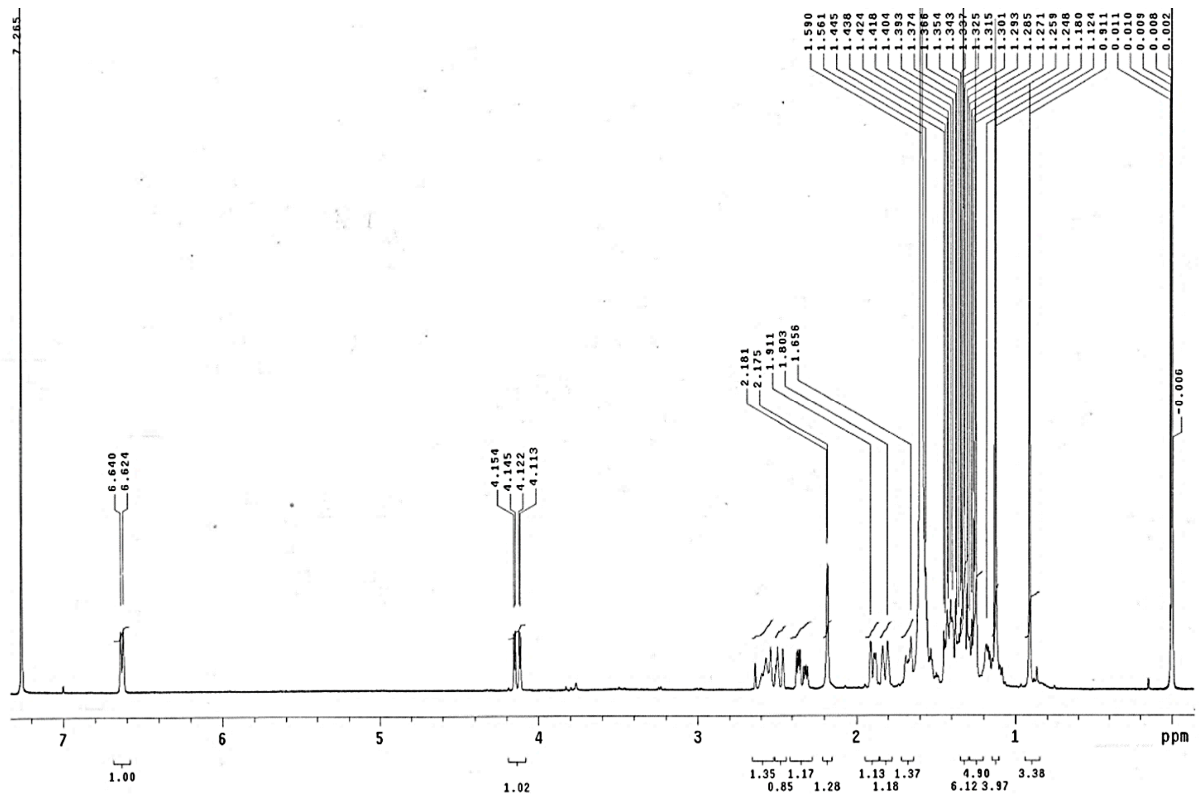

Figure S60. ^{1}H NMR spectrum of **9** in $CDCl_3$ at 400 MHz.

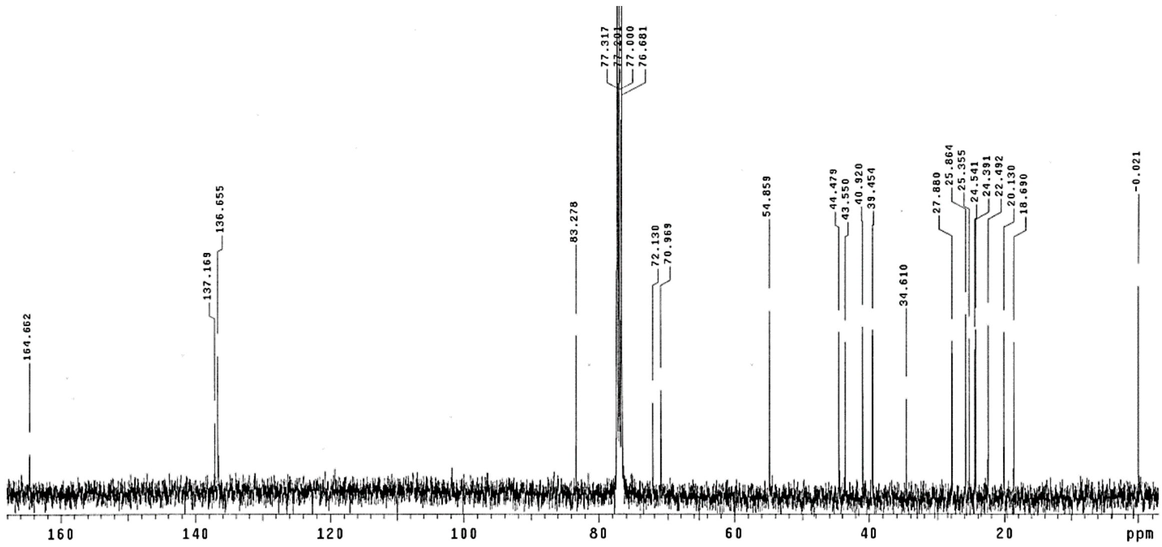

Figure S61. ^{13}C NMR spectrum of **9** in $CDCl_3$ at 100 MHz.

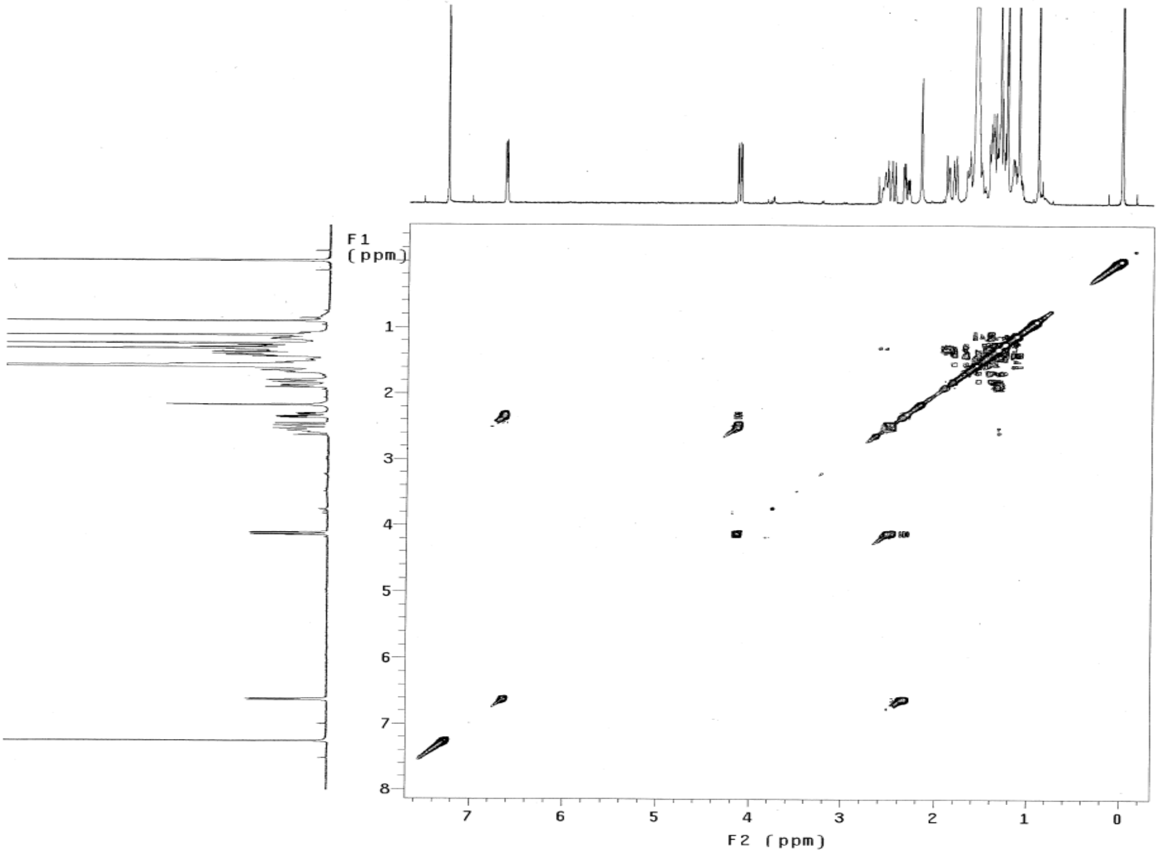

Figure S62. ^{1}H-^{1}H COSY spectrum of **9** in $CDCl_3$.

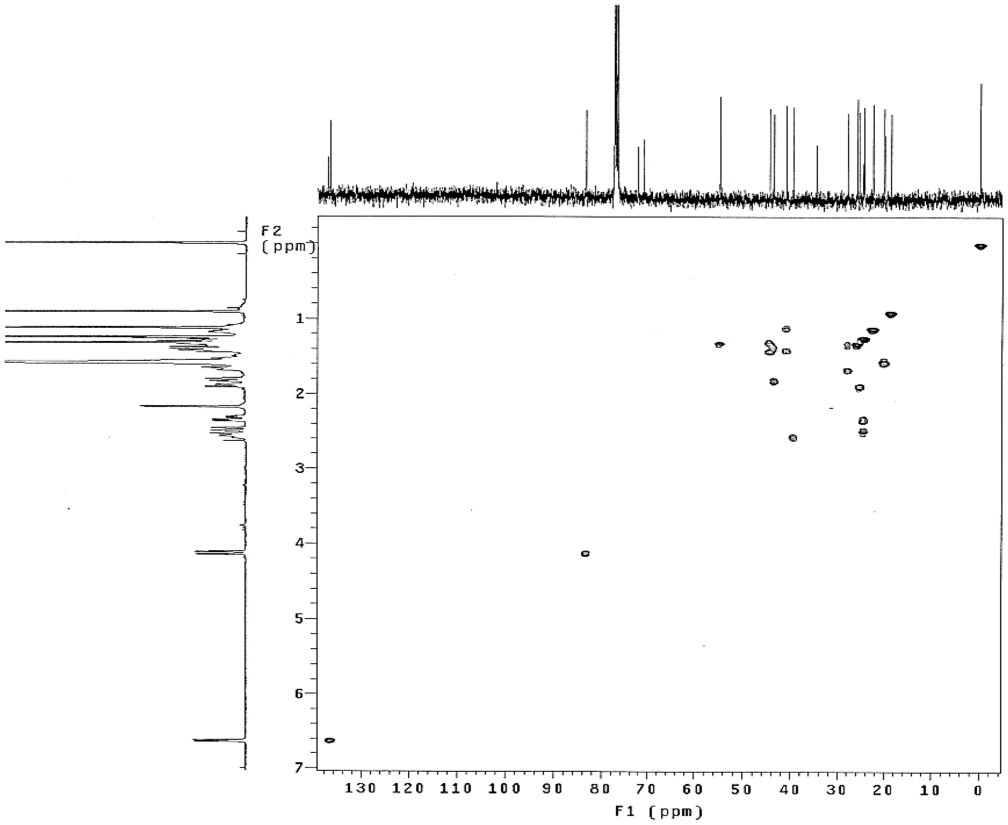

Figure S63. HSQC spectrum of **9** in $CDCl_3$.

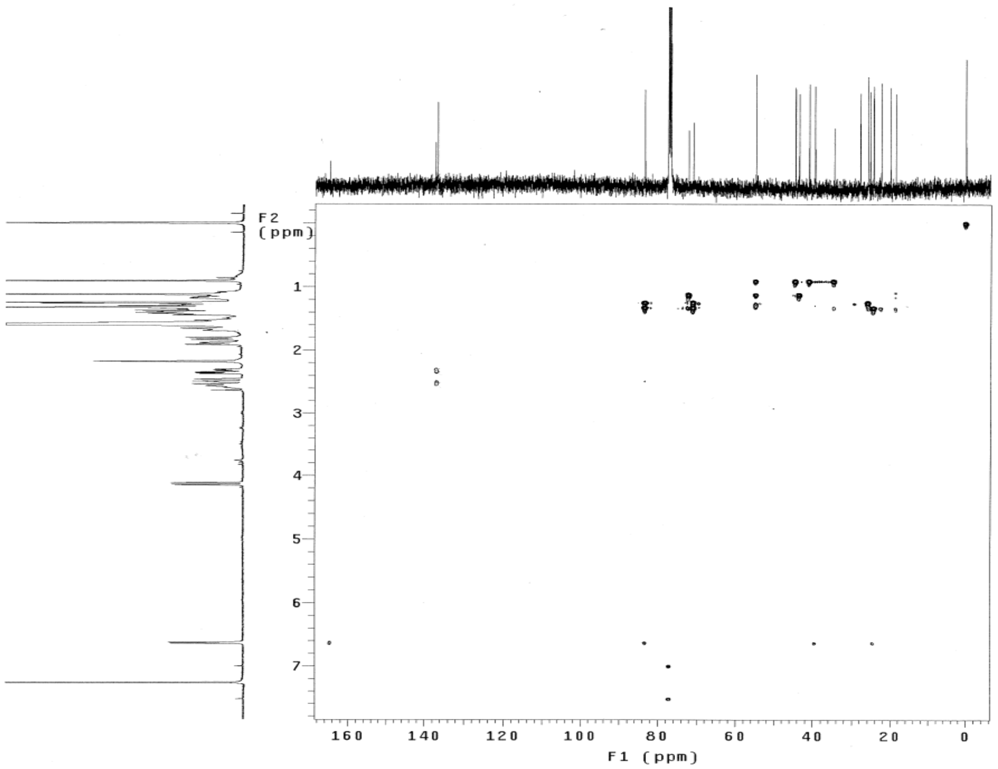

Figure S64. HMBC spectrum of **9** in $CDCl_3$.

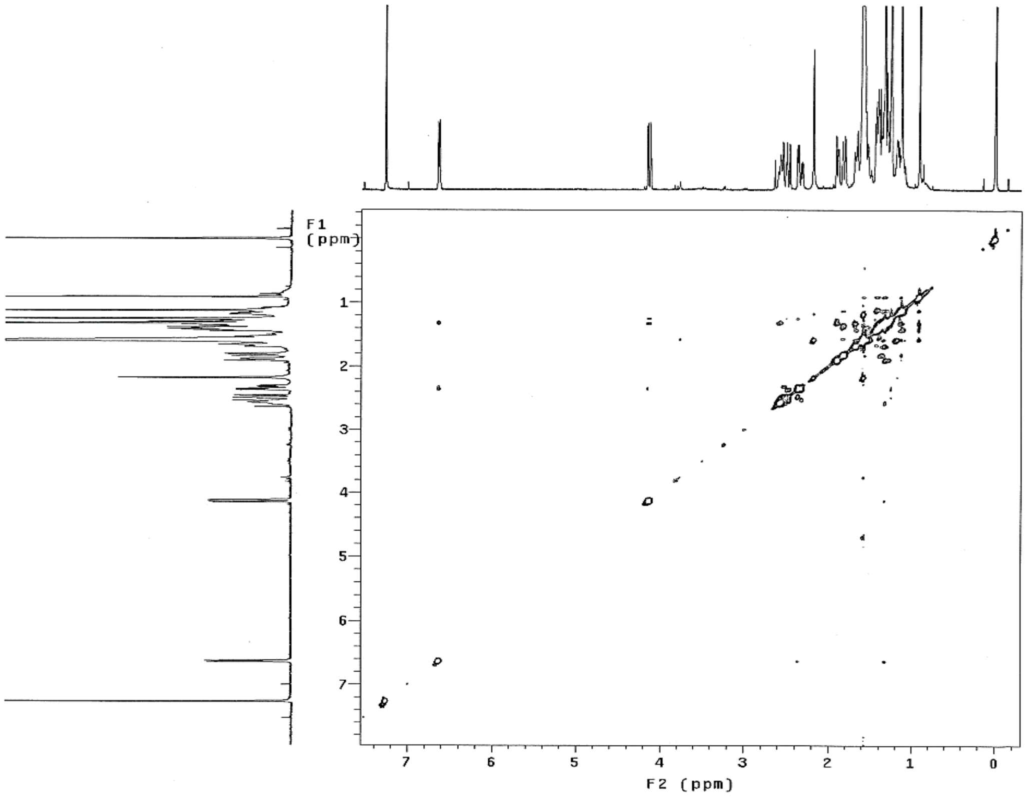

Figure S65. NOESY spectrum of **9** in $CDCl_3$.

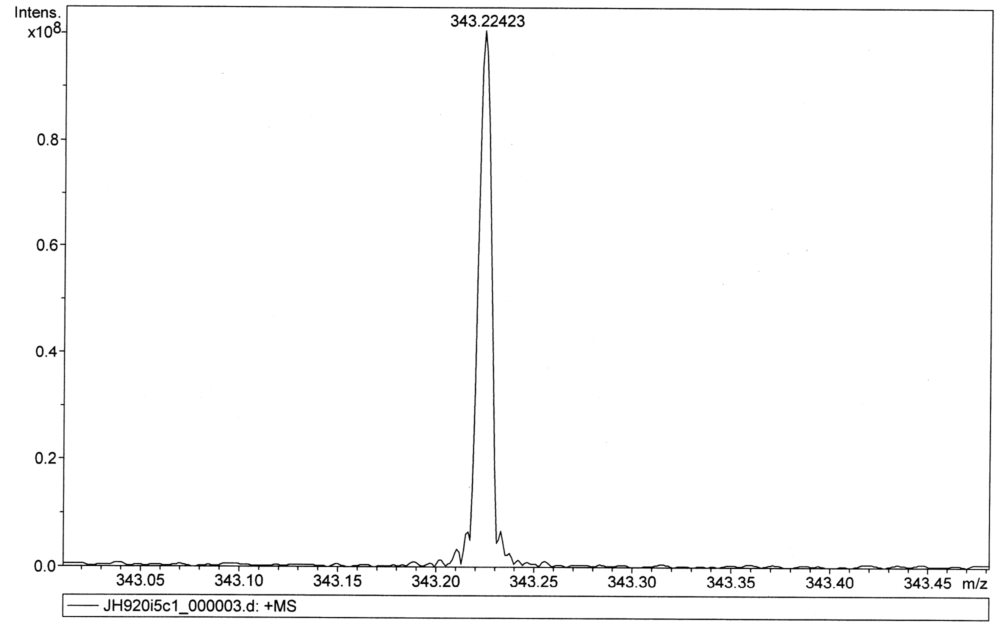

Meas. m/z	#	Formula	Score	m/z	err [mDa]	err [ppm]	mSigma	rdb	e⁻ Conf	N-Rule
343.22423	1	C 20 H 32 Na O 3	100.00	343.22437	0.13	0.39	17.0	4.5	even	ok

Figure S66. HRESIMS spectrum of **10**.

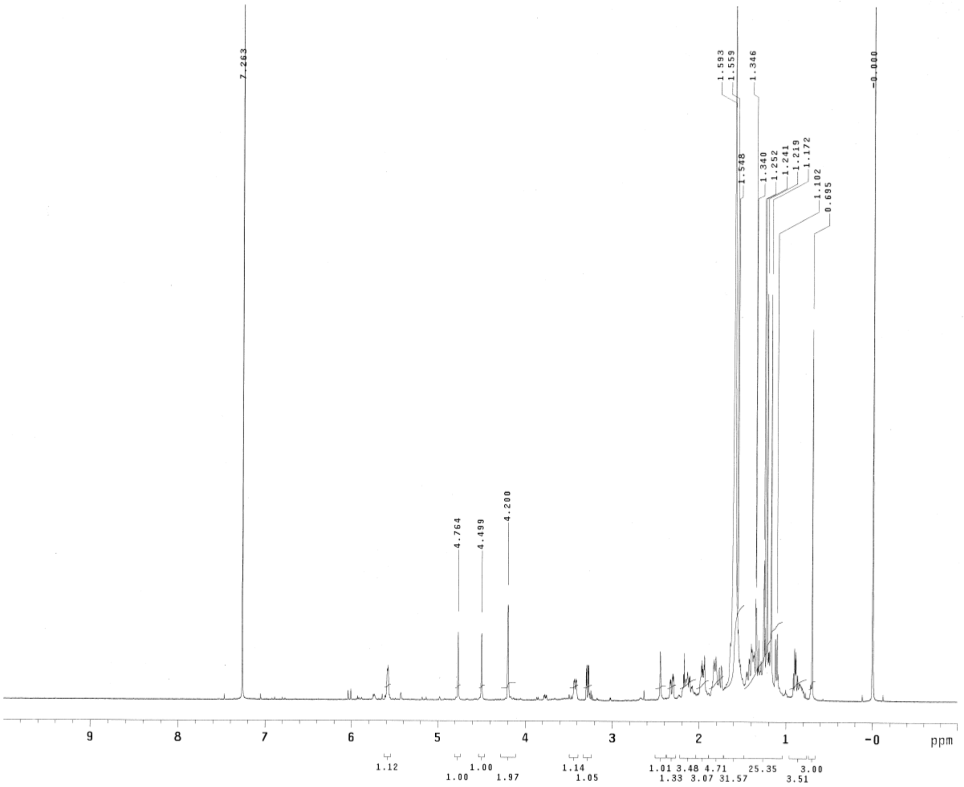

Figure S67. ^{1}H NMR spectrum of **10** in $CDCl_3$ at 500 MHz.

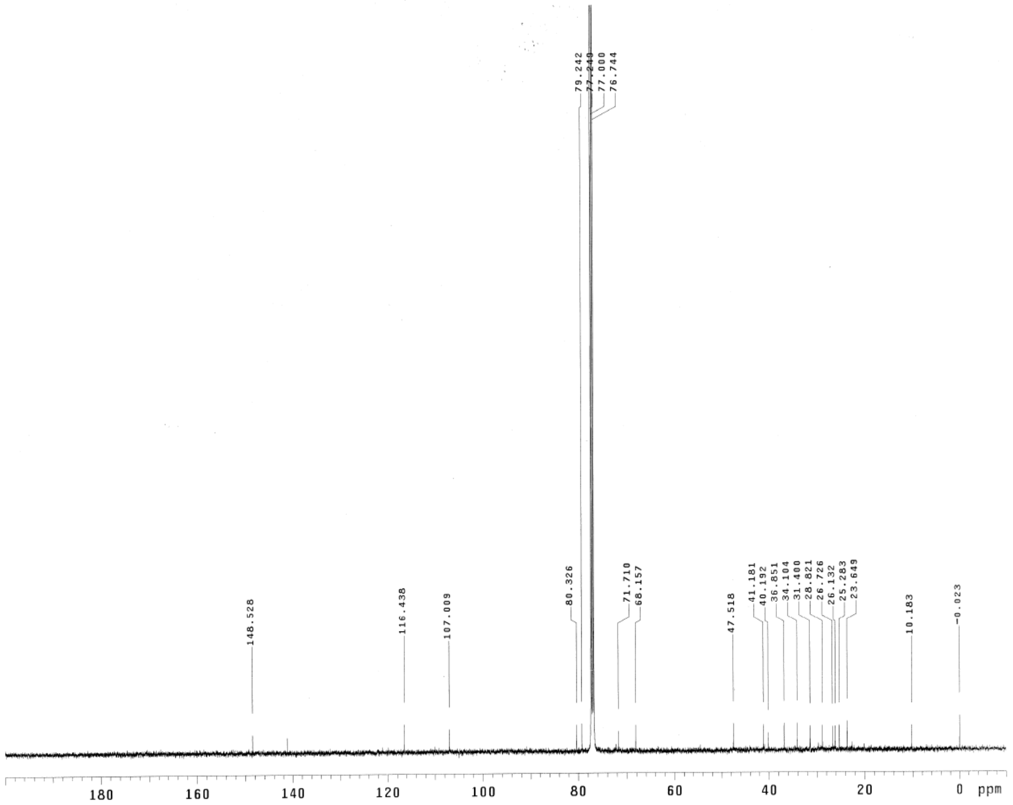

Figure S68. ^{13}C NMR spectrum of **10** in $CDCl_3$ at 125 MHz.

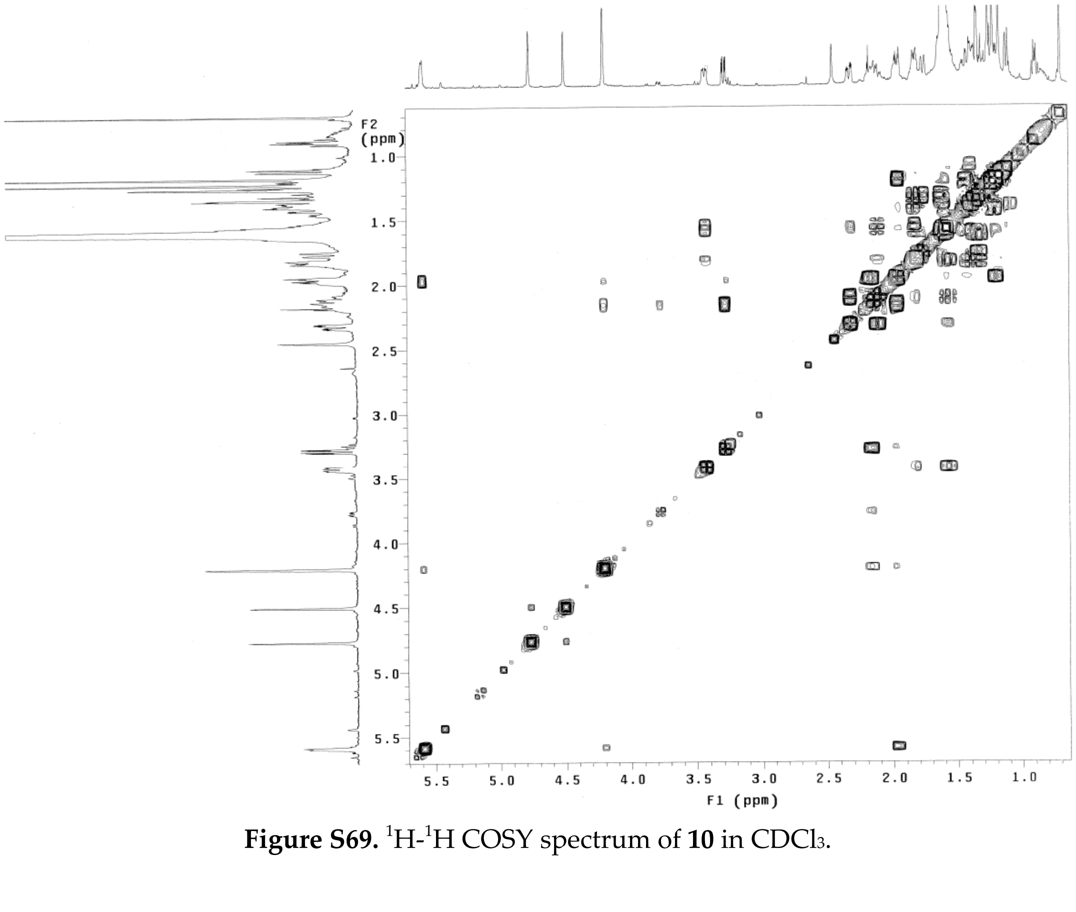

Figure S69. ^{1}H-^{1}H COSY spectrum of **10** in $CDCl_3$.

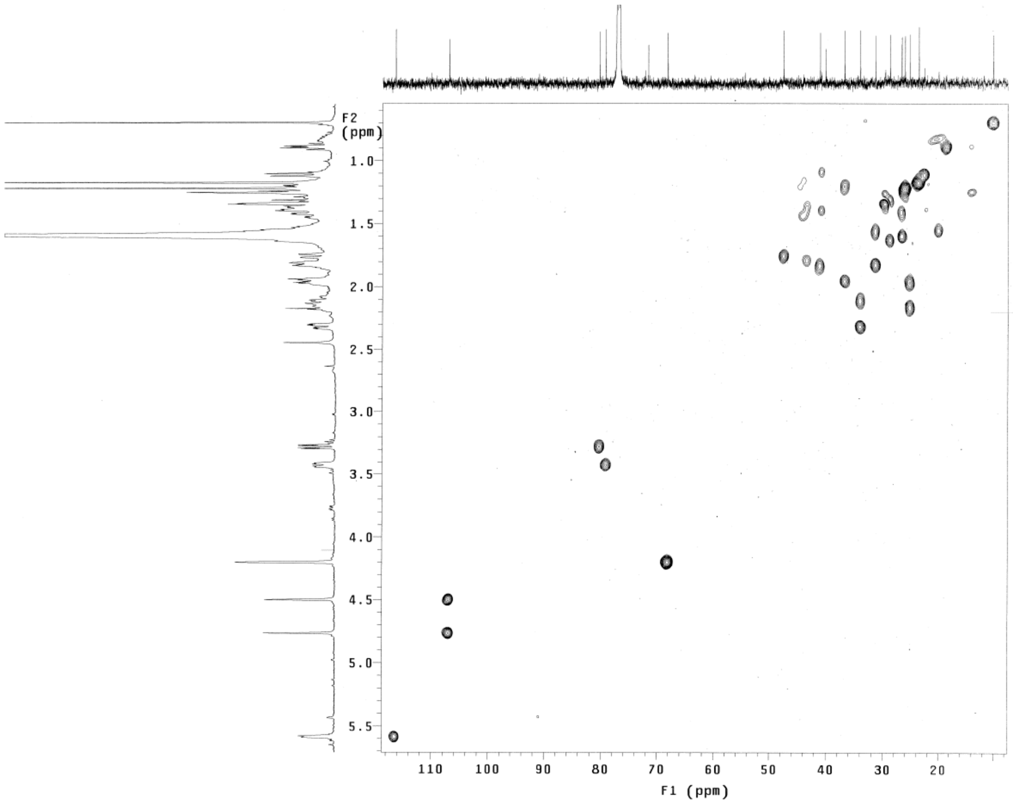

Figure S70. HSQC spectrum of **10** in $CDCl_3$.

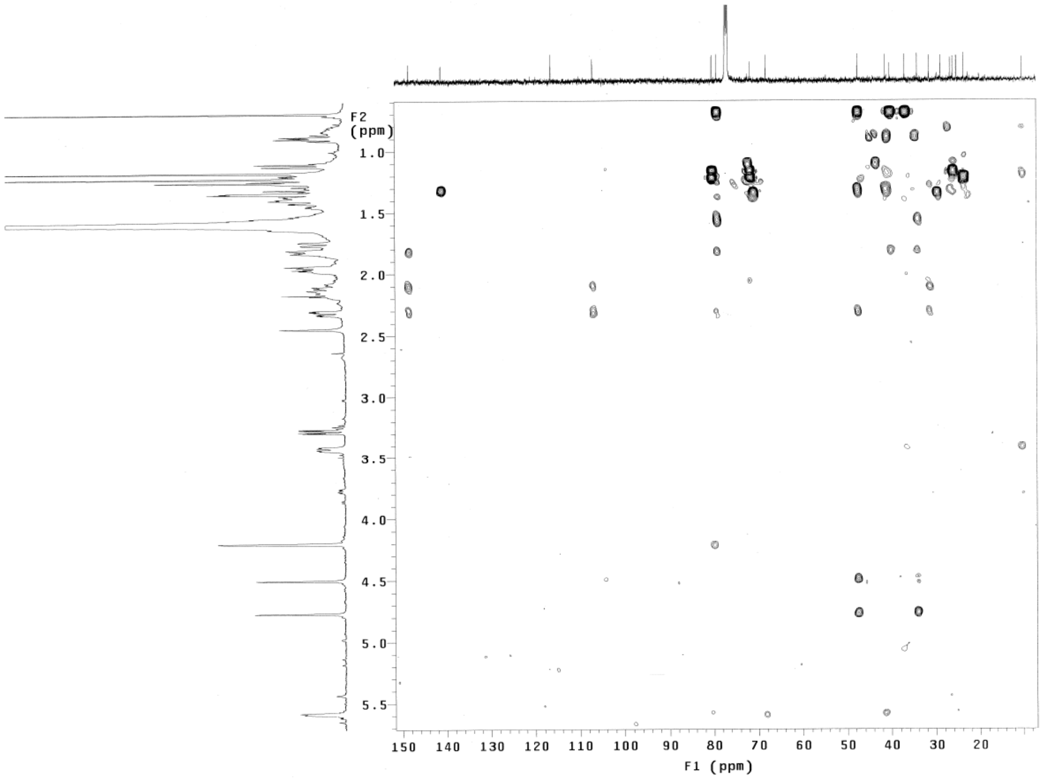

Figure S71. HMBC spectrum of **10** in $CDCl_3$.

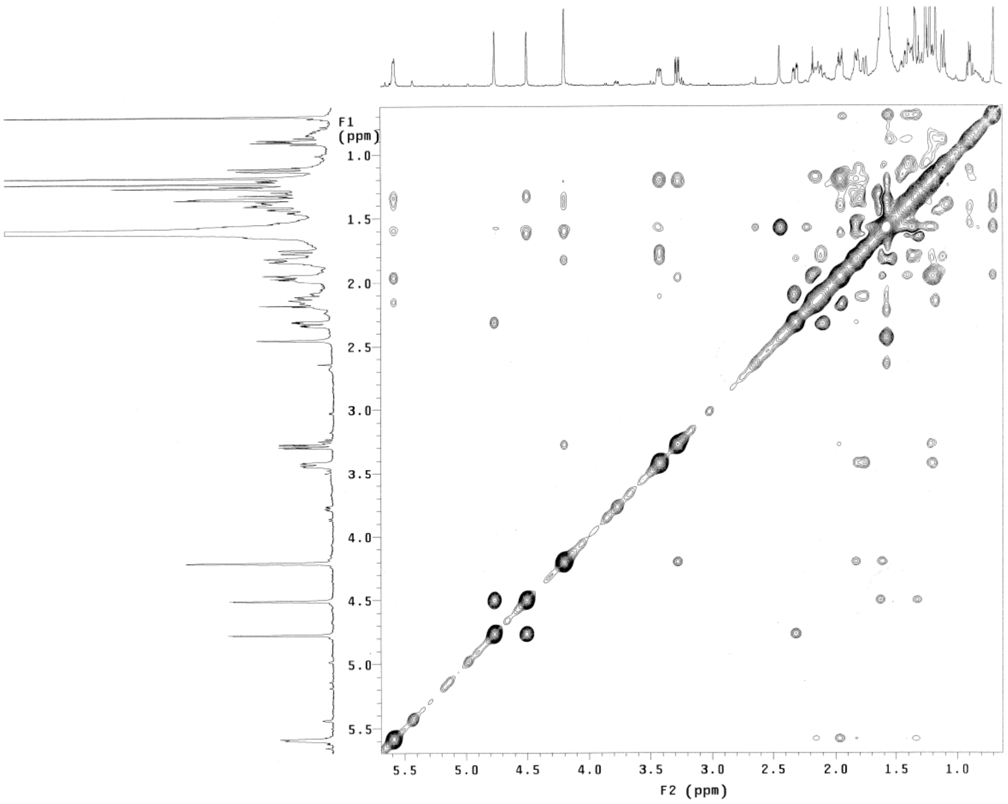

Figure S72. NOESY spectrum of **10** in $CDCl_3$.

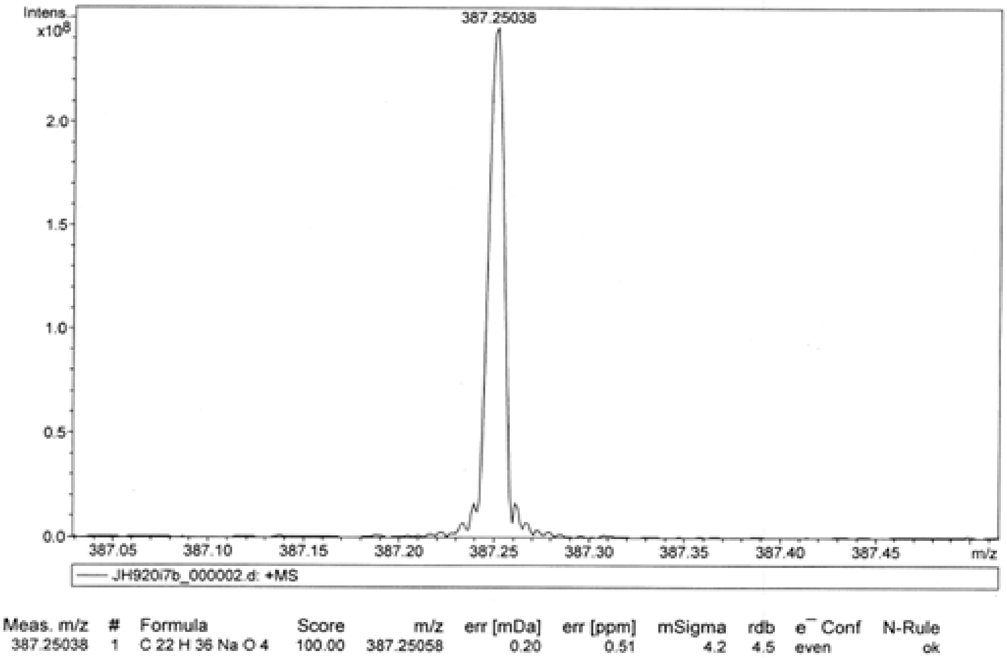

Meas. m/z	#	Formula	Score	m/z	err [mDa]	err [ppm]	mSigma	rdb	e⁻ Conf	N-Rule
387.25038	1	C 22 H 36 Na O 4	100.00	387.25058	0.20	0.51	4.2	4.5	even	ok

Figure S73. HRESIMS spectrum of **11**.

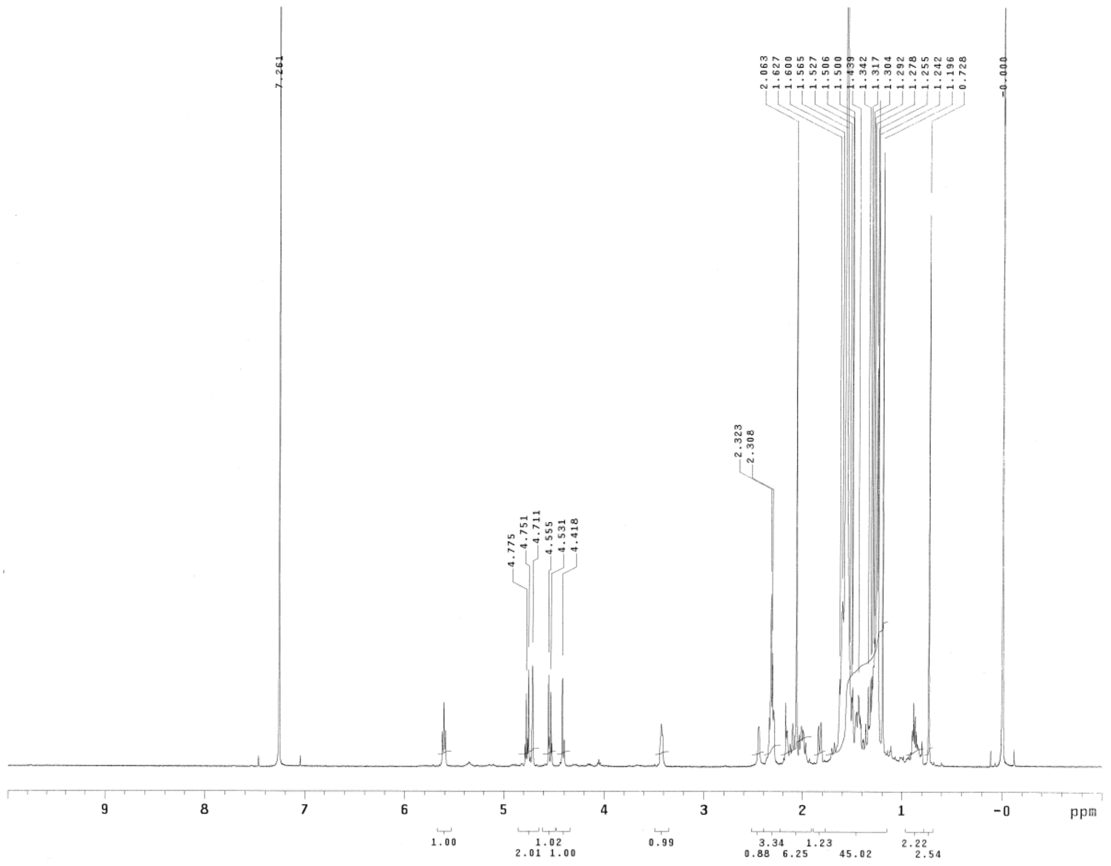

Figure S74. ^{1}H NMR spectrum of **11** in $CDCl_3$ at 500 MHz.

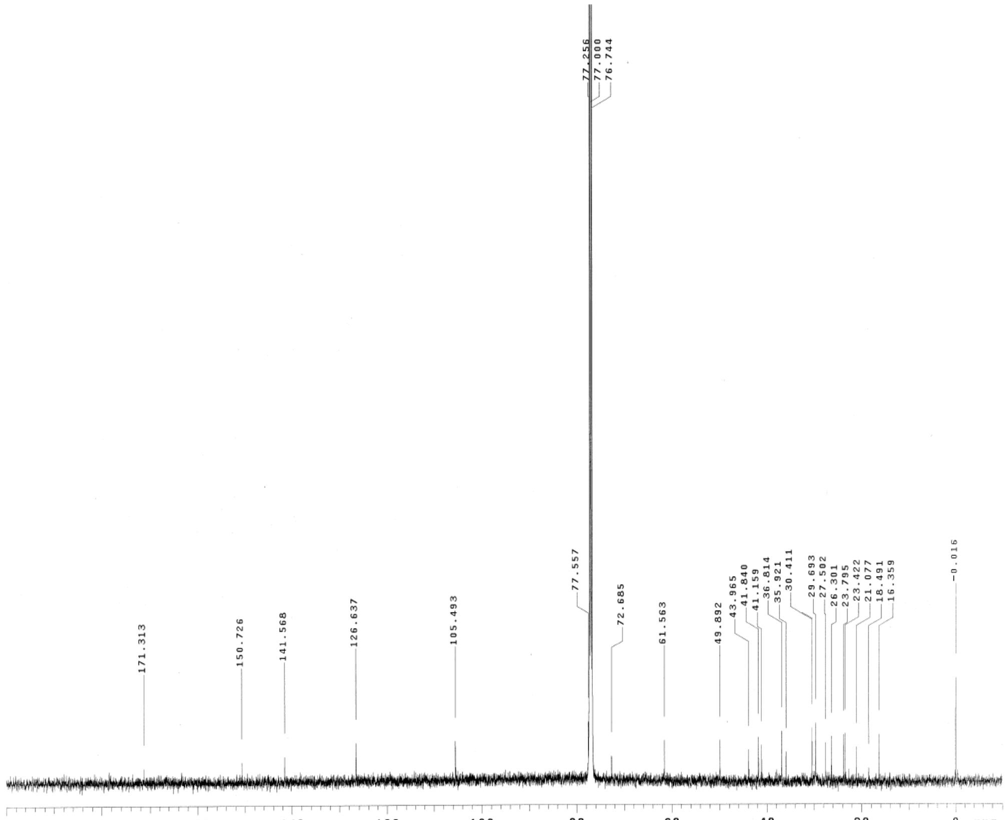

Figure S75. ^{13}C NMR spectrum of **11** in $CDCl_3$ at 125 MHz.

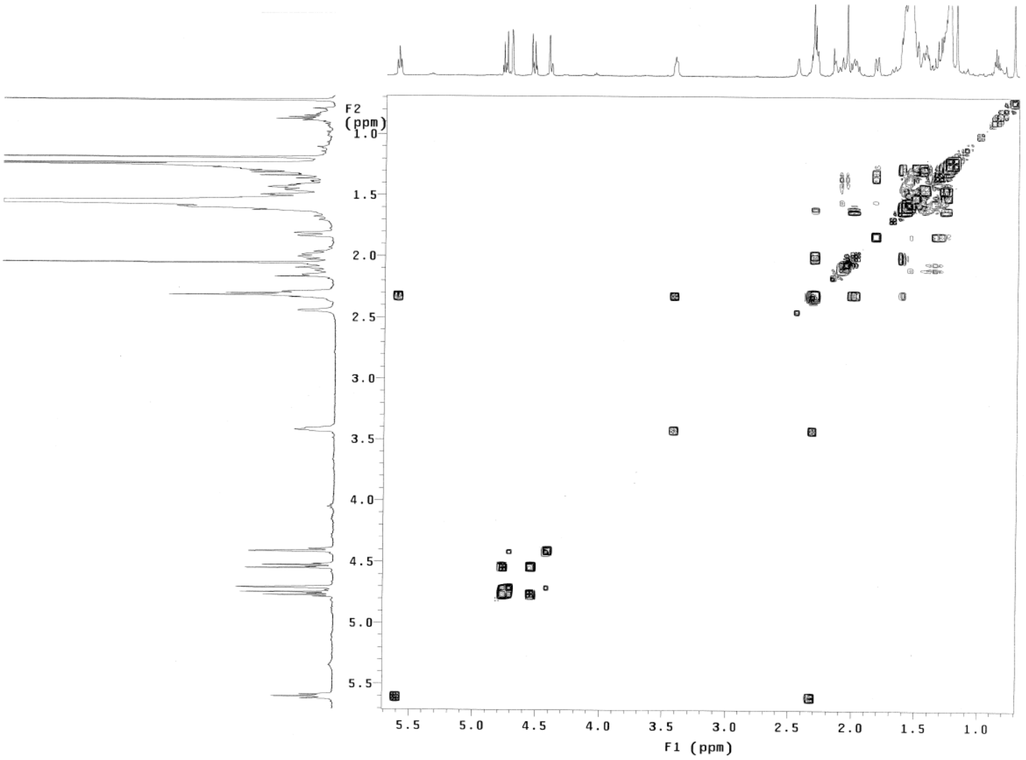

Figure S76. ^{1}H-^{1}H COSY spectrum of **11** in $CDCl_3$.

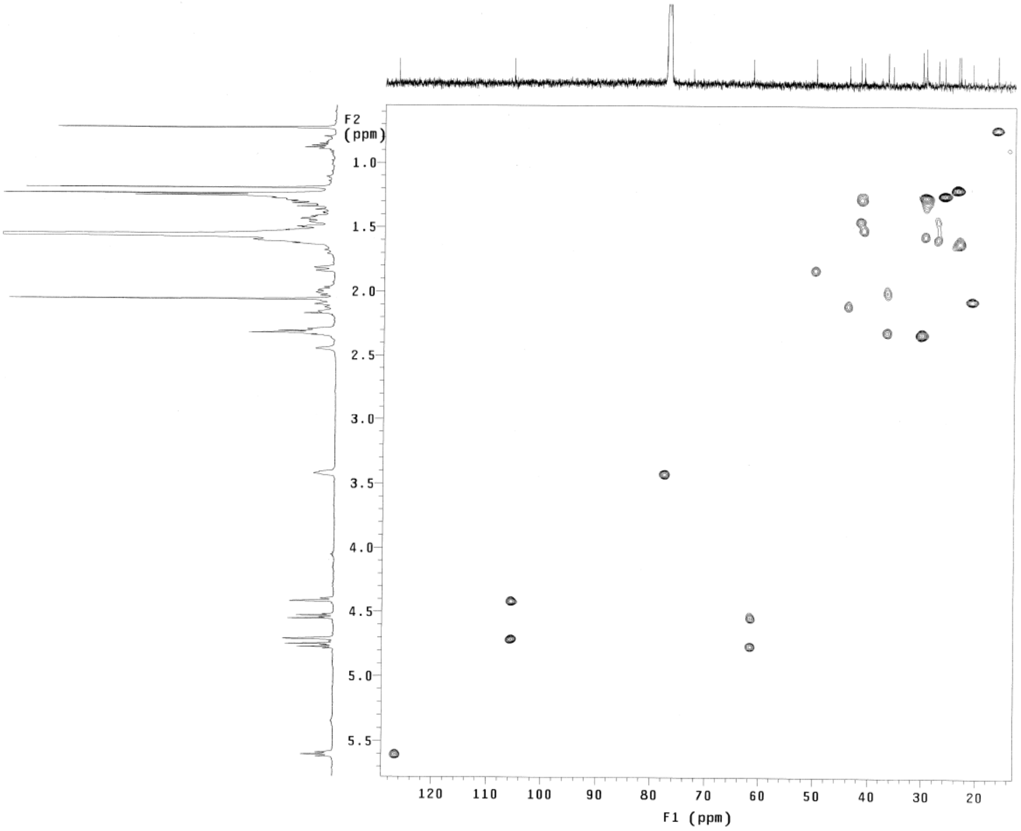

Figure S77. HSQC spectrum of **11** in $CDCl_3$.

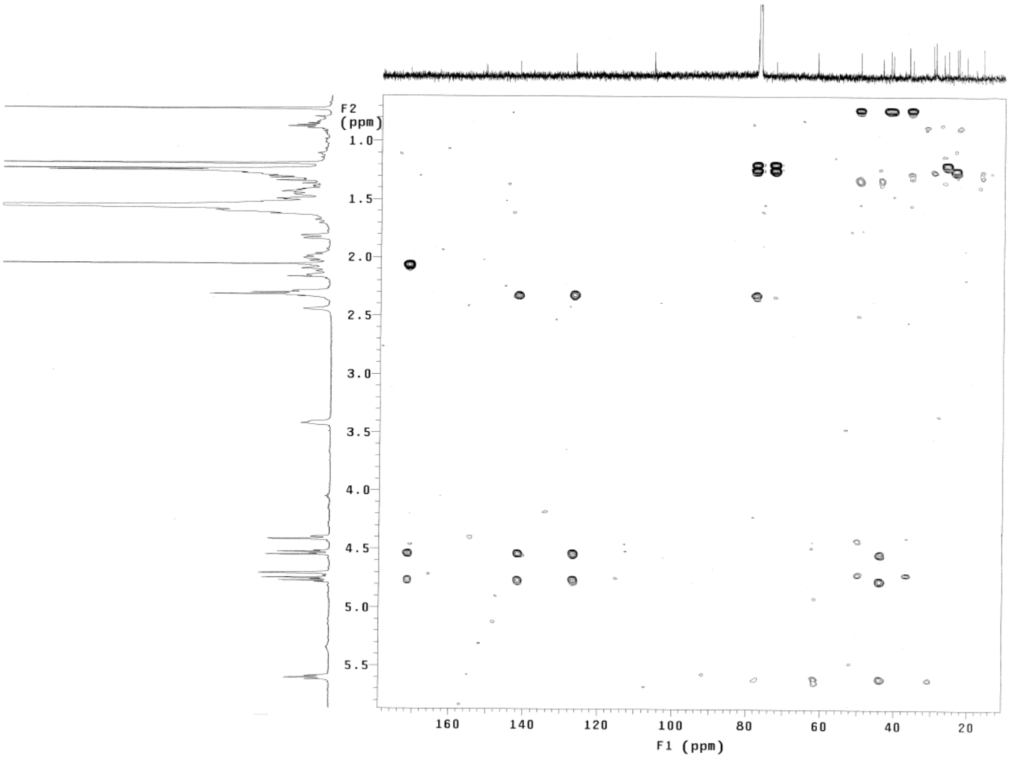

Figure S78. HMBC spectrum of **11** in $CDCl_3$.

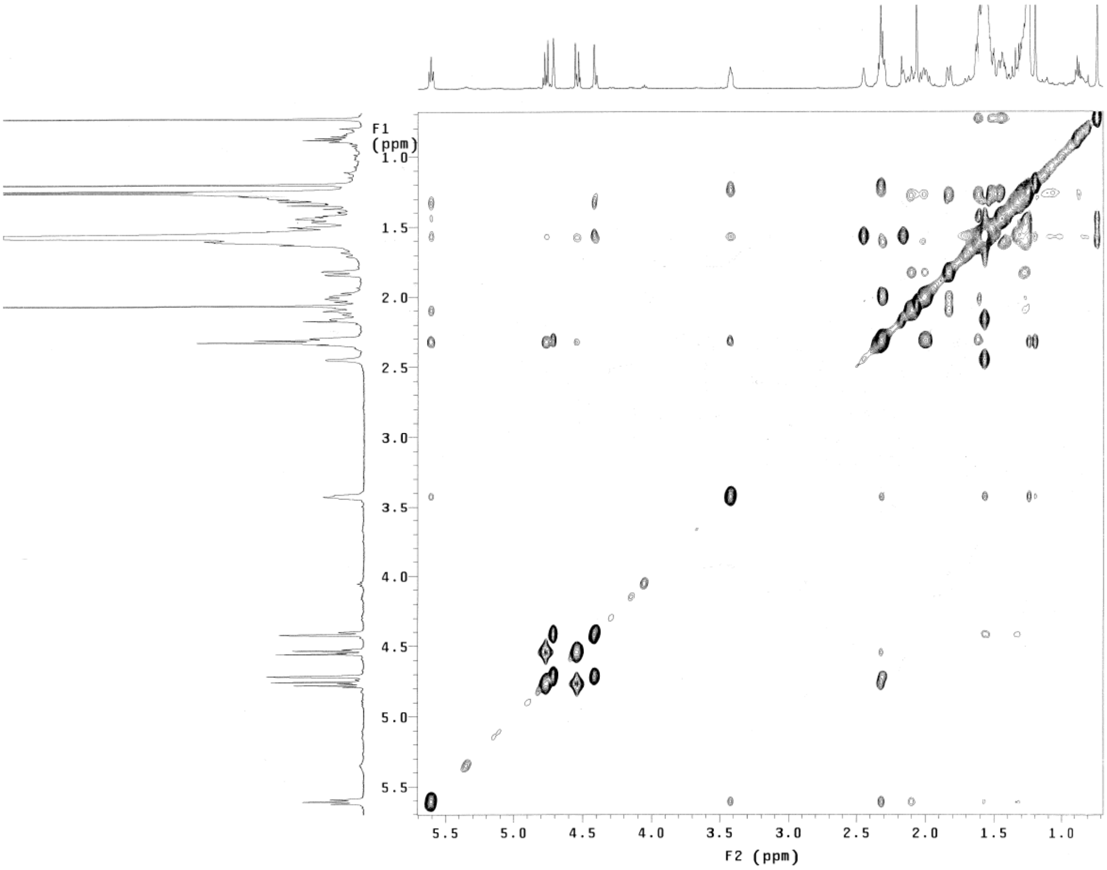

Figure S79. NOESY spectrum of **11** in $CDCl_3$.
